# Genetic Evidence of Cross-Border *Plasmodium vivax* Spread in a Malaria Pre-elimination Region of South Asia

**DOI:** 10.1093/infdis/jiag268

**Published:** 2026-05-15

**Authors:** Anjana Rai, Edwin Sutanto, Prakash Ghimire, Sonam Wangchuk, Tobgyel Drukpa, Mohammad Shafiul Alam, Najia Ghanchi, Awab Gulam Rahim, Angela Rumaseb, Hidayat Trimarsanto, Kian Soon Hoon, Nabaraj Adhikari, Sanjib Adhikari, Megha Raj Banjara, Komal Raj Rijal, Roshan Nepal, Ramesh Sharma Regmi, Bushra Qurashi, Shan-e-Zehra Zaidi, Mohammad Asim Beg, Benedikt Ley, Ric N Price, Kamala Thriemer, Sarah Auburn

**Affiliations:** Global and Tropical Health Division, Menzies School of Health Research and Charles Darwin University, Darwin, Northern Territory, Australia; Global and Tropical Health Division, Menzies School of Health Research and Charles Darwin University, Darwin, Northern Territory, Australia; Central Department of Microbiology, Tribhuvan University, Kirtipur, Kathmandu, Nepal; Public Health Laboratory, Department of Public Health, Ministry of Health, Thimphu, Bhutan; Vector Borne Disease Control Programme, Department of Public Health, Ministry of Health, Gelephu, Bhutan; Public Health Laboratory, Department of Public Health, Ministry of Health, Thimphu, Bhutan; Vector Borne Disease Control Programme, Department of Public Health, Ministry of Health, Gelephu, Bhutan; Infectious Diseases Division, International Centre for Diarrhoeal Disease Research, Bangladesh (ICDDR,B), Dhaka, Bangladesh; Department of Pathology and Laboratory Medicine, Aga Khan University, Karachi, Pakistan; Department of Public and Global Health, Afghan International Islamic University, Kabul, Afghanistan; Mahidol-Oxford Tropical Medicine Research Unit, Faculty of Tropical Medicine, Mahidol University, Bangkok, Thailand; Global and Tropical Health Division, Menzies School of Health Research and Charles Darwin University, Darwin, Northern Territory, Australia; Global and Tropical Health Division, Menzies School of Health Research and Charles Darwin University, Darwin, Northern Territory, Australia; Global and Tropical Health Division, Menzies School of Health Research and Charles Darwin University, Darwin, Northern Territory, Australia; Central Department of Microbiology, Tribhuvan University, Kirtipur, Kathmandu, Nepal; Central Department of Microbiology, Tribhuvan University, Kirtipur, Kathmandu, Nepal; Central Department of Microbiology, Tribhuvan University, Kirtipur, Kathmandu, Nepal; Central Department of Microbiology, Tribhuvan University, Kirtipur, Kathmandu, Nepal; Central Department of Microbiology, Tribhuvan University, Kirtipur, Kathmandu, Nepal; Central Department of Microbiology, Tribhuvan University, Kirtipur, Kathmandu, Nepal; Department of Pathology and Laboratory Medicine, Aga Khan University, Karachi, Pakistan; Department of Pathology and Laboratory Medicine, Aga Khan University, Karachi, Pakistan; Department of Pathology and Laboratory Medicine, Aga Khan University, Karachi, Pakistan; Global and Tropical Health Division, Menzies School of Health Research and Charles Darwin University, Darwin, Northern Territory, Australia; Global and Tropical Health Division, Menzies School of Health Research and Charles Darwin University, Darwin, Northern Territory, Australia; Mahidol-Oxford Tropical Medicine Research Unit, Faculty of Tropical Medicine, Mahidol University, Bangkok, Thailand; Centre for Tropical Medicine and Global Health, Nuffield Department of Clinical Medicine, University of Oxford, Oxford, United Kingdom; Global and Tropical Health Division, Menzies School of Health Research and Charles Darwin University, Darwin, Northern Territory, Australia; Global and Tropical Health Division, Menzies School of Health Research and Charles Darwin University, Darwin, Northern Territory, Australia; Mahidol-Oxford Tropical Medicine Research Unit, Faculty of Tropical Medicine, Mahidol University, Bangkok, Thailand

**Keywords:** malaria, South Asia, *Plasmodium vivax;* population structure, cross-border transmission, genotyping

## Abstract

**Background:**

*Plasmodium vivax* is the predominant cause of malaria in South Asia. *P. vivax* cases have fallen over the past decade, but cross-border transmission remains a major challenge to elimination. Genetic data can generate valuable insights into transmission; however, until now, only low-resolution data have been available from Nepal, Bhutan and Bangladesh. We piloted high-resolution genotyping using a new microhaplotype (multiallelic) assay to monitor *P. vivax* transmission across borders.

**Methods:**

Genotyping was conducted using the 93-microhaplotype vivaxGEN panel on *P. vivax* parasites collected from patients enrolled in clinical trials in Bangladesh, Bhutan, and Nepal between 2013 and 2023. These data were compared with open-access microhaplotype and genomic data derived from Afghanistan, India, and Pakistan between 2014 and 2024. Complexity and relatedness (identity by descent [IBD]) were determined within and between countries.

**Results:**

High-quality genotyping data were generated for Nepal (n = 19), Bhutan (n = 27), and Bangladesh (n = 35); comparative data were sourced from Afghanistan (n = 159), India (n = 24), and Pakistan (n = 213). High-complexity infections were observed in 27.04% (43/159) of isolates from Afghanistan, 16.43% (35/213) from Pakistan, and 28.57% (10/35) from Bangladesh. Low prevalence of high-complexity infections was observed in Bhutan (3.70%; 1/27) and Nepal (5.26%; 1/19), suggesting lower superinfection or co-infection. Country-wide IBD analyses revealed 3 genetic clusters partitioning Bangladesh and Bhutan (partial) from the remaining countries. There were 2 subpopulations in Bhutan, which separated local and cross-border imported cases.

**Conclusions:**

Our results highlight the use of regional high-resolution genetic data to enhance monitoring of transmission intensity and cross-border importations.

## Teaser

Genetic analysis of South Asian Plasmodium vivax isolates shows cross-border transmission contributing to ongoing endemicity. Continued investment in genetic infrastructure and data sharing is critical to utilize genomic epidemiology for timely public health responses to accelerate malaria elimination.

Malaria remains a major public health threat in South Asia, with more than 6 million cases reported in 2024; *Plasmodium vivax* accounted for approximately 60% of these cases, contributing substantially to the socio-economic burden [1]. India, Pakistan and Afghanistan remain highly endemic, accounting for almost 70% of all *P. vivax* malaria globally, whereas over the last decade, *P. vivax* cases have declined significantly in Nepal, Bhutan and Bangladesh [1].

Cross-border movement is frequent in South Asia, sustaining the reintroduction of *P. vivax* parasites into elimination areas such as the borders of Bhutan and Nepal. In Bhutan, there have been no local malaria cases since 2022; however, the southern border with India remains highly porous, with recent cases being imported, emphasizing the ongoing risk of parasite importation and resurgence [1, 2]. Nepal also shares a border with India to the east, south, and west, exposing the country to importation of parasites from malaria-endemic regions. Extensive cross-border movement of migrant workers returning from India to Nepal resulted in a high number of imported malaria cases; in 2023, these accounted for 98% of all *P. vivax* cases [1, 3]. Monitoring *P. vivax* transmission within and across borders is challenging owing in part to the biology of this species.

*P. vivax* can form dormant liver stages (hypnozoites), which reactivate weeks to months after initial infection causing recurrent blood-stage infections that facilitate ongoing transmission and a propensity for resurgence. Individuals with asymptomatic *P. vivax* infections act as the main reservoirs for transmission [4]. Furthermore, recent studies reveal that a substantial parasite biomass accumulates in the spleen, forming another hidden reservoir that likely sustains local transmission and cross-border spread [5]. Amidst this complexity, genetic data has potential to inform on parasite transmission dynamics.

Prior to our study, the genetic diversity and associated insights on *P. vivax* transmission and cross-border spread in South Asia remained poorly characterized. Previous investigations in Nepal, Bhutan and Bangladesh used genotyping at panels of up to 10 microsatellites to assess local *P. vivax* diversity [6-8]. While microsatellites are highly diverse, their genotyping costs are high, which limits panel size and associated resolution for detecting fine-scale population structure and relatedness [9, 10]. It is also challenging to collate microsatellite data from different studies unless consensus control samples and variant calling methods are applied prospectively [11-13]. This limits cross-country comparisons.

In this study, we present the first high-resolution *P. vivax* genetic datasets from Bhutan, Nepal and Bangladesh using a recently established 93-microhaplotype panel [13]. When these datasets were combined with existing data from 3 highly endemic countries in the region, the high-resolution marker panel permitted measures of identity-by-descent (IBD) to explore parasite-relatedness within and between countries. Our findings highlight the potential of this approach to track cross-border spread of *P. vivax* and provide important baseline data for ongoing surveillance of *P. vivax* transmission and spread across South Asia.

## METHODS

### Patient Samples From New Datasets

Parasite isolates were collected from patients enrolled in clinical studies conducted between 2013 and 2024 in 5 South Asian countries (Supplementary Table 1; Supplementary Figure 1). Samples from Nepal were collected from a clinical trial in Sudurpashchim Province, between 2021 and 2023, evaluating the safety and efficacy of a low-dose short-course primaquine treatment (3.5 mg/kg total dose given over 7 days) in glucose-6-phosphate dehydrogenase normal patients infected with *P. vivax* and *P. falciparum* (ClinicalTrials.gov ID: NCT04079621). Samples from Bhutan were collected during a therapeutic efficacy survey conducted from 2013 to 2015 in Sarpang, Gelephu, Trongsa, Wangdiphodrang, Trongsa, Pemagatshel, and Samdrupjongkhar districts [6]. Patients enrolled in the study were categorized into 2 groups based on nationality: Bhutanese nationals and non-nationals, to examine whether 93-microhaplotype assay can capture genetic separation between locally acquired and cross-border imported *P. vivax* infections. Samples from Bangladesh were collected during a prospective observational therapeutic efficacy study in the Chittagong Hill Tracts conducted between 2014 and 2015 [14].

DNA was extracted from blood samples using the Qiagen QIAamp DNA Blood Mini or Midi Kit, according to the manufacturer's instructions (Qiagen, Hilden, Germany). *Plasmodium* species were confirmed by PCR as previously described by Padley et al [15] with a modification suggested by Boonma et al [16] that each species is tested in a singleplex assay.

### Existing Data for Comparative Analysis

Amplicon sequencing and whole-genome sequencing (WGS) data from high-endemic countries in the region were included for comparative analysis. Amplicon sequencing data for Afghanistan were sourced from a previous study, which included samples collected during a clinical trial conducted from 2014 to 2017 in Jalalabad and Laghman (ClinicalTrials.gov ID: NCT01814683) [13, 17]. Amplicon sequencing data from Pakistan were generated using samples from a clinical trial (ClinicalTrials.gov ID: NCT04411836) conducted between 2023 and 2024 in Karachi and Thatta [18]. The samples from both clinical trials were collected from patients presenting with clinical symptoms, with *P. vivax* parasitemia confirmed by blood film microscopy. The amplicon sequencing data from Afghanistan and Pakistan were generated using the same library preparation methods applied to the new datasets from Bangladesh, Bhutan and Nepal for the vivaxGEN 93-microhaplotype panel [13]. Genetic data from India were obtained from an open-access WGS dataset generated using standard Illumina library preparation on genomic DNA (gDNA) samples, with Illumina sequencing generating 150 bp paired-end reads [19].

### Selective Whole-Genome Amplification

*Plasmodium vivax* selective whole-genome amplification (sWGA) was performed on samples from populations where pilot genotyping demonstrated low genotyping success in gDNA samples. sWGA was performed using 2 previously published primer sets, pvset1920 and pvset1 [20]. Amplifications were conducted separately with each primer set, and a pool of each sWGA products equal volume was also prepared for downstream library preparation.

### Amplicon Sequencing

Genomic DNA or sWGA product were used as input for library preparation using the vivaxGEN microhaplotype panel as previously described [13]. Briefly, libraries were prepared using a rhAmpSeq kit (Integrated DNA Technologies) with 5 μL template in 10 μL reactions to generate amplicons at the custom primer pool, followed by indexing. The resulting libraries were paired-end sequenced in multiplexes of up to 192 samples on an Illumina MiniSeq platform using the MiniSeq Mid Output kit (300 cycles).

### Variant Calling

Variant calling on the amplicon data was performed using the DADA2-based vivaxGEN MicroHaps pipeline (commit 359d042, https://github.com/vivaxgen/MicroHaps). The data were further processed to remove insertions and deletions (indels), and alleles with <2% proportion-wise or 5 read-pairs per sample. In the WGS data, SNP variants were initially called, requiring at least 5 reads to call an allele, with heterozygous calls if the minor allele had at least 2 reads and contributed to ≥5% of the total reads. Indel variants were removed, and then only microhaplotypes with no missing or heterozygous SNPs were retained. The amplicon and WGS data were combined and then samples with at least 60% (55/93) microhaplotype markers genotyped were included in downstream analyses.

### Population Genetics Analysis

Within-host diversity was measured by calculating the mean number of alleles per sample. Additionally, the effective Multiplicity of Infection (eMOI) metric was determined by MOIRE software (v3.5.0) [21] with samples pooled from all countries. The following parameters were used for MOIRE: burnin = 1000; pt_chains = seq(0.5, 1, length.out = 40); samples_per_chain = 1000; r_alpha = 3; r_beta = 3. eMOI is a metric of diversity that incorporates MOI and relatedness. Relatedness between samples was measured using Dcifer (v1.2.1) [22]. Pairwise identity by descent (IBD) was estimated in Dcifer with the assumption that paired strains between samples have the same relatedness. The total IBD between samples were then scaled by the minimum MOI between the 2 samples to obtain the overall relatedness. Network plots showing relatedness between different samples were created using ggnetwork (v0.5.13) [23].

The following analyses were conducted on a subset of high-complexity isolates. Isolates were categorized as high complexity if they have mean number of alleles >1.1. Rather than using 1.0, the 1.1 threshold was defined to account for a potential low level of sequencing error. Microhaplotype marker diversity and effective cardinality in the different countries was undertaken using paneljudge [24], with marker 64721 removed from paneljudge analysis. Median values >0.5 were considered indicative of high marker diversity. To confirm the population structure observed in Dcifer analysis, we used structure (v2.3.4) [25] with the following parameters: 10 000 burnin; 20 000 numreps; and 1 ploidy. Other parameters were left as default. We tested different K values (1 to 9) and performed 20 repeats for each K with randomized seeds. Sorting and plotting of the structure results were done using CLUMPAK [26]. The optimal number of clusters was calculated using the delta K method [27].

### Ethics Approval

Written, informed consent was provided by all patients or a parent or guardian. Ethical approvals for all studies included the use of blood samples for parasite genotyping and were provided by the Menzies School of Health Research Human Research Ethics Committee of the Northern Territory Department of Health and local ethics committees as listed in Supplementary Table 3.

## RESULTS

### High-Quality Microhaplotype Data Generated

Genotyping data were successfully generated for 96.4% (27/28) of samples from Bhutan, 89.7% (35/39) from Bangladesh, and 86.4% (19/22) from Nepal. Open-access data were obtained from 159 samples from Afghanistan, 24 from India and 213 from Pakistan. In total, high-quality amplicon sequencing data were generated on 453 independent samples, with additional WGS-derived data on 24 samples. High marker diversity was confirmed in all populations, providing high genetic resolution for diversity and structure analysis (Supplementary Figure 2).

A summary of the available patient demographics for the successfully genotyped samples is presented in Supplementary Table 2, and line-level sample details are presented in Supplementary Data 1. Variation was observed amongst the populations in gender, but in all populations, majority were male (range 60%–89%) (Supplementary Table 2). Aside from Afghanistan and Bangladesh, where a high proportion of individuals were in the 5–15-year-old category (34%–73%), >80% of patients were in the >15-year-old category.

### Low Complexity Observed in the Low-Endemic Populations

**Figure 1.**
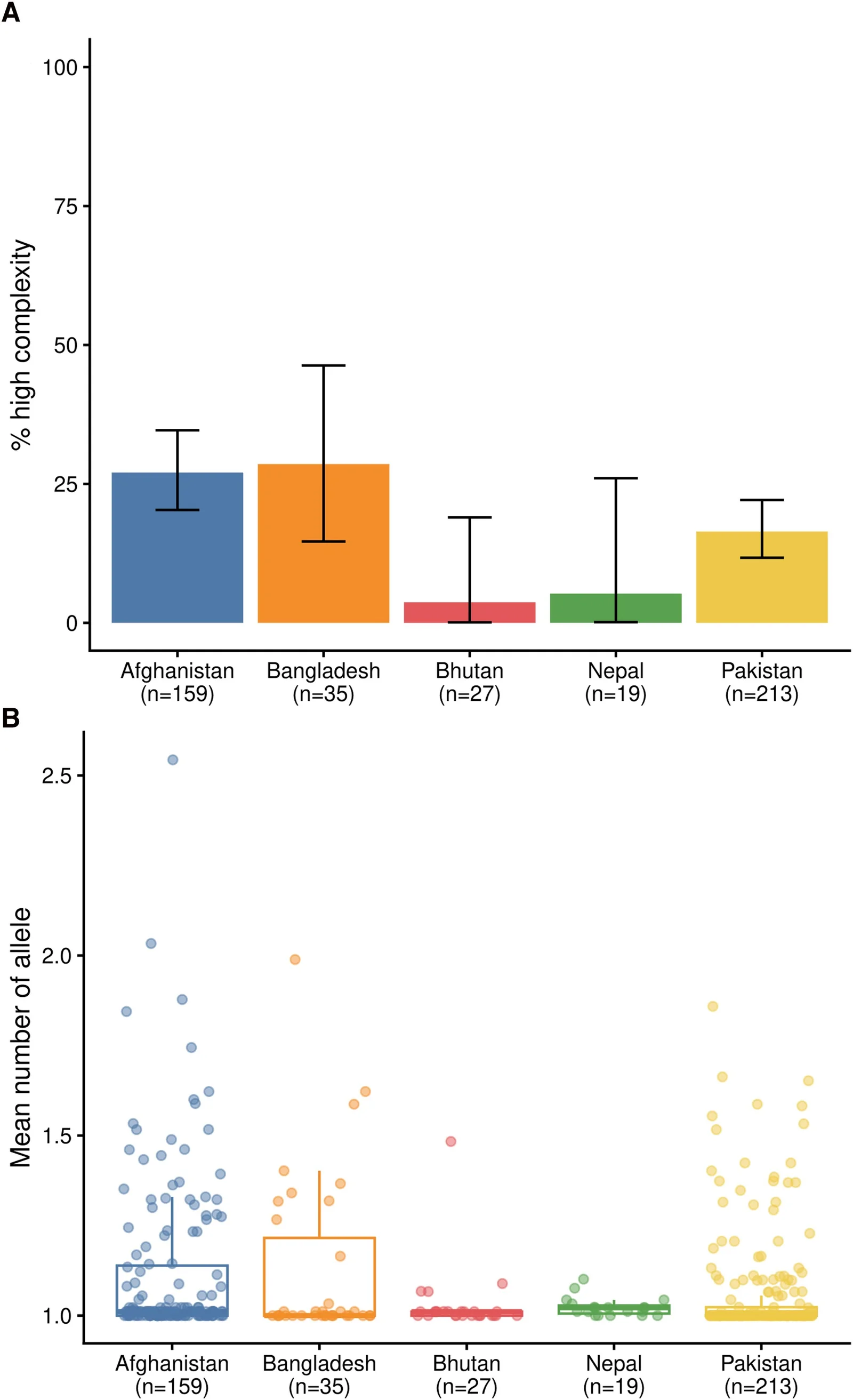
Low within-host infection diversity in low-endemic countries. *A*, presents the percentage of high-complexity infections, and *B*, presents the mean number of alleles distribution by country. India was excluded owing to the biased preselection for low-complexity genomic samples to enable accurate microhaplotype calling.

Complexity and within-host infection diversity can be used as a proxy to transmission intensity, with greater diversity generally observed in regions with higher transmission and associated superinfection and co-transmission [11]. For complexity estimation and within-host diversity analysis, the 24 WGS-derived cases from India were excluded from analysis as the cases were preselected to be monoclonal. A visual representation of the proportion of different alleles at each marker (termed rainbow plots) are presented for each infection in Bangladesh, Bhutan and Nepal (Supplementary Data 1). The data was also summarized quantitatively using measures, including the mean number of alleles for each sample. As illustrated in Supplementary Figure 3, amongst 453 independent samples with amplicon-based sequencing data, a modest inflection was observed at the 1.1 threshold defined for classifying infections as high complexity. High-complexity infections were observed in 27.04% (43/159) of isolates from Afghanistan, 16.43% (35/213) from Pakistan and 28.57% (10/35) from Bangladesh (Figure 1*A*). A low frequency of high-complexity infections was observed in Bhutan (3.70%; 1/27) and Nepal (5.26%; 1/19). In accordance with the binary high versus low-complexity classifications, the distribution of mean number of alleles per sample was lowest in Bhutan and Nepal (mean = 1.03 and 1.02, respectively), suggesting low superinfection or co-infection, consistent with low endemicity (Figure 1*B*). In contrast, the distributions of mean number of alleles were higher in Bangladesh (1.13), Afghanistan (1.12) and Pakistan (1.06), suggesting a higher incidence of superinfection and co-transmission (Figure 1*B*). A similar trend was observed with the eMOI distribution for all countries except Bangladesh, where the distribution was lower than that observed with the mean number of alleles (Supplementary Figure 4). Amongst 10 high complexity (mean number of alleles >1.1) samples from Bangladesh, 9(90%) were defined as single clone infections with MOIRE. Visual inspection of the rainbow plots for these cases confirmed high allelic diversity. Comparison of the mean number of alleles and eMOI showed moderate correlation in the measures, although the eMOI trended toward lower complexity values than the mean number of alleles, potentially reflecting sampling constraints (Supplementary Figure 5a). To further assess potential sampling effects, we performed 100 rounds of random subsampling with n = 19 (reflecting the smallest sample size in the study) from Afghanistan and Pakistan and computed the eMOI and mean number of alleles in each sample set. The random subsampling revealed that the mean eMOI estimates using MOIRE were more impacted by the sample size than the mean of mean number of alleles (Supplementary Figure 5b). We therefore used the latter measure in our interpretation of the data.

### Evidence of a Large Cross-Border Reservoir

**Figure 2.**
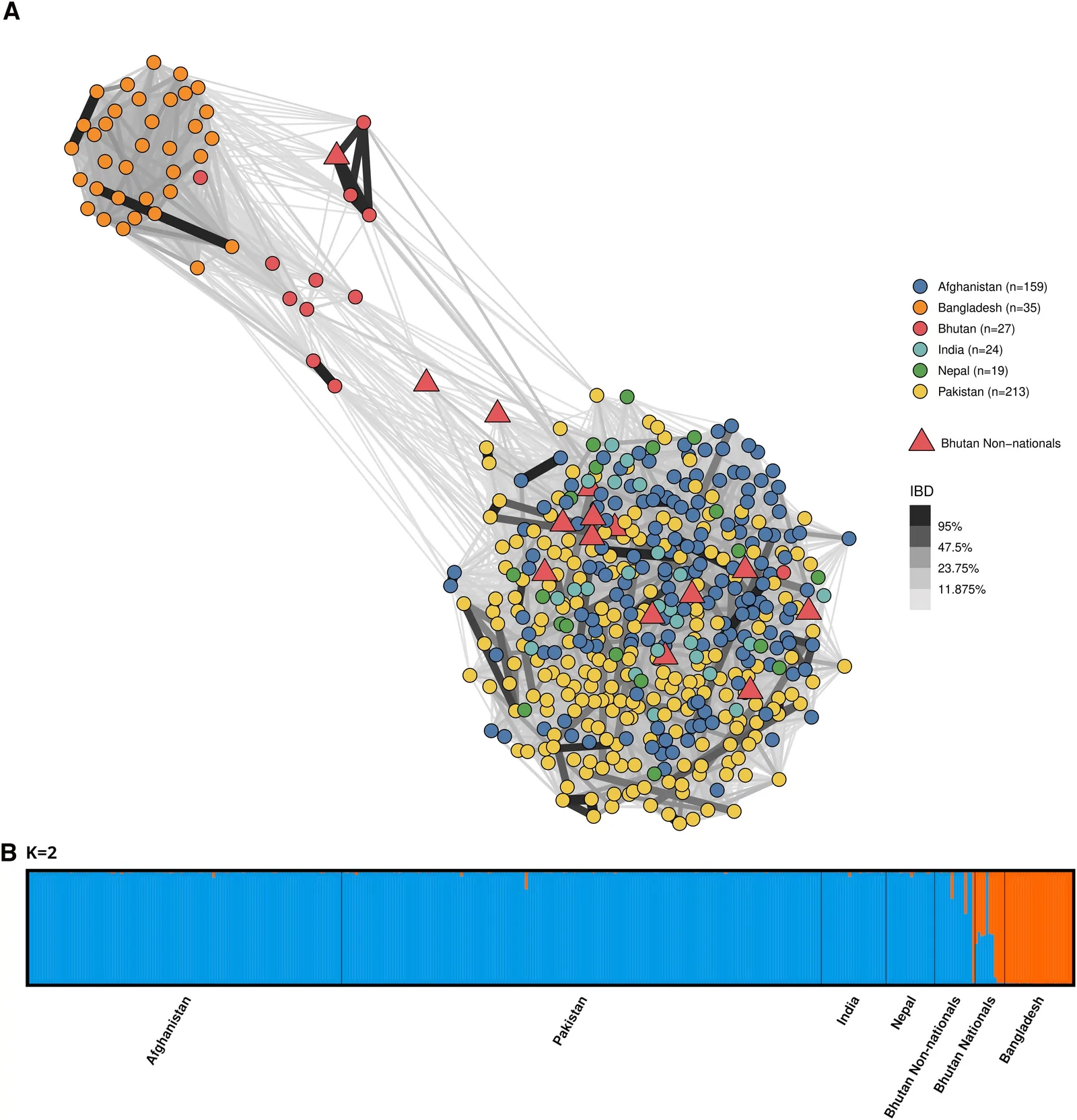
Regional population structure reveals cross-border transmission. *A*, presents an identity-by-descent-based (IBD-based) network illustrating connectivity between infections from Bhutan, Bhutan non-nationals (are of Indian origin, represented by a triangle shape), Bangladesh, Nepal, Afghanistan, India, and Pakistan. Each shape represents an infection and is color-coded by sites. For each country, connectivity, illustrated by a gray to thick black connecting line, is presented at the IBD threshold of ≥5% (thin grey lines) to ≥95% (thick black lines). *B*, Presents STRUCTURE-based population grouping across the 6 countries. The optimal number of clusters was determined using the delta K method, which showed the highest value at *K* = 2. Each bar represents an individual isolate with color-coding reflecting the ancestry to each of the 2 K groups. The Bangladesh isolates formed a distinct cluster (K2), while Afghanistan, Pakistan, India, Nepal, and Bhutan non-national isolates clustered together (K1). In contrast, the Bhutanese national isolates showed mixed ancestry to K1 and K2 or predominant K2 ancestry. Analysis was conducted on 477 and 387 independent infections for Dcifer and STRUCTURE, respectively.

Analysis of IBD was conducted at the regional level to inform on the relatedness between parasites from different countries. There were 3 distinct infection clusters in the IBD-network plot with IBD threshold of 5% (Figure 2*A*). The samples from Afghanistan, India, Pakistan and Nepal formed 1 large cluster, whilst the samples from Bangladesh formed a distinct cluster. The samples from Bhutan were divided into 2 subpopulations: 1 group formed its own cluster, while the other group clustered together with the multi-country cluser. Closer inspection of the Bhutanese subpopulations by patient nationality revealed that the distinct group largely comprised Bhutanese nationals, whilst the multi-country group largely comprised non-nationals, including Indian nationals presenting with clinical illness in Bhutan (Figure 2*A*).

Further investigation of population structure in the regional dataset was conducted using STRUCTURE software. The optimal number of clusters (K populations) [27] was identified as 2 (Supplementary Figure 6). 99.1% (348/351) of isolates from Afghanistan, Bhutanese non-nationals, India, Nepal and Pakistan had major ancestry to a large multi-country group (K1), whereas the 54.5% (6/11) of Bhutanese nationals showed mixed ancestry to both groups (Figure 2*B*). At *K* = 2, all (25) Bangladesh isolates had a majority (>80% ancestry) to a distinct cluster (K2) from isolates from the other South Asian populations (Figure 2*B*).

### Variation in Inbreeding Between Sites

**Figure 3.**
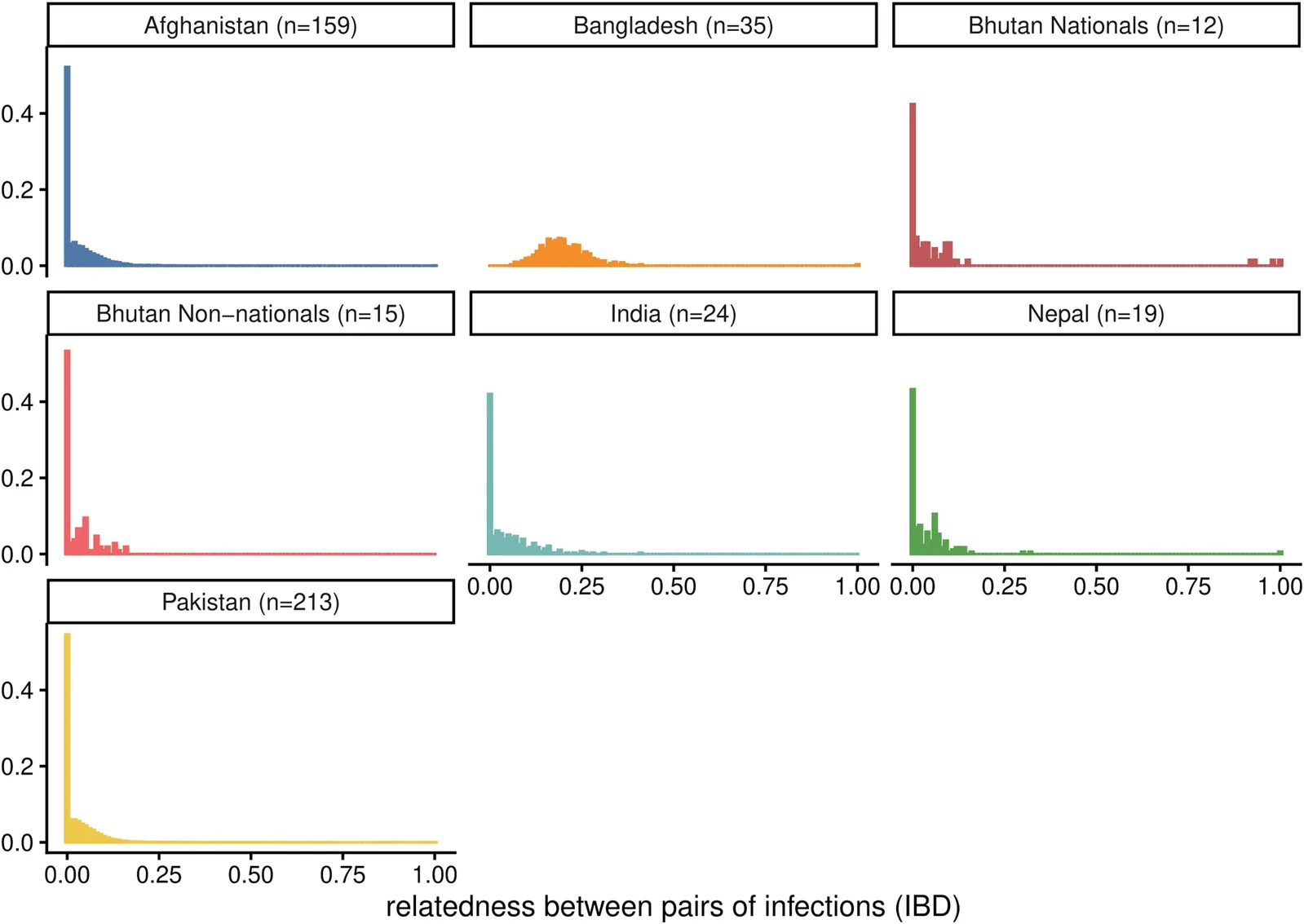
Variation in between-host infection relatedness by country. The histogram presents the distribution of the identity-by-descent (IBD) values for each country.

Analysis of within-country IBD was conducted to inform on the relatedness between parasites within countries. The samples from Bangladesh showed high relatedness (median IBD = 19.2%) compared with those from other countries included in the analysis. In contrast, Bhutan Nationals (median IBD = 1.5%), Bhutan Non-nationals (median IBD = 0%), and Nepal (median IBD = 2%) showed similar relatedness to those in the higher endemic settings (Figure 3).

### Demonstrated Strength of High- Versus Low-Resolution Markers to Capture Identity by Descent and Separate Local Versus Imported Cases in Bhutan

**Figure 4.**
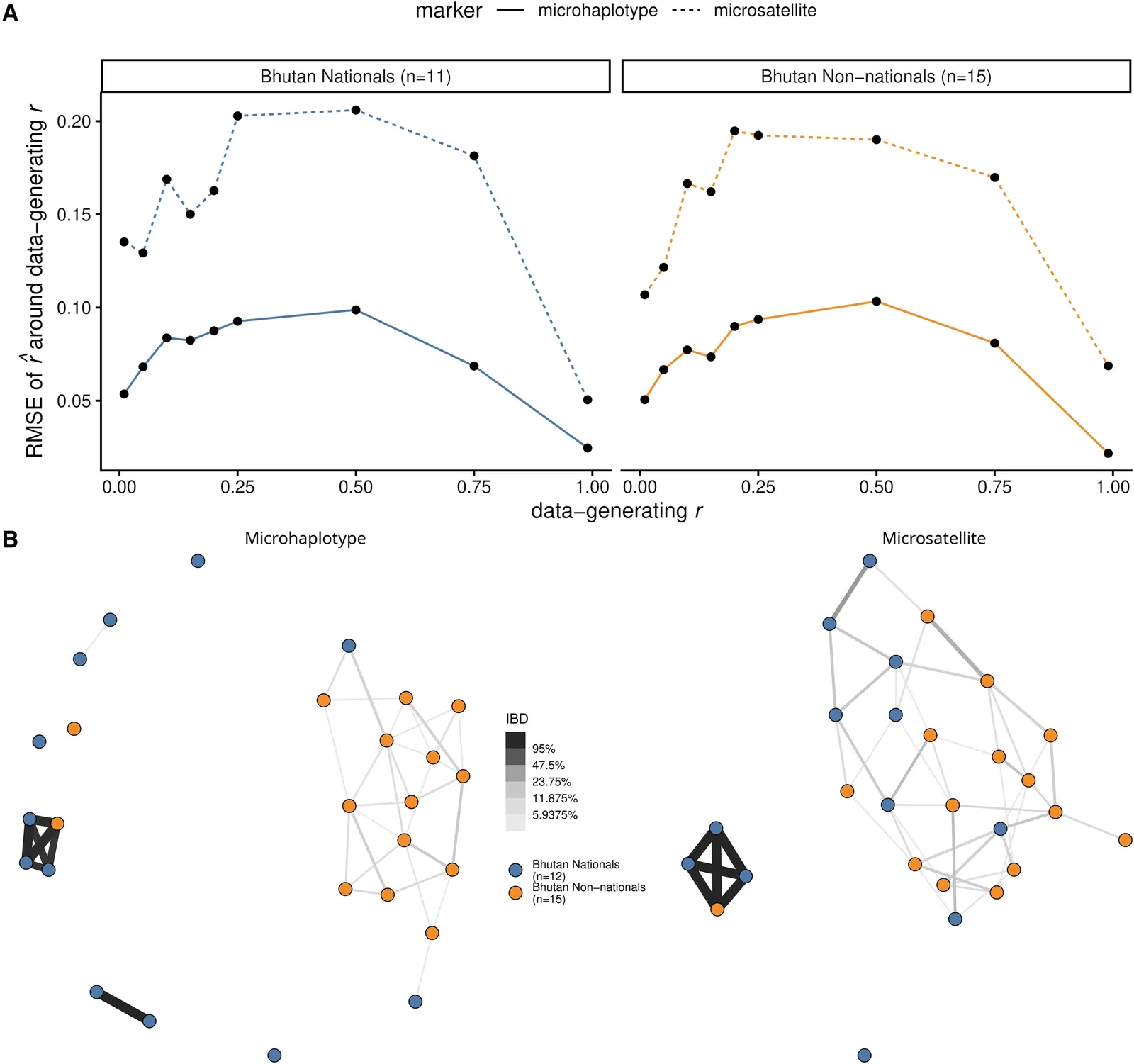
Higher IBD estimation accuracy in Bhutan using 92 microhaplotypes versus 9 microsatellites. *A*, presents the relative mean square error (RMSE) in estimation of the given IBD levels (0.01, 0.05, 0.1, 0.15, 0.20, 0.25, 0.5, 0.75, and 0.99) in the low-complexity Bhutanese national (n = 11) and non-national (n = 15) populations. At the data-generating *r* (IBD) of 0.5, the RMSE is approximately 2-fold higher for the microsatellites than the microhaplotypes in both populations. *B*, presents IBD-based cluster plots for each marker panel at minimum 3% relatedness using the Bhutanese sample set (12 national and 15 non-national cases). Greater distinction of non-national from national cases is observed with the microhaplotypes than the microsatellites.

Previous analyses using a panel of 9 microsatellites did not reveal genetic separation between locally acquired and cross-border imported *P. vivax* infections in Bhutan [6]. We explored whether the separation observed with the microhaplotype assay (92 markers analyzed) was due to the panels' capacity to capture IBD accurately. Paneljudge software was used to assess the diversity of the 2 marker panels and their accuracy in predicting different levels of IBD between sample pairs in Bhutan. Although the microsatellites had a higher average marker diversity and effective cardinality compared with the microhaplotypes (Supplementary Figure 7a and 7b), the relative mean square error of the microsatellites was up to 2-fold higher than for the microhaplotypes (Figure 4*A*). Identity by descent networks illustrated the relative limitation of the microsatellite panel, with almost all samples (national and non-national) showing relatedness at approximately 12% (Figure 4*B*). In contrast, the microhaplotypes showed greater discrimination of the Bhutanese non-nationals (related at approximately 3%), indicating the panels greater ability to resolve low relatedness (Figure 4*B*). Overall, the data suggested that 92 microhaplotypes could estimate IBD with significantly greater accuracy than the 9-microsatellite assay. Even without reference data from other countries, the microhaplotype panel achieved greater differentiation of local versus imported infections in Bhutan.

## DISCUSSION

Our study presents novel genetic data that serve as a foundation for the molecular surveillance of *P. vivax* in South Asia. Our high-resolution genetic data were derived from parasite isolates collected across multiple countries in the region, enabling a detailed investigation of *P. vivax* cross-border transmission and highlighting the risks for malaria resurgence in pre-elimination countries. The study also demonstrates the power of microhaplotype genotyping to distinguish local from cross-border importations.

Genetic diversity indices can be a useful tool in gauging the intensity of *P. vivax* transmission [28-31]. However, software that rely on population-level allele frequency information, such as MOIRE, may not be effective in low-endemic settings where sample size is inevitably small. Our data revealed deviations in within-host diversity estimates derived from MOIRE relative to population-independent measures of within-sample allele frequency. Visual inspections of the data and subsampling tests revealed that the population-independent measures were generally more accurate gauges of within-sample diversity. This highlights a critical tool selection consideration for molecular surveillance in pre-elimination settings.

Using the population-independent measures of within-host diversity, our genetic data from Bhutan and Nepal aligned with epidemiological data on annual parasite incidence (API), suggesting low transmission characterized by low prevalence of high-complexity infection and indicative of infrequent superinfection or co-infection. The low prevalence of high-complexity infections is not a limitation of the marker diversity, as this was high in all populations assessed. Conversely, there was a high frequency of high-complexity infections in Afghanistan and Pakistan, where API was high. Interestingly, Bangladesh also had a high frequency of high-complexity infections despite low reported API. In contrast to Bhutan, that have reported 3 years of zero indigenous cases, and Nepal reported a low number of indigenous cases in the past 5 years, Bangladesh remains endemic for *P. vivax*. Investigation of the subpatent and asymptomatic reservoir of infection in Bangladesh may shed more light on the high within-host diversity.

Notable differences in population-level genetic relatedness were observed between countries. Samples from Bangladesh exhibited high relatedness between infections, suggesting high inbreeding. In contrast, samples from Bhutan (even when restricted to isolates collected from nationals) and Nepal showed low relatedness between infections, which could reflect the modest sample sizes in these populations. Alternatively, the influx of imported cases may sustain local diversity in these populations. In Bhutan, the country-wide spatial and long temporal sampling catchment could contribute to the low relatedness between infections. The mountainous terrain in Bhutan may reduce human movement between different provinces, which could lead to a highly structured local parasite population. Despite the overall low relatedness, a few highly related infections were observed across different countries. Most of the closely related infections originated from the same catchment areas, likely reflecting local clonal expansion. In Bhutan, 1 infection that was highly related was identified from a non-national individual, which is likely to represent either infections in the country or imported cases subsequently spread within the community. Our analyses revealed apparent relatedness among *P. vivax* populations within and across geographically proximate border regions. These patterns highlight that while local transmission persists within each country, cross-border movement of parasites also remains a contributor to ongoing transmission.

Previous genetic surveys in Nepal, Bhutan, and Bangladesh have primarily employed panels of up to 10 microsatellite markers [7, 8, 32]. However, as our analysis in Bhutan demonstrated, small microsatellite panels have low-resolution information and are unable to measure IBD accurately, constraining distinction between local and imported cases in porous border regions. In contrast, the microhaplotype-based IBD analysis in Bhutan successfully distinguished locally acquired infections from those genetically linked to parasites from neighboring countries. This highlights the utility of microhaplotype genotyping in improving the detection of cross-border importations.

In contrast to Bhutan, Nepal had limited evidence of within-country population structure or differentiation from highly endemic areas in Afghanistan, India and Pakistan. This may reflect high parasite diversity sustained by cross-border importations from neighboring populations. Future work combining genetic with epidemiological data in model frameworks to reconstruct transmission chains, and conducting source-sink mapping, could provide even clearer insights into cross-border transmission dynamics in areas such as Nepal [33]. Future work should also use available high-resolution microhaplotype data from high-endemic countries to gain deeper insights into local heterogeneity and genetic diversity.

Our study was limited by using retrospective sample collections and a relatively small number of samples from several countries. The years of sample collection and catchment areas also differed between studies. However, in Bhutan and Nepal, which had the smallest sample size, the main constraint was the local endemicity: although small, the samples represent a significant proportion of all national cases. Despite the limitations, the data provides important proof-of-concept and baseline data for future surveillance in the region.

## CONCLUSION

Our findings demonstrate that high-resolution *P. vivax* genotyping provides a feasible and cost-effective approach for tracking intervention efficacy and cross-border spread. Continued investment in genetic infrastructure, capacity building, and collaborative data sharing will be critical to fully utilize the potential of genomic epidemiology to inform timely public health responses that will accelerate malaria elimination in South Asia and beyond.

## Supplementary Material

jiag268_Supplementary_Data

## Supplementary Data

Supplementary materials are available at *The Journal of Infectious Diseases* online (http://jid.oxfordjournals.org/). Supplementary materials consist of data provided by the author that are published to benefit the reader. The posted materials are not copyedited. The contents of all supplementary data are the sole responsibility of the authors. Questions or messages regarding errors should be addressed to the author.
