# Supplementary material for: Genetic Evidence of Cross-Border Plasmodium vivax Spread in a Malaria Pre-elimination Region of South Asia
Source: J Infect Dis. 2026 May 15;234(3):e671–80. doi: 10.1093/infdis/jiag268 (PMC13600386; doi:10.1093/infdis/jiag268)
Supplement: Supplementary file 1 [file jiag268_supplementary_data.zip › Revision1.0_RaiAnjana_Supplementary information.docx]

**Supplementary information**

**Supplementary Figure 1. Geographic location of the study sites from which data were generated.** The large base map illustrates the countries where samples were derived from, including Nepal, Bhutan, Bangladesh, Afghanistan, Pakistan, and India. The smaller map illustrates the multiple divisions within Bhutan where samples were collected from. This map was generated using the Malaria Atlas Project tools and presents information on *P. vivax* clinical counts in 2014 (<https://data.malariaatlas.org/maps>).

**Supplementary Figure 2. Marker diversity by country.** Microhaplotype marker diversity was analysed for samples from different countries using paneljudge [1]. Each box represents a country. Individual dots represent diversity estimates for each marker. Analysis was conducted on a total of 387 independent, **low-complexity** infections.

**Supplementary Figure 3. Mean number of alleles across all populations**. A threshold of 1.1 was determined (red dashed line) based on the observed inflection.

**Supplementary Figure 4: Effective multiplicity of infection (eMOI) distribution by country.** India was excluded owing to the biased pre-selection for low-complexity genomic samples to enable accurate microhaplotype calling.

**Supplementary Figure 5**. Panel a) shows the correlation between effective multiplicity of infection (eMOI) and mean number of alleles. Samples from each country are colour coded. Panel b) shows the correlation between the mean of eMOI and the mean number of alleles from 100 rounds of random subsampling with n=19 from Afghanistan (left) and Pakistan (right). The blue dotted line represents the mean of mean number of alleles from the entire sample set, and the red dashed line represents the mean of eMOI using all samples.

**Supplementary Figure 6. The optimal number of clusters (K).** The optimal number of clusters was determined to be K = 2, as calculated by CLUMPAK [2], as previously described [3].

**Supplementary Figure 7. Marker diversity of the two marker panels.** Panel a) shows heterozygosity measures for Bhutan Nationals and Non-national cases, and panel b) shows effective cardinality scores in 11 independent low complexity Bhutan-National and 15 independent low complexity Bhutan Non-National samples. Each box plot shows the median, interquartile range, and minimum and maximum values for heterozygosity (panel a) and effective cardinality (panel b) calculated using paneljudge [1].

**Supplementary Table 1. Study site details**

| **Site name** | **Country** | **State/Division** | **Enrolment framework** | **Enrolment period** | **State/Province level *P. vivax* API 2014, 2024*** |
| --- | --- | --- | --- | --- | --- |
| Tikapur | Nepal | Sudurpaschim | Clinical trial | Aug 2021 - Aug 2023 | 0.68, 0.02 |
| Malakheti | Nepal | Sudurpaschim | Clinical trial | Aug 2021 - Aug 2023 | 0.68, 0.02 |
| Alikadam | Bangladesh | Chittagong Hill Tracts | Cross-sectional survey | Aug 2015 – Jan 2016 | 0.70, 1.17 |
| Laghman | Afghanistan | Laghman | Clinical trial | July 2014 - Nov 2017 | 32.72, 32.06 |
| Jalalabad | Afghanistan | Nangarhar | Clinical trial | July 2014 - Nov 2017 | 48.03, 43.13 |
| Karachi | Pakistan | Sindh | Clinical trial | April 2023 – Sept 2024 | 9.83, 66.36 |
| Thatta | Pakistan | Sindh | Clinical trial | April 2023 – Sept 2024 | 9.83 66.36 |
| Sarpang tar/Rabdeyling/Jambeling/Chubarthang/Chokhorling/Umling | Bhutan | Sarpang | Clinical efficacy | April 2013 – Oct 2015 | 0.54, 0 |
| Tsirang | Bhutan | Tsirang | Clinical efficacy | April 2013 – Oct 2015 | 0.1, 0 |
| Kamichu | Bhutan | Wangdiphodrang | Clinical efficacy | April 2013 – Oct 2015 | 0**, 0 |
| Sherubling | Bhutan | Trongsa | Clinical efficacy | April 2013 – Oct 2015 | 0.04, 0 |
| Mgalam | Bhutan | Pemagatshel | Clinical efficacy | April 2013 – Oct 2015 | 0**, 0 |
| Sethi | Bhutan | Samdrup Jongkhar | Clinical efficacy | April 2013 – Oct 2015 | 0**, 0 |

*API, Annual Parasite Incidence (cases/1000) for 2014 and 2024, derived from Malaria Atlas Project. **<0.001

**Supplementary Table 2. Demographic details of the patient samples that were successfully genotyped**

| Site | Country | Collection period | Age category (years) | | | % Male patients |
| --- | --- | --- | --- | --- | --- | --- |
|  |  |  | <5 | 5-15 | >15 |  |
| Tikapur | Nepal | Oct 2021 - Aug 2023 | 0/14 (0%) | 0/14 (0%) | 14/14 (100%) | 85.7% (12/14) |
| Malakheti | Nepal | Oct 2021 - Aug 2023 | 0/5 (0%) | 1/5 (20%) | 4/5 (80%) | 80.0% (4/5) |
| Bhutan | Bhutan | May 2013 - Apr 2015 | 0/27 (0%) | 3/27 (11.1%) | 24/27 (88.9%) | 88.9% (24/27) |
| Chittagong Hill Tracts | Bangladesh | Aug 2014 - Jan 2015 | 2/35 (5.7%) | 12/35 (34.3%) | 21/35 (60%) | 60.0 % (21/35) |
| Laghman | Afghanistan | Sep 2014 - Apr 2016 | 3/30 (10%) | 22/30 (73.3%) | 5/30 (16.7%) | 66.7% (20/30) |
| Jalalabad | Afghanistan | Apr 2015 - Apr 2016 | 13/129 (10.1%) | 53/129 (41.1%) | 63/129 (48.8%) | 75.2 % (97/129) |
| Karachi | Pakistan | May 2023 - Mar 2024 | 0/22 (0%) | 0/22 (0%) | 22/22 (100%) | 72.7% (16/22) |
| Thatta | Pakistan | Apr 2023 -Mar 2024 | 0/191 (0%) | 0/191 (0%) | 191/191 (100%) | 73.3% (140/191) |

**Supplementary Table 3. List of Ethics Approvals**

| Country | Ethics review board/regulatory authorities | Reference number |
| --- | --- | --- |
| Australia/Nepal | Human Research Ethics Committee of the Northern Territory Department of Health and Menzies School of Health Research | HREC-2019-3467 |
|  | Nepal Health Research Council | 857/2019 P |
| Australia/Bhutan | Human Research Ethics Committee of the Northern Territory Department of Health and Menzies School of Health Research | HREC-2012-1871 |
|  | Research Ethics Board of Health, at the Ministry of Health in Bhutan | REBH 2012/031 |
| Australia/Bangladesh | Human Research Ethics Committee of the Northern Territory Department of Health and Menzies School of Health Research | HREC-2014-2228 |
|  | Ethics Review Committee of the icddr,b | PR-14053 |
| Australia/Afghanistan | Human Research Ethics Committee of the Northern Territory Department of Health and Menzies School of Health Research | 13–1991 |
|  | Oxford Tropical Research Ethics Committee OxTREC | 1014–13 |
|  | Ministry of Public Health, Afghanistan |  |
| Australia/Pakistan | Human Research Ethics Committee of the Northern Territory Department of Health and Menzies School of Health Research | HREC-20-3694 |
|  | Ethics Review Committee of the Aga Khan University | 2022-7042-20769 |
|  | National Institutes of Health, Health Research Institute, National Bioethics Committee (NBC) | 4-87/NBC-772/22/1833 |
|  | Drug Regulatory Authority of Pakistan | 16-37/2022 DD (PS) |

**References**

1. LaVerriere E, Schwabl P, Carrasquilla M, et al. Design and implementation of multiplexed amplicon sequencing panels to serve genomic epidemiology of infectious disease: A malaria case study. Molecular Ecology Resources **2022**; 22:2285-303.

2. Kopelman NM, Mayzel J, Jakobsson M, Rosenberg NA, Mayrose I. Clumpak: a program for identifying clustering modes and packaging population structure inferences across K. Molecular Ecology Resources **2015**; 15:1179-91.

3. Evanno G, Regnaut S, Goudet J. Detecting the number of clusters of individuals using the software STRUCTURE: a simulation study. Mol Ecol **2005**; 14:2611-20.
