# Supplementary material for: Genetic Evidence of Cross-Border Plasmodium vivax Spread in a Malaria Pre-elimination Region of South Asia
Source: J Infect Dis. 2026 May 15;234(3):e671–80. doi: 10.1093/infdis/jiag268 (PMC13600386; doi:10.1093/infdis/jiag268)
Supplement: Supplementary file 1 [file jiag268_supplementary_data.zip › Supplementary_Data_1.pdf]

NE−003 (Mean number of alleles = 1.02; eMOI = 1)

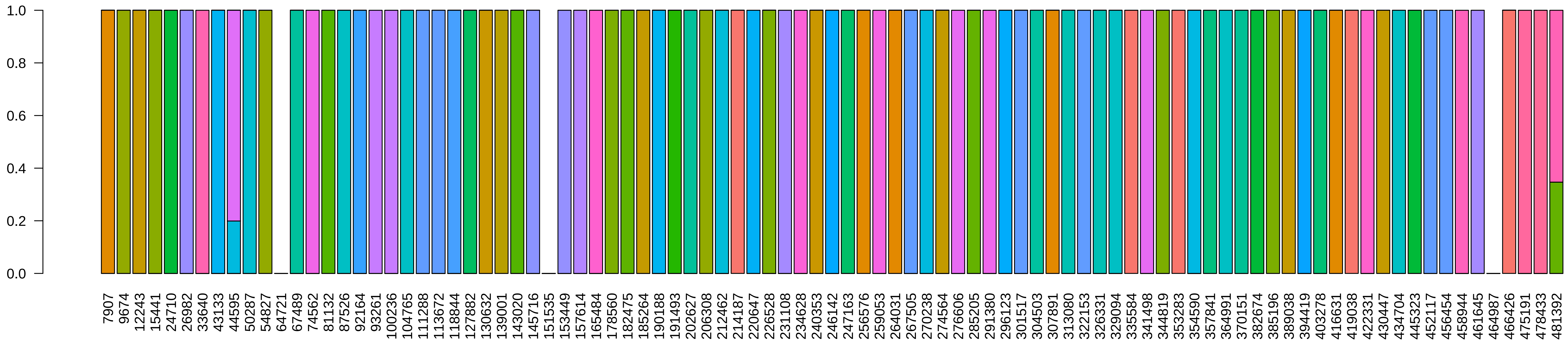

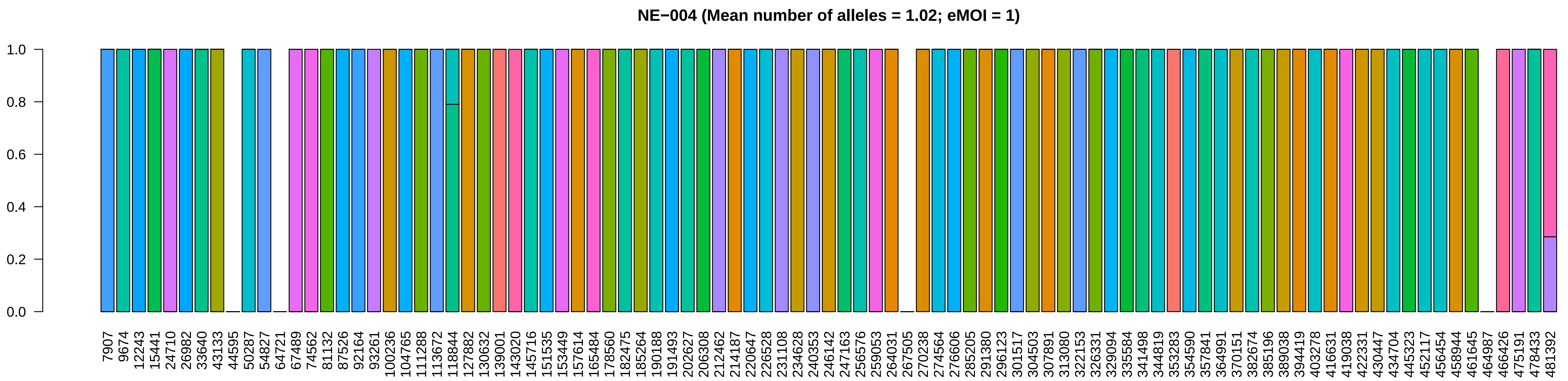

NE−004 (Mean number of alleles = 1.02; eMOI = 1)
1.0
0.8
0.6
0.4
0.2
0.0
7907 9674 12243 15441 24710 26982 33640 43133 44595 50287 54827 64721 67489 74562 81132 87526 92164 93261 100236 104765 111288 113672 118844 127882 130632 139001 143020 145716 151535 153449 157614 165484 178560 182475 185264 190188 191493 202627 206308 212462 214187 220647 226528 231108 234628 240353 246142 247163 256576 259053 264031 267505 270238 274564 276606 285205 291380 296123 301517 304503 307891 313080 322153 326331 329094 335584 341498 344819 353283 354590 357841 364991 370151 382674 385196 389038 394419 403278 416631 419038 422331 430447 434704 445323 452117 456454 458944 461645 464987 466426 475191 478433 481392

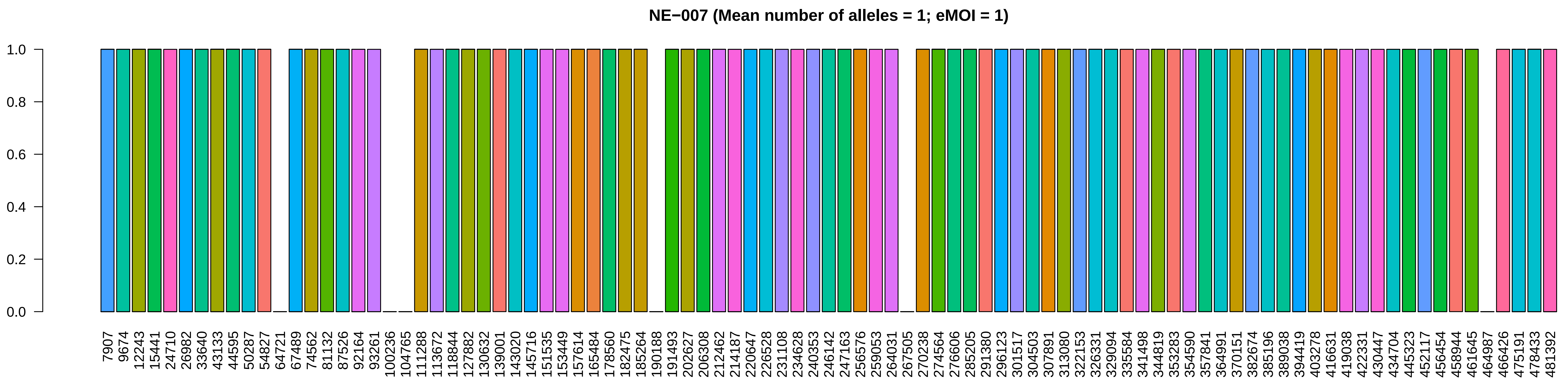

NE−007 (Mean number of alleles = 1; eMOI = 1)
1.0
0.8
0.6
0.4
0.2
0.0
7907 9674 12243 15441 24710 26982 33640 43133 44595 50287 54827 64721 67489 74562 81132 87526 92164 93261 100236 104765 111288 113672 118844 127882 130632 139001 143020 145716 151535 153449 157614 165484 178560 182475 185264 190188 191493 202627 206308 212462 214187 220647 226528 231108 234628 240353 246142 247163 256576 259053 264031 267505 270238 274564 276606 285205 291380 296123 301517 304503 307891 313080 322153 326331 329094 335584 341498 344819 353283 354590 357841 364991 370151 382674 385196 389038 394419 403278 416631 419038 422331 430447 434704 445323 452117 456454 458944 461645 464987 466426 475191 478433 481392

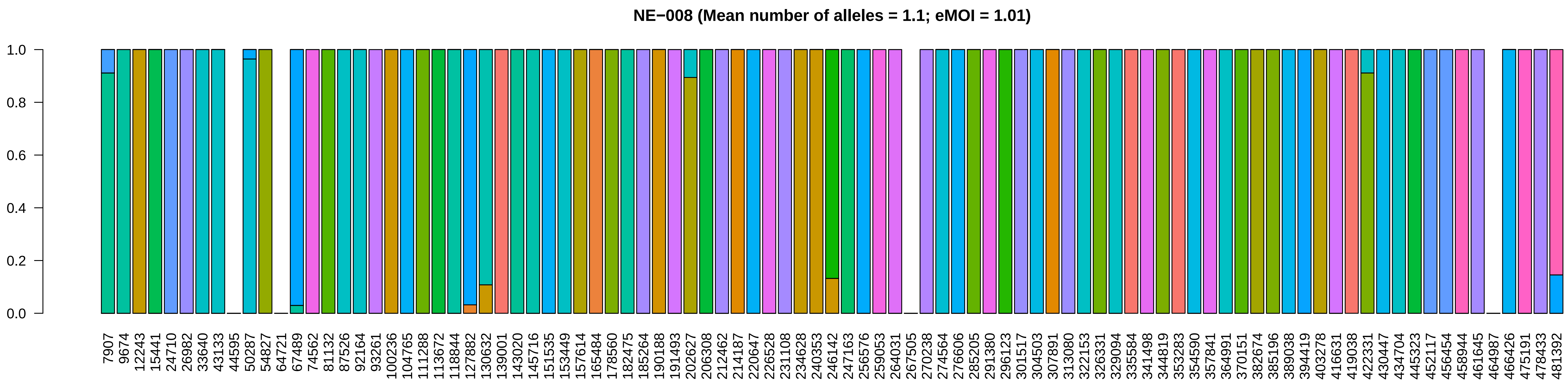
NE−008 (Mean number of alleles = 1.1; eMOI = 1.01)
0.0
0.2
0.4
0.6
0.8
1.0
7907
9674
12243
15441
24710
26982
33640
43133
44595
50287
54827
64721
67489
74562
81132
87526
92164
93261
100236
104765
111288
113672
118844
127882
130632
139001
143020
145716
151535
153449
157614
165484
178560
182475
185264
190188
191493
202627
206308
212462
214187
220647
226528
231108
234628
240353
246142
247163
256576
259053
264031
267505
270238
274564
276606
285205
291380
296123
301517
304503
307891
313080
322153
326331
329094
335584
341498
344819
353283
354590
357841
364991
370151
382674
385196
389038
394419
403278
416631
419038
422331
430447
434704
445323
452117
456454
458944
461645
464987
466426
475191
478433
481392

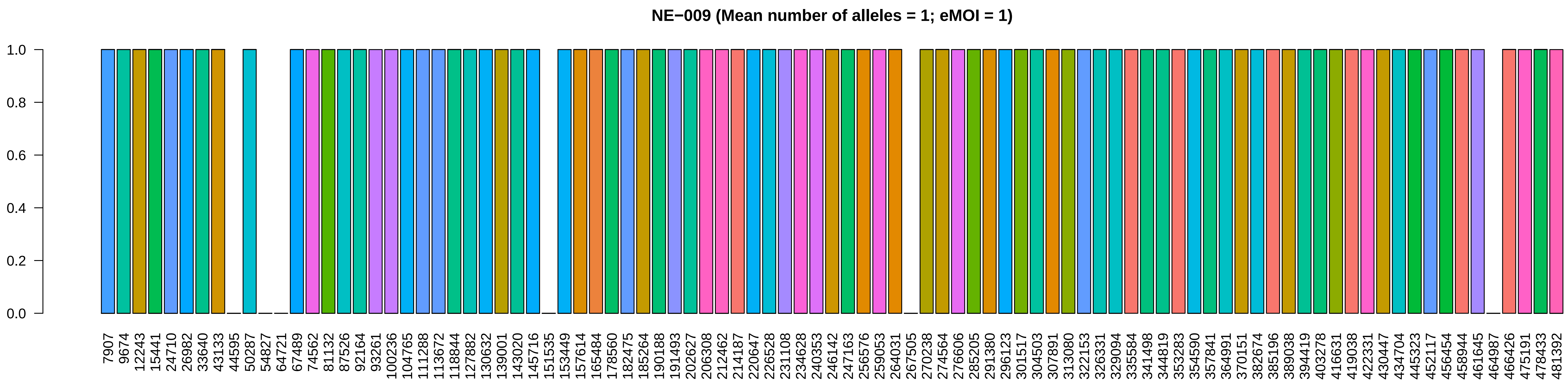
NE−009 (Mean number of alleles = 1; eMOI = 1)
1.0
0.8
0.6
0.4
0.2
0.0
7907 9674 12243 15441 24710 26982 33640 43133 44595 50287 54827 64721 67489 74562 81132 87526 92164 93261 100236 104765 111288 113672 118844 127882 130632 139001 143020 145716 151535 153449 157614 165484 178560 182475 185264 190188 191493 202627 206308 212462 214187 220647 226528 231108 234628 240353 246142 247163 256576 259053 264031 267505 270238 274564 276606 285205 291380 296123 301517 304503 307891 313080 322153 326331 329094 335584 341498 344819 353283 354590 357841 364991 370151 382674 385196 389038 394419 403278 416631 419038 422331 430447 434704 445323 452117 456454 458944 461645 464987 466426 475191 478433 481392

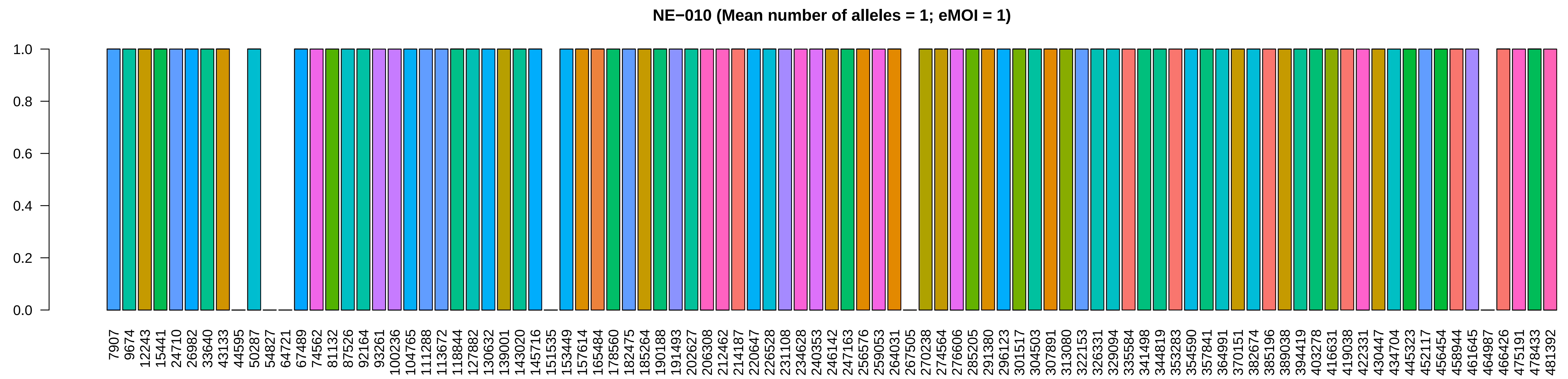

NE−010 (Mean number of alleles = 1; eMOI = 1)
1.0
0.8
0.6
0.4
0.2
0.0
7907 9674 12243 15441 24710 26982 33640 43133 44595 50287 54827 64721 67489 74562 81132 87526 92164 93261 100236 104765 111288 113672 118844 127882 130632 139001 143020 145716 151535 153449 157614 165484 178560 182475 185264 190188 191493 202627 206308 212462 214187 220647 226528 231108 234628 240353 246142 247163 256576 259053 264031 267505 270238 274564 276606 285205 291380 296123 301517 304503 307891 313080 322153 326331 329094 335584 341498 344819 353283 354590 357841 364991 370151 382674 385196 389038 394419 403278 416631 419038 422331 430447 434704 445323 452117 456454 458944 461645 464987 466426 475191 478433 481392

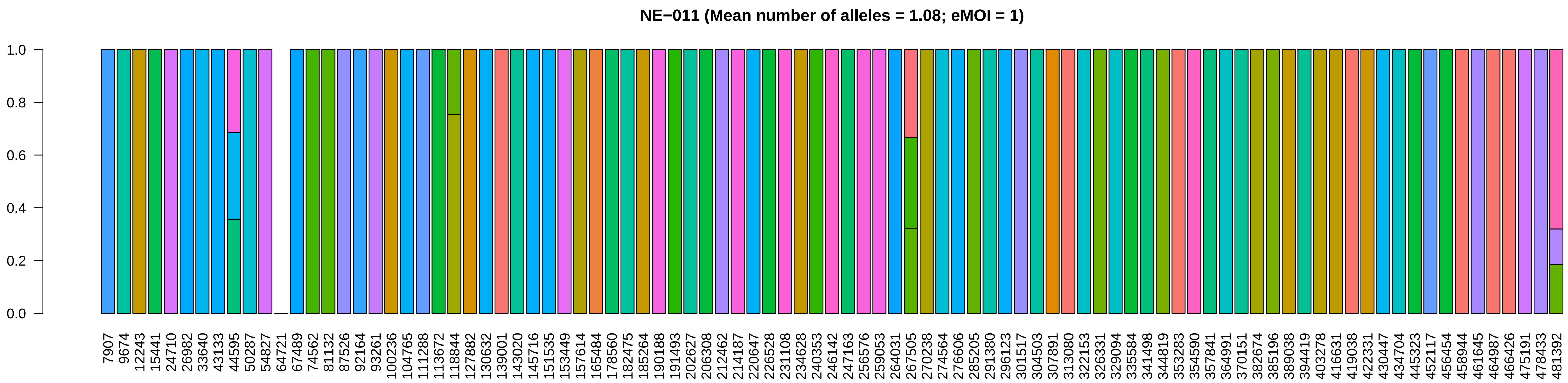
NE−011 (Mean number of alleles = 1.08; eMOI = 1)
1.0
0.8
0.6
0.4
0.2
0.0
7907
9674
12243
15441
24710
26982
33640
43133
44595
50287
54827
64721
67489
74562
81132
87526
92164
93261
100236
104765
111288
113672
118844
127882
130632
139001
143020
145716
151535
153449
157614
165484
178560
182475
185264
190188
191493
202627
206308
212462
214187
220647
226528
231108
234628
240353
246142
247163
256576
259053
264031
267505
270238
274564
276606
285205
291380
296123
301517
304503
307891
313080
322153
326331
329094
335584
341498
344819
353283
354590
357841
364991
370151
382674
385196
389038
394419
403278
416631
419038
422331
430447
434704
445323
452117
456454
458944
461645
464987
466426
475191
478433
481392

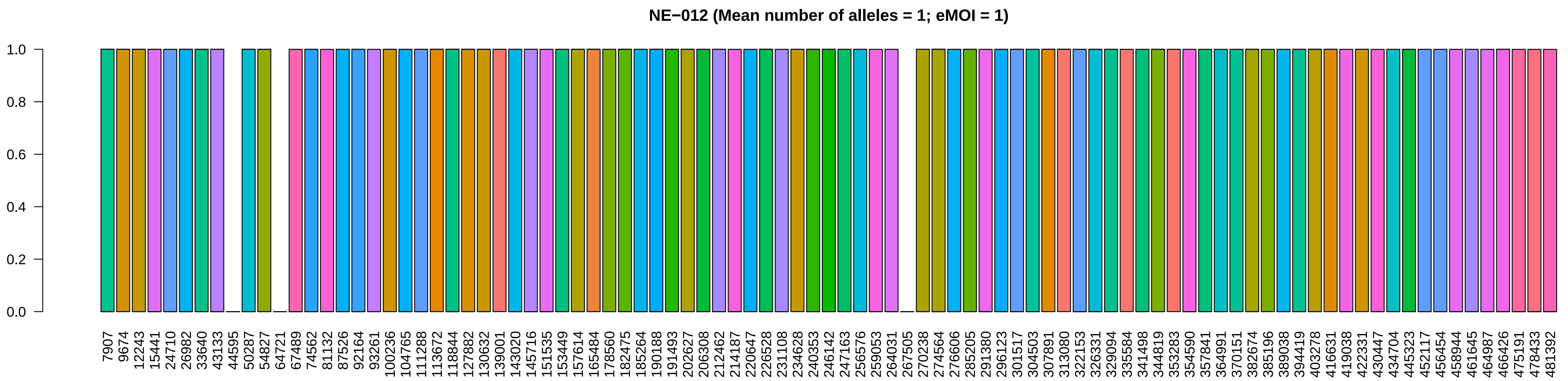
NE−012 (Mean number of alleles = 1; eMOI = 1)
1.0
0.8
0.6
0.4
0.2
0.0
7907 9674 12243 15441 24710 26982 33640 43133 44595 50287 54827 64721 67489 74562 81132 87526 92164 93261 100236 104765 111288 113672 118844 127882 130632 139001 143020 145716 151535 153449 157614 165484 178560 182475 185264 190188 191493 202627 206308 212462 214187 220647 226528 231108 234628 240353 246142 247163 256576 259053 264031 267505 270238 274564 276606 285205 291380 296123 301517 304503 307891 313080 322153 326331 329094 335584 341498 344819 353283 354590 357841 364991 370151 382674 385196 389038 394419 403278 416631 419038 422331 430447 434704 445323 452117 456454 458944 461645 464987 466426 475191 478433 481392

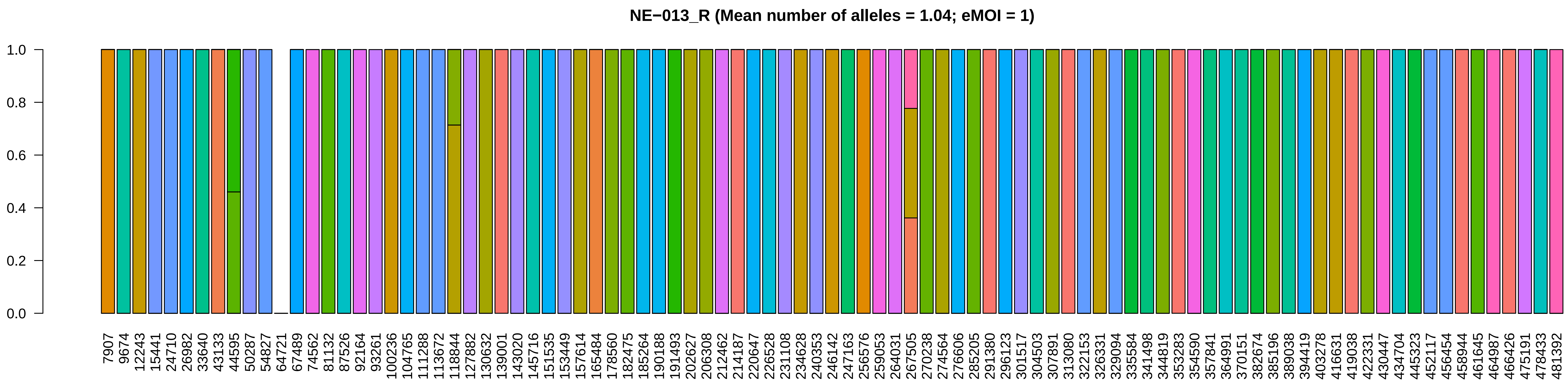

NE−013_R (Mean number of alleles = 1.04; eMOI = 1)
1.0
0.8
0.6
0.4
0.2
0.0
7907
9674
12243
15441
24710
26982
33640
43133
44595
50287
54827
64721
67489
74562
81132
87526
92164
93261
100236
104765
111288
113672
118844
127882
130632
139001
143020
145716
151535
153449
157614
165484
178560
182475
185264
190188
191493
202627
206308
212462
214187
220647
226528
231108
234628
240353
246142
247163
256576
259053
264031
267505
270238
274564
276606
285205
291380
296123
301517
304503
307891
313080
322153
326331
329094
335584
341498
344819
353283
354590
357841
364991
370151
382674
385196
389038
394419
403278
416631
419038
422331
430447
434704
445323
452117
456454
458944
461645
464987
466426
475191
478433
481392

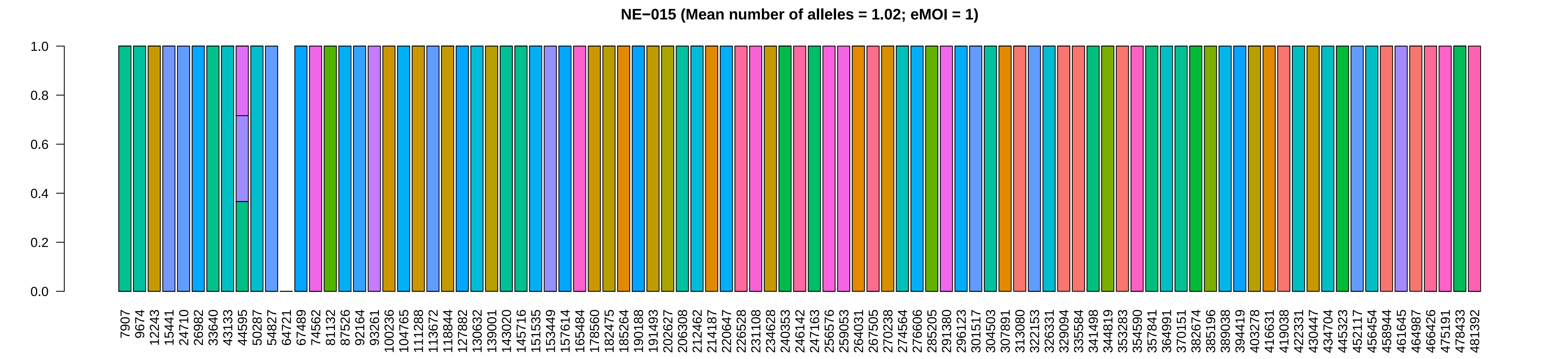
NE−015 (Mean number of alleles = 1.02; eMOI = 1)
1.0
0.8
0.6
0.4
0.2
0.0
7907
9674
12243
15441
24710
26982
33640
43133
44595
50287
54827
64721
67489
74562
81132
87526
92164
93261
100236
104765
111288
113672
118844
127882
130632
139001
143020
145716
151535
153449
157614
165484
178560
182475
185264
190188
191493
202627
206308
212462
214187
220647
226528
231108
234628
240353
246142
247163
256576
259053
264031
267505
270238
274564
276606
285205
291380
296123
301517
304503
307891
313080
322153
326331
329094
335584
341498
344819
353283
354590
357841
364991
370151
382674
385196
389038
394419
403278
416631
419038
422331
430447
434704
445323
452117
456454
458944
461645
464987
466426
475191
478433
481392

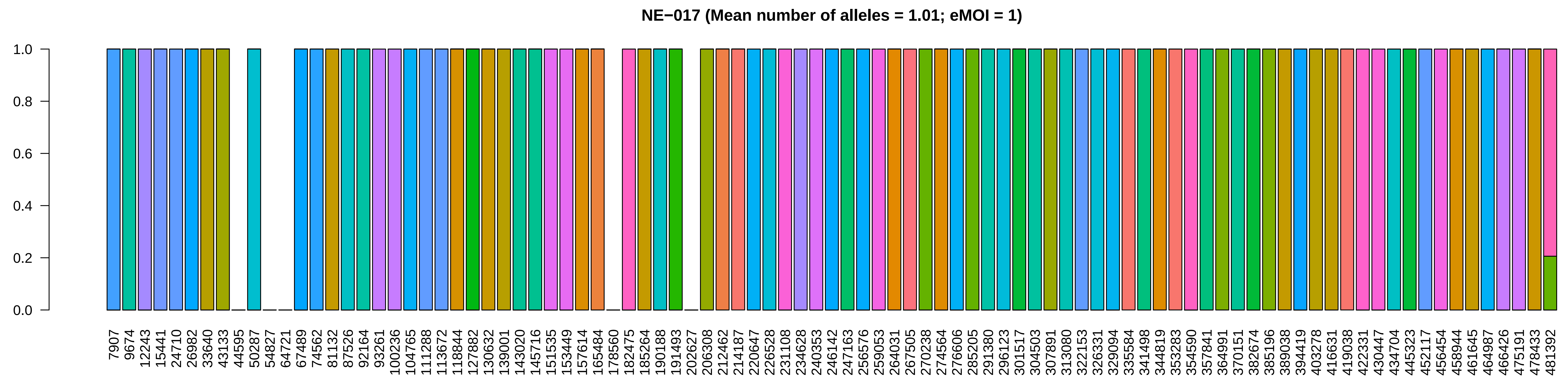
NE−017 (Mean number of alleles = 1.01; eMOI = 1)
1.0
0.8
0.6
0.4
0.2
0.0
7907
9674
12243
15441
24710
26982
33640
43133
44595
50287
54827
64721
67489
74562
81132
87526
92164
93261
100236
104765
111288
113672
118844
127882
130632
139001
143020
145716
151535
153449
157614
165484
178560
182475
185264
190188
191493
202627
206308
212462
214187
220647
226528
231108
234628
240353
246142
247163
256576
259053
264031
267505
270238
274564
276606
285205
291380
296123
301517
304503
307891
313080
322153
326331
329094
335584
341498
344819
353283
354590
357841
364991
370151
382674
385196
389038
394419
403278
416631
419038
422331
430447
434704
445323
452117
456454
458944
461645
464987
466426
475191
478433
481392

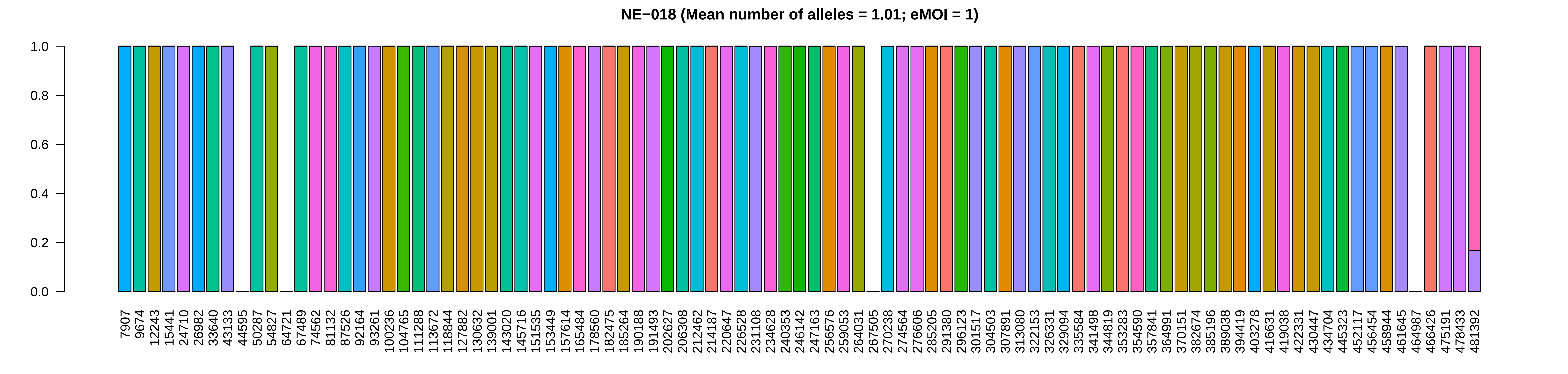

NE−018 (Mean number of alleles = 1.01; eMOI = 1)
1.0
0.8
0.6
0.4
0.2
0.0
7907 9674 12243 15441 24710 26982 33640 43133 44595 50287 54827 64721 67489 74562 81132 87526 92164 93261 100236 104765 111288 113672 118844 127882 130632 139001 143020 145716 151535 153449 157614 165484 178560 182475 185264 190188 191493 202627 206308 212462 214187 220647 226528 231108 234628 240353 246142 247163 256576 259053 264031 267505 270238 274564 276606 285205 291380 296123 301517 304503 307891 313080 322153 326331 329094 335584 341498 344819 353283 354590 357841 364991 370151 382674 385196 389038 394419 403278 416631 419038 422331 430447 434704 445323 452117 456454 458944 461645 464987 466426 475191 478433 481392

NE−019 (Mean number of alleles = 1; eMOI = 1)

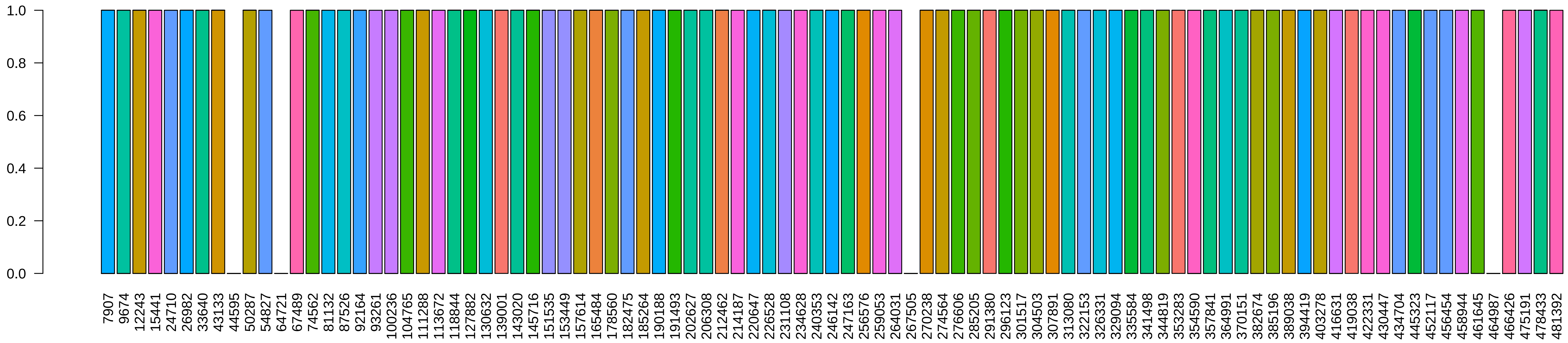

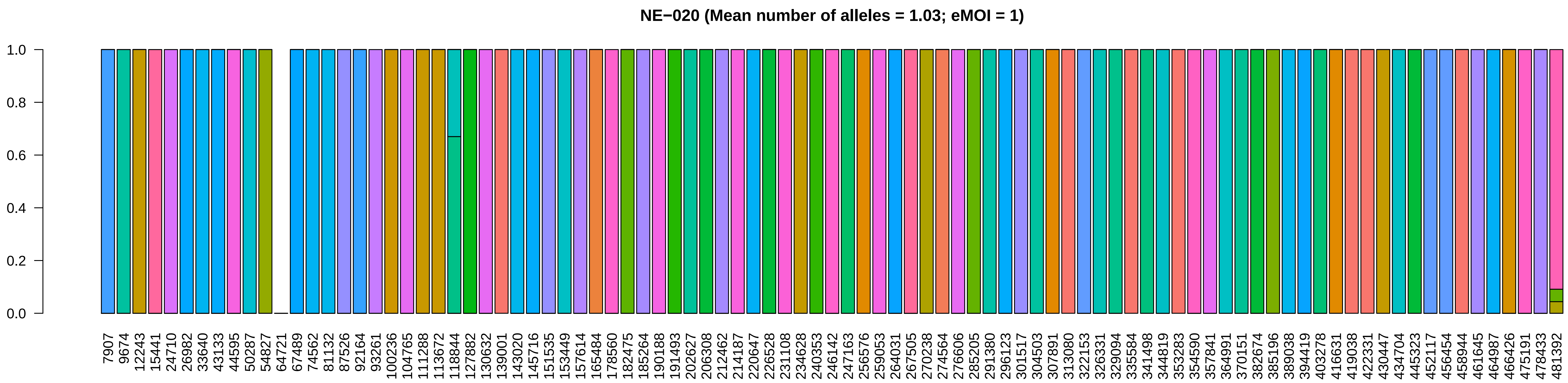
NE−020 (Mean number of alleles = 1.03; eMOI = 1)
1.0
0.8
0.6
0.4
0.2
0.0
7907
9674
12243
15441
24710
26982
33640
43133
44595
50287
54827
64721
67489
74562
81132
87526
92164
93261
100236
104765
111288
113672
118844
127882
130632
139001
143020
145716
151535
153449
157614
165484
178560
182475
185264
190188
191493
202627
206308
212462
214187
220647
226528
231108
234628
240353
246142
247163
256576
259053
264031
267505
270238
274564
276606
285205
291380
296123
301517
304503
307891
313080
322153
326331
329094
335584
341498
344819
353283
354590
357841
364991
370151
382674
385196
389038
394419
403278
416631
419038
422331
430447
434704
445323
452117
456454
458944
461645
464987
466426
475191
478433
481392

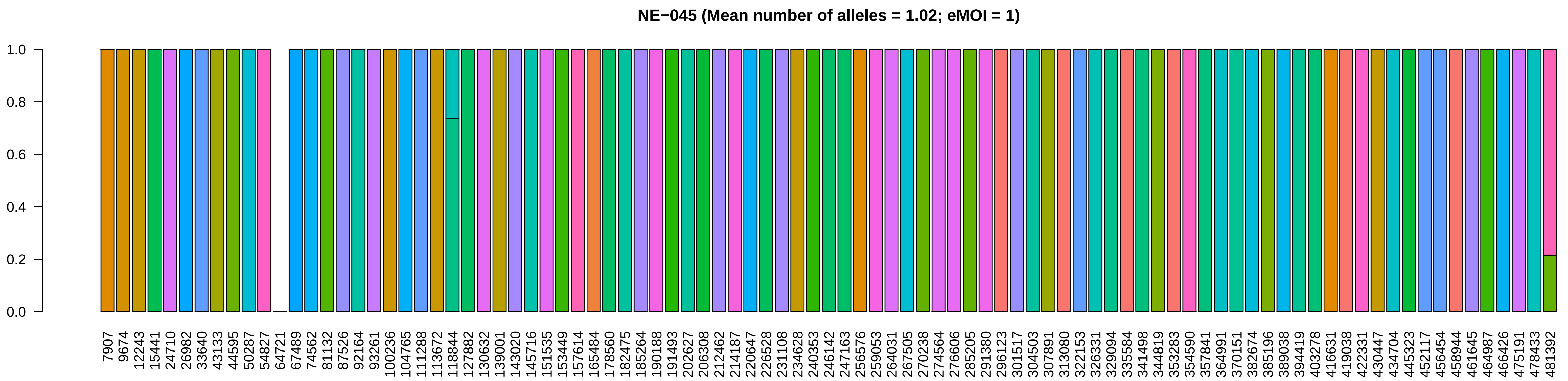
NE−045 (Mean number of alleles = 1.02; eMOI = 1)
1.0
0.8
0.6
0.4
0.2
0.0
7907 9674 12243 15441 24710 26982 33640 43133 44595 50287 54827 64721 67489 74562 81132 87526 92164 93261 100236 104765 111288 113672 118844 127882 130632 139001 143020 145716 151535 153449 157614 165484 178560 182475 185264 190188 191493 202627 206308 212462 214187 220647 226528 231108 234628 240353 246142 247163 256576 259053 264031 267505 270238 274564 276606 285205 291380 296123 301517 304503 307891 313080 322153 326331 329094 335584 341498 344819 353283 354590 357841 364991 370151 382674 385196 389038 394419 403278 416631 419038 422331 430447 434704 445323 452117 456454 458944 461645 464987 466426 475191 478433 481392

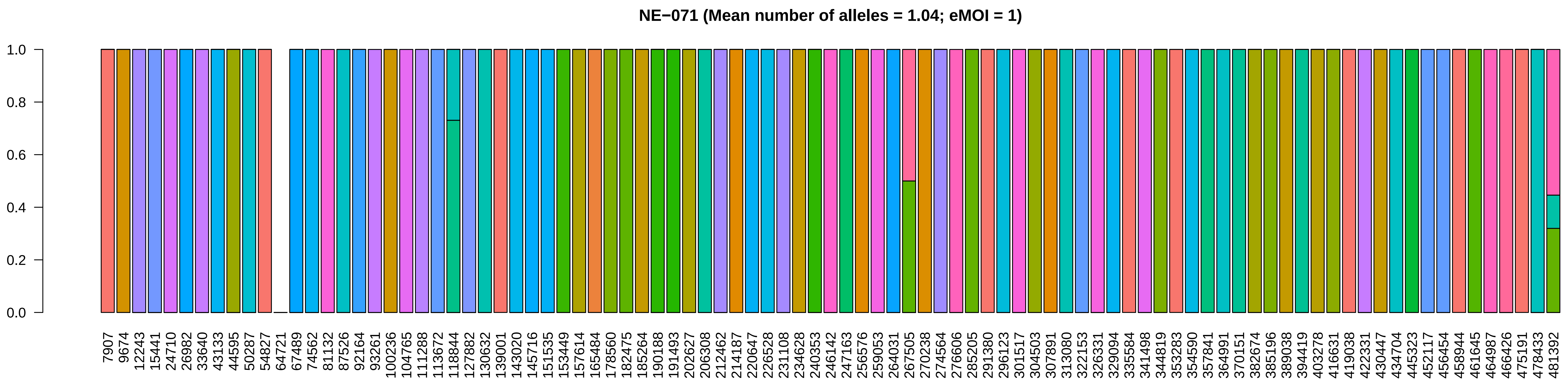
NE−071 (Mean number of alleles = 1.04; eMOI = 1)
1.0
0.8
0.6
0.4
0.2
0.0
7907
9674
12243
15441
24710
26982
33640
43133
44595
50287
54827
64721
67489
74562
81132
87526
92164
93261
100236
104765
111288
113672
118844
127882
130632
139001
143020
145716
151535
153449
157614
165484
178560
182475
185264
190188
191493
202627
206308
212462
214187
220647
226528
231108
234628
240353
246142
247163
256576
259053
264031
267505
270238
274564
276606
285205
291380
296123
301517
304503
307891
313080
322153
326331
329094
335584
341498
344819
353283
354590
357841
364991
370151
382674
385196
389038
394419
403278
416631
419038
422331
430447
434704
445323
452117
456454
458944
461645
464987
466426
475191
478433
481392

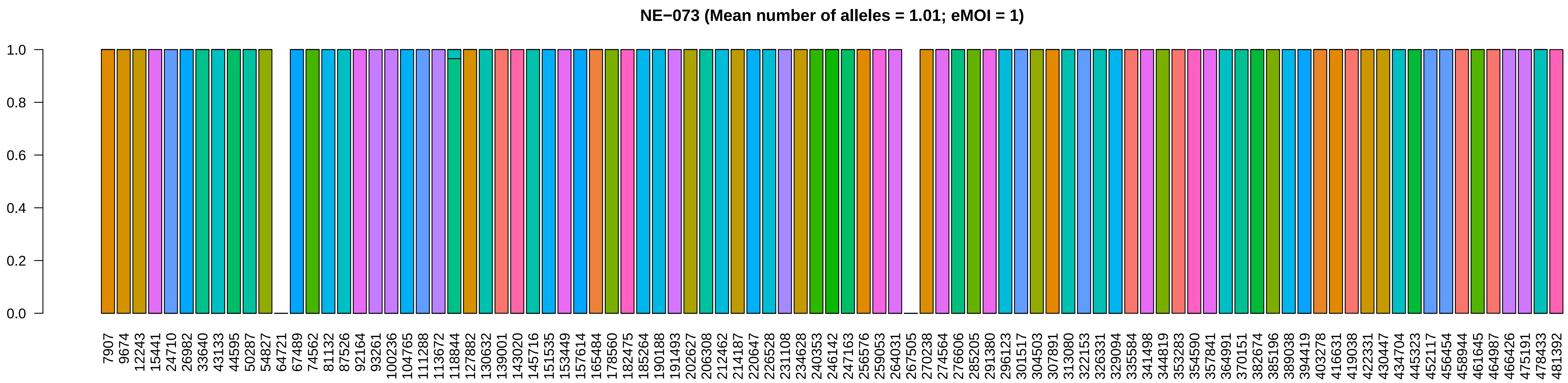
NE−073 (Mean number of alleles = 1.01; eMOI = 1)
1.0
0.8
0.6
0.4
0.2
0.0
7907
9674
12243
15441
24710
26982
33640
43133
44595
50287
54827
64721
67489
74562
81132
87526
92164
93261
100236
104765
111288
113672
118844
127882
130632
139001
143020
145716
151535
153449
157614
165484
178560
182475
185264
190188
191493
202627
206308
212462
214187
220647
226528
231108
234628
240353
246142
247163
256576
259053
264031
267505
270238
274564
276606
285205
291380
296123
301517
304503
307891
313080
322153
326331
329094
335584
341498
344819
353283
354590
357841
364991
370151
382674
385196
389038
394419
403278
416631
419038
422331
430447
434704
445323
452117
456454
458944
461645
464987
466426
475191
478433
481392

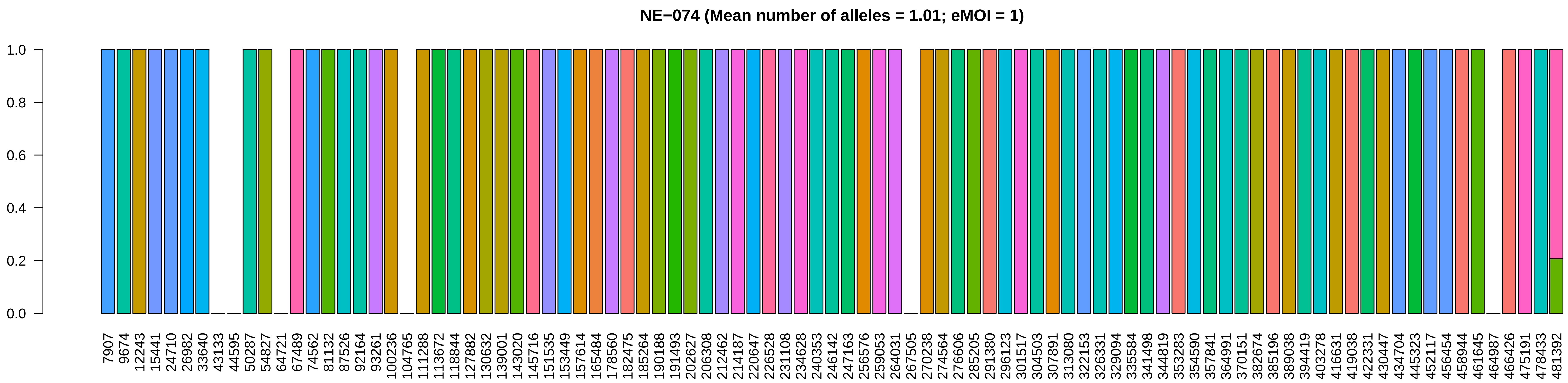
NE−074 (Mean number of alleles = 1.01; eMOI = 1)
1.0
0.8
0.6
0.4
0.2
0.0
7907
9674
12243
15441
24710
26982
33640
43133
44595
50287
54827
64721
67489
74562
81132
87526
92164
93261
100236
104765
111288
113672
118844
127882
130632
139001
143020
145716
151535
153449
157614
165484
178560
182475
185264
190188
191493
202627
206308
212462
214187
220647
226528
231108
234628
240353
246142
247163
256576
259053
264031
267505
270238
274564
276606
285205
291380
296123
301517
304503
307891
313080
322153
326331
329094
335584
341498
344819
353283
354590
357841
364991
370151
382674
385196
389038
394419
403278
416631
419038
422331
430447
434704
445323
452117
456454
458944
461645
464987
466426
475191
478433
481392

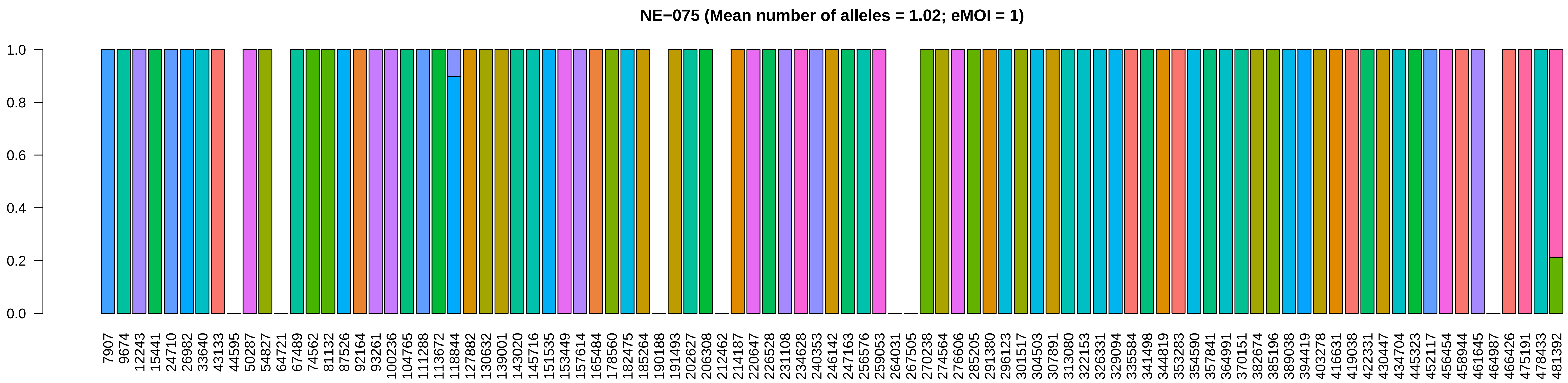

NE−075 (Mean number of alleles = 1.02; eMOI = 1)
0.0
0.2
0.4
0.6
0.8
1.0
7907 9674 12243 15441 24710 26982 33640 43133 44595 50287 54827 64721 67489 74562 81132 87526 92164 93261 100236 104765 111288 113672 118844 127882 130632 139001 143020 145716 151535 153449 157614 165484 178560 182475 185264 190188 191493 202627 206308 212462 214187 220647 226528 231108 234628 240353 246142 247163 256576 259053 264031 267505 270238 274564 276606 285205 291380 296123 301517 304503 307891 313080 322153 326331 329094 335584 341498 344819 353283 354590 357841 364991 370151 382674 385196 389038 394419 403278 416631 419038 422331 430447 434704 445323 452117 456454 458944 461645 464987 466426 475191 478433 481392

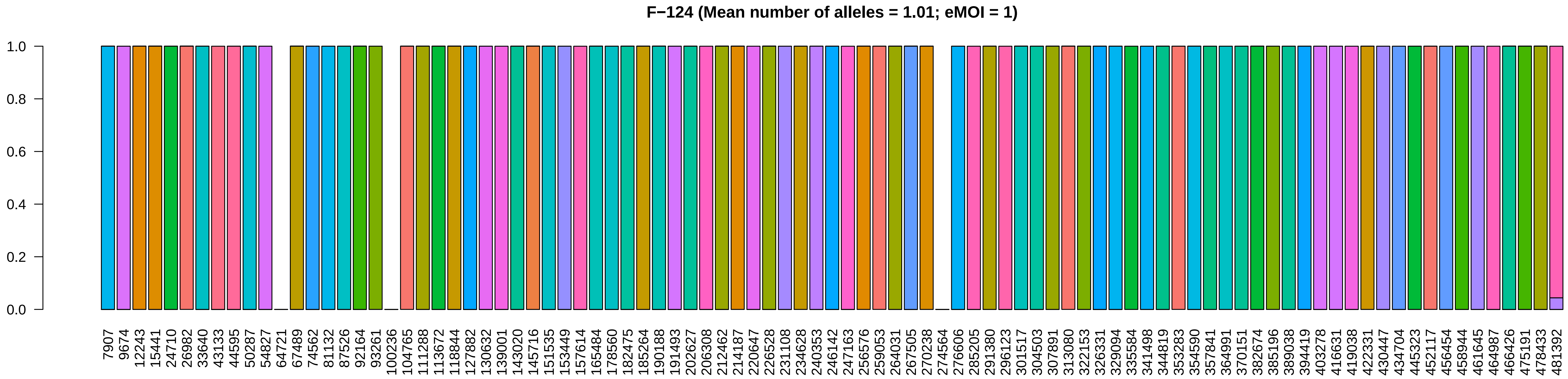
F−124 (Mean number of alleles = 1.01; eMOI = 1)
1.0
0.8
0.6
0.4
0.2
0.0
7907
9674
12243
15441
24710
26982
33640
43133
44595
50287
54827
64721
67489
74562
81132
87526
92164
93261
100236
104765
111288
113672
118844
127882
130632
139001
143020
145716
151535
153449
157614
165484
178560
182475
185264
190188
191493
202627
206308
212462
214187
220647
226528
231108
234628
240353
246142
247163
256576
259053
264031
267505
270238
274564
276606
285205
291380
296123
301517
304503
307891
313080
322153
326331
329094
335584
341498
344819
353283
354590
357841
364991
370151
382674
385196
389038
394419
403278
416631
419038
422331
430447
434704
445323
452117
456454
458944
461645
464987
466426
475191
478433
481392

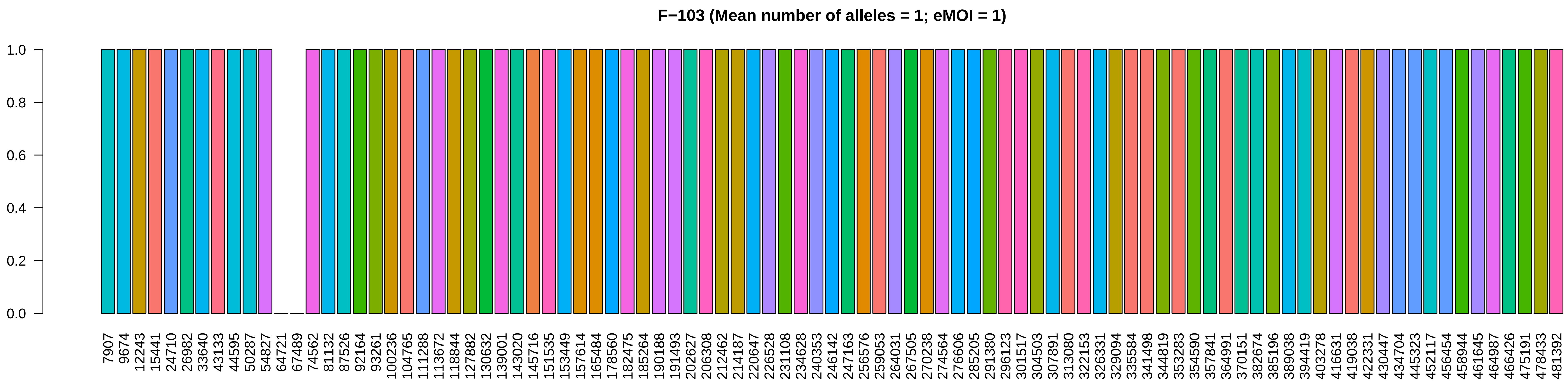
F−103 (Mean number of alleles = 1; eMOI = 1)
1.0
0.8
0.6
0.4
0.2
0.0
7907 9674 12243 15441 24710 26982 33640 43133 44595 50287 54827 64721 67489 74562 81132 87526 92164 93261 100236 104765 111288 113672 118844 127882 130632 139001 143020 145716 151535 153449 157614 165484 178560 182475 185264 190188 191493 202627 206308 212462 214187 220647 226528 231108 234628 240353 246142 247163 256576 259053 264031 267505 270238 274564 276606 285205 291380 296123 301517 304503 307891 313080 322153 326331 329094 335584 341498 344819 353283 354590 357841 364991 370151 382674 385196 389038 394419 403278 416631 419038 422331 430447 434704 445323 452117 456454 458944 461645 464987 466426 475191 478433 481392

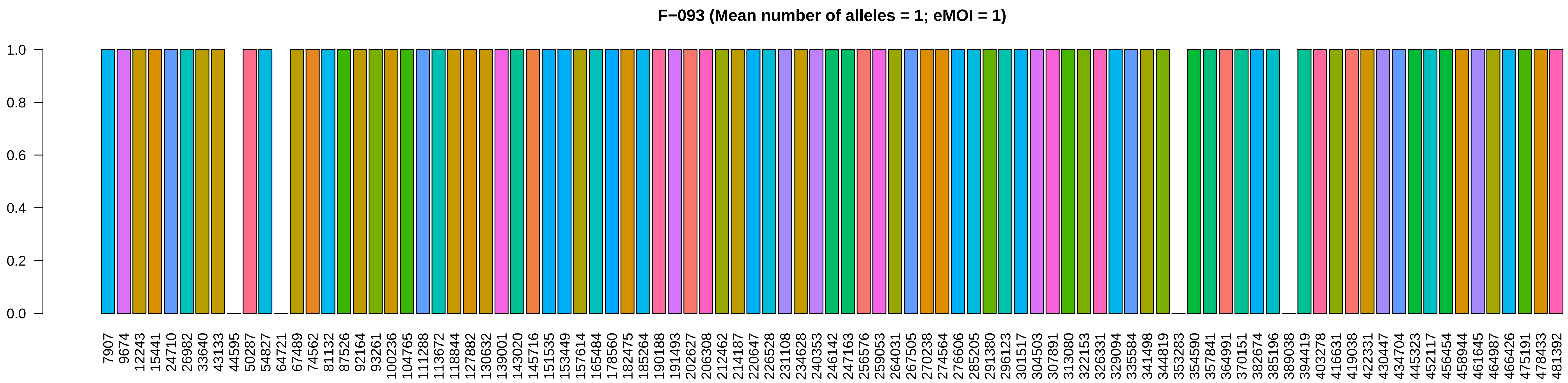
F−093 (Mean number of alleles = 1; eMOI = 1)
1.0
0.8
0.6
0.4
0.2
0.0
7907 9674 12243 15441 24710 26982 33640 43133 44595 50287 54827 64721 67489 74562 81132 87526 92164 93261 100236 104765 111288 113672 118844 127882 130632 139001 143020 145716 151535 153449 157614 165484 178560 182475 185264 190188 191493 202627 206308 212462 214187 220647 226528 231108 234628 240353 246142 247163 256576 259053 264031 267505 270238 274564 276606 285205 291380 296123 301517 304503 307891 313080 322153 326331 329094 335584 341498 344819 353283 354590 357841 364991 370151 382674 385196 389038 394419 403278 416631 419038 422331 430447 434704 445323 452117 456454 458944 461645 464987 466426 475191 478433 481392

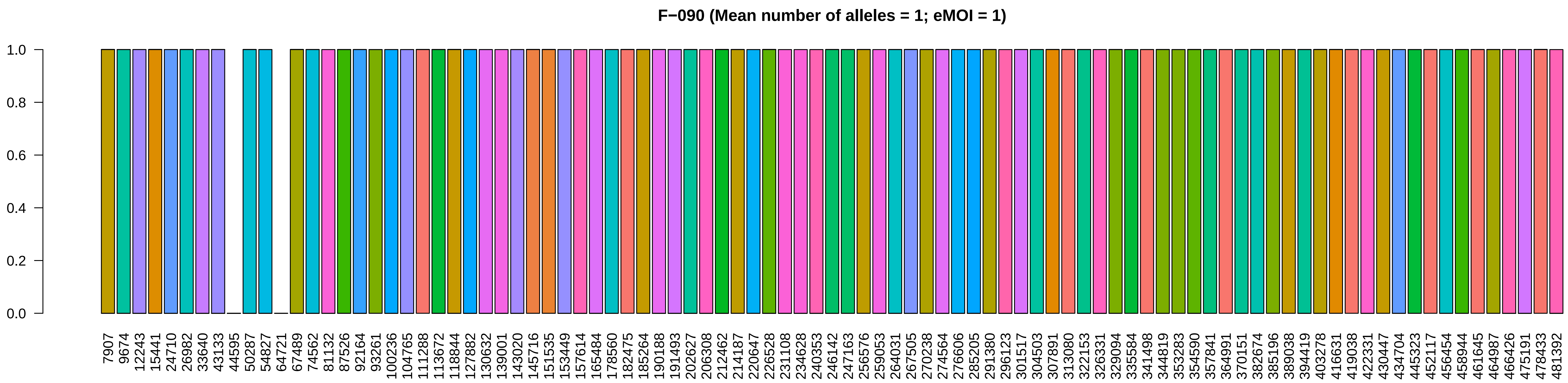
F−090 (Mean number of alleles = 1; eMOI = 1)
1.0
0.8
0.6
0.4
0.2
0.0
7907
9674
12243
15441
24710
26982
33640
43133
44595
50287
54827
64721
67489
74562
81132
87526
92164
93261
100236
104765
111288
113672
118844
127882
130632
139001
143020
145716
151535
153449
157614
165484
178560
182475
185264
190188
191493
202627
206308
212462
214187
220647
226528
231108
234628
240353
246142
247163
256576
259053
264031
267505
270238
274564
276606
285205
291380
296123
301517
304503
307891
313080
322153
326331
329094
335584
341498
344819
353283
354590
357841
364991
370151
382674
385196
389038
394419
403278
416631
419038
422331
430447
434704
445323
452117
456454
458944
461645
464987
466426
475191
478433
481392

F−082 (Mean number of alleles = 1.27; eMOI = 1)

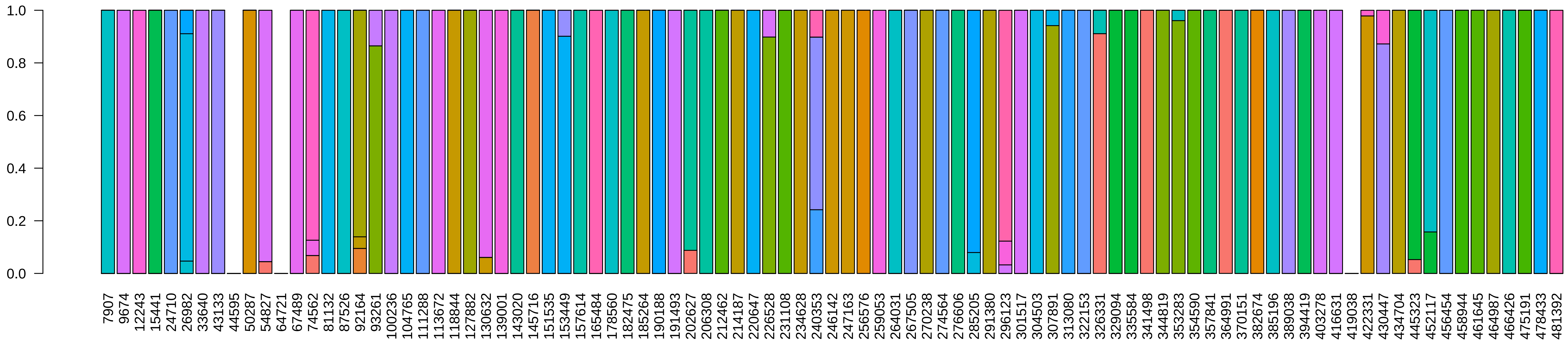

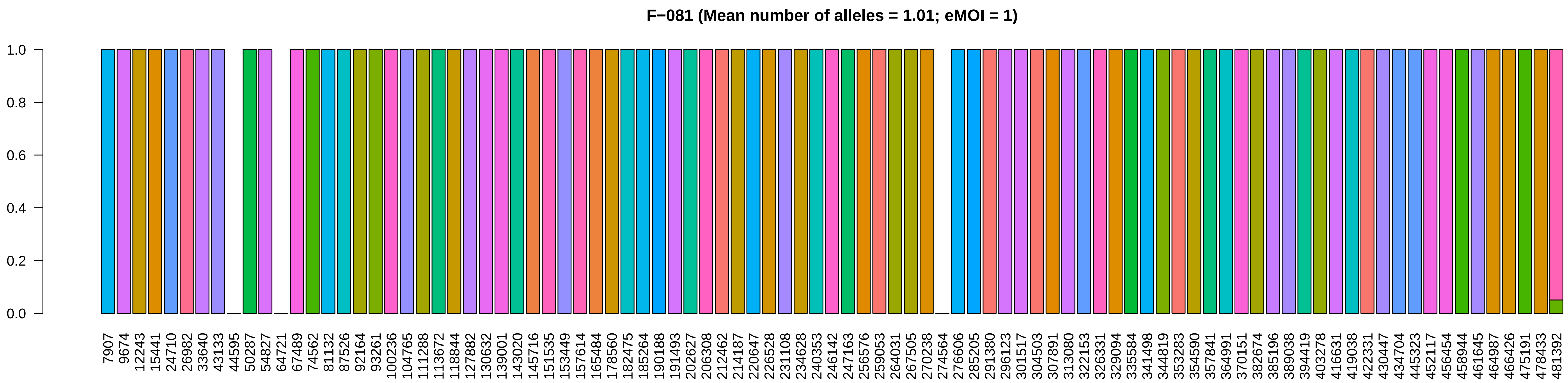
F−081 (Mean number of alleles = 1.01; eMOI = 1)
1.0
0.8
0.6
0.4
0.2
0.0
7907
9674
12243
15441
24710
26982
33640
43133
44595
50287
54827
64721
67489
74562
81132
87526
92164
93261
100236
104765
111288
113672
118844
127882
130632
139001
143020
145716
151535
153449
157614
165484
178560
182475
185264
190188
191493
202627
206308
212462
214187
220647
226528
231108
234628
240353
246142
247163
256576
259053
264031
267505
270238
274564
276606
285205
291380
296123
301517
304503
307891
313080
322153
326331
329094
335584
341498
344819
353283
354590
357841
364991
370151
382674
385196
389038
394419
403278
416631
419038
422331
430447
434704
445323
452117
456454
458944
461645
464987
466426
475191
478433
481392

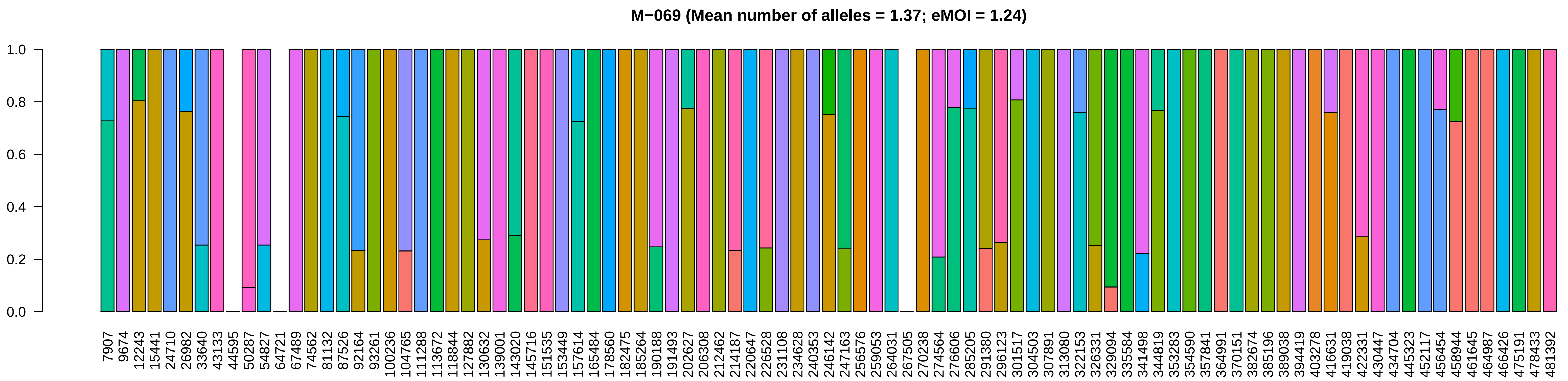

M−069 (Mean number of alleles = 1.37; eMOI = 1.24)
1.0
0.8
0.6
0.4
0.2
0.0
7907 9674 12243 15441 24710 26982 33640 43133 44595 50287 54827 64721 67489 74562 81132 87526 92164 93261 100236 104765 111288 113672 118844 127882 130632 139001 143020 145716 151535 153449 157614 165484 178560 182475 185264 190188 191493 202627 206308 212462 214187 220647 226528 231108 234628 240353 246142 247163 256576 259053 264031 267505 270238 274564 276606 285205 291380 296123 301517 304503 307891 313080 322153 326331 329094 335584 341498 344819 353283 354590 357841 364991 370151 382674 385196 389038 394419 403278 416631 419038 422331 430447 434704 445323 452117 456454 458944 461645 464987 466426 475191 478433 481392

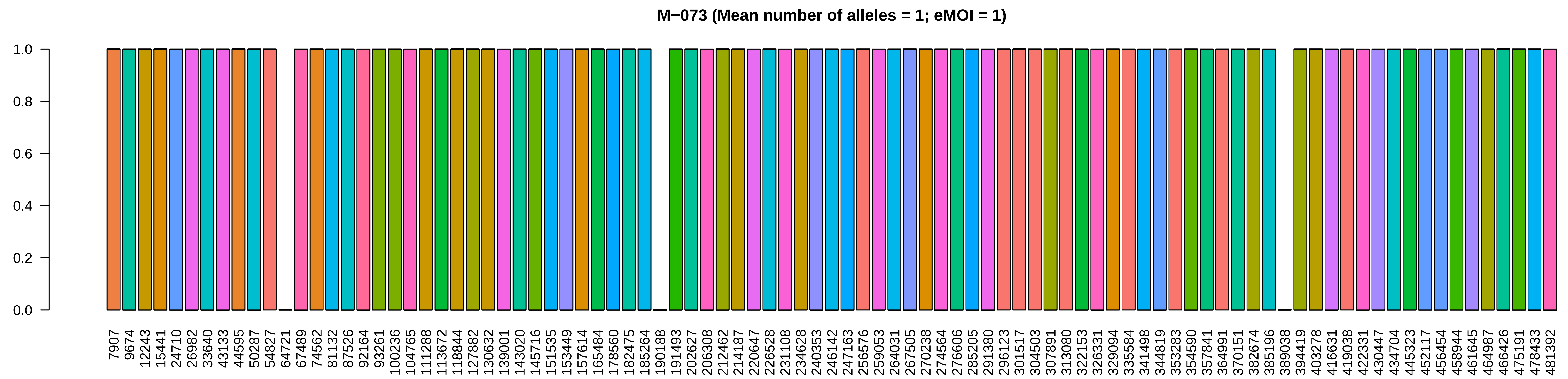
M−073 (Mean number of alleles = 1; eMOI = 1)
1.0
0.8
0.6
0.4
0.2
0.0
7907
9674
12243
15441
24710
26982
33640
43133
44595
50287
54827
64721
67489
74562
81132
87526
92164
93261
100236
104765
111288
113672
118844
127882
130632
139001
143020
145716
151535
153449
157614
165484
178560
182475
185264
190188
191493
202627
206308
212462
214187
220647
226528
231108
234628
240353
246142
247163
256576
259053
264031
267505
270238
274564
276606
285205
291380
296123
301517
304503
307891
313080
322153
326331
329094
335584
341498
344819
353283
354590
357841
364991
370151
382674
385196
389038
394419
403278
416631
419038
422331
430447
434704
445323
452117
456454
458944
461645
464987
466426
475191
478433
481392

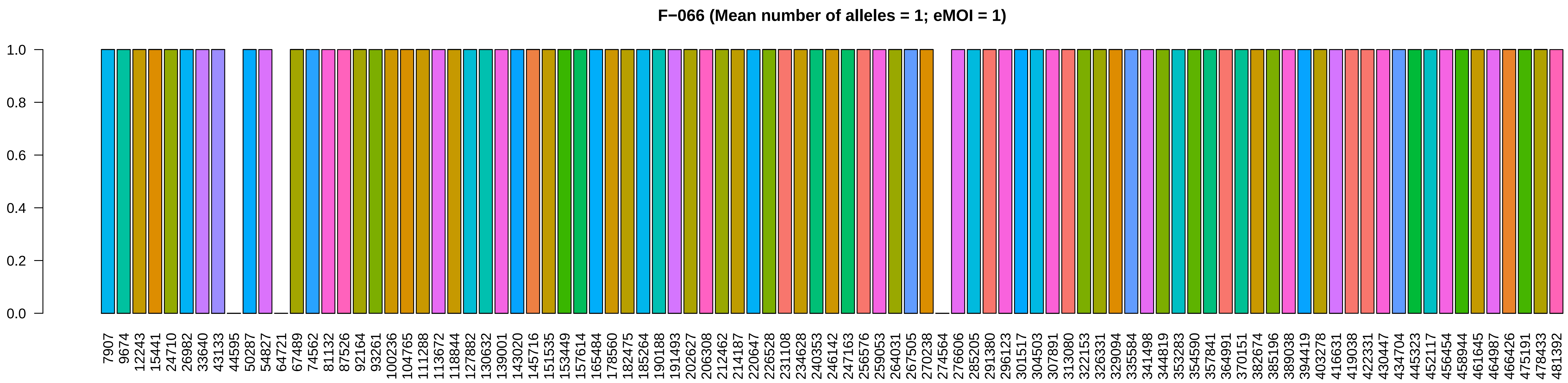

F−066 (Mean number of alleles = 1; eMOI = 1)
0.0
0.2
0.4
0.6
0.8
1.0
7907
9674
12243
15441
24710
26982
33640
43133
44595
50287
54827
64721
67489
74562
81132
87526
92164
93261
100236
104765
111288
113672
118844
127882
130632
139001
143020
145716
151535
153449
157614
165484
178560
182475
185264
190188
191493
202627
206308
212462
214187
220647
226528
231108
234628
240353
246142
247163
256576
259053
264031
267505
270238
274564
276606
285205
291380
296123
301517
304503
307891
313080
322153
326331
329094
335584
341498
344819
353283
354590
357841
364991
370151
382674
385196
389038
394419
403278
416631
419038
422331
430447
434704
445323
452117
456454
458944
461645
464987
466426
475191
478433
481392

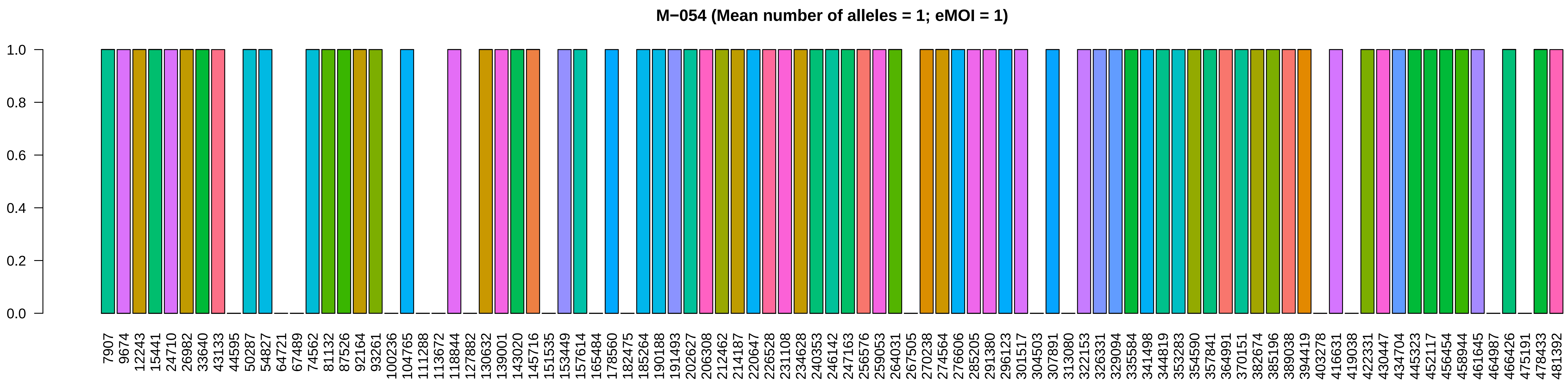

M−054 (Mean number of alleles = 1; eMOI = 1)
1.0
0.8
0.6
0.4
0.2
0.0
7907
9674
12243
15441
24710
26982
33640
43133
44595
50287
54827
64721
67489
74562
81132
87526
92164
93261
100236
104765
111288
113672
118844
127882
130632
139001
143020
145716
151535
153449
157614
165484
178560
182475
185264
190188
191493
202627
206308
212462
214187
220647
226528
231108
234628
240353
246142
247163
256576
259053
264031
267505
270238
274564
276606
285205
291380
296123
301517
304503
307891
313080
322153
326331
329094
335584
341498
344819
353283
354590
357841
364991
370151
382674
385196
389038
394419
403278
416631
419038
422331
430447
434704
445323
452117
456454
458944
461645
464987
466426
475191
478433
481392

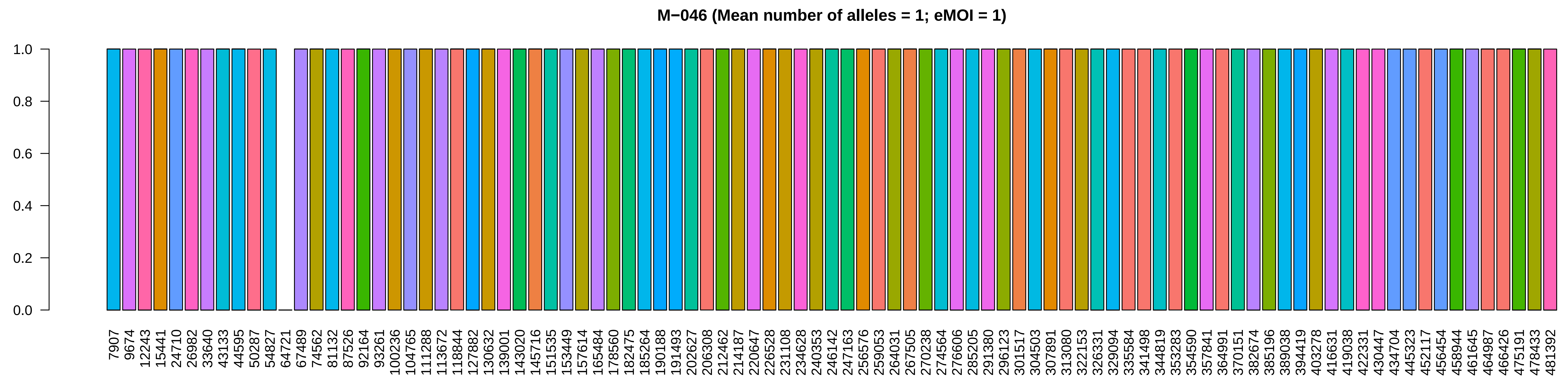
M−046 (Mean number of alleles = 1; eMOI = 1)
1.0
0.8
0.6
0.4
0.2
0.0
7907 9674 12243 15441 24710 26982 33640 43133 44595 50287 54827 64721 67489 74562 81132 87526 92164 93261 100236 104765 111288 113672 118844 127882 130632 139001 143020 145716 151535 153449 157614 165484 178560 182475 185264 190188 191493 202627 206308 212462 214187 220647 226528 231108 234628 240353 246142 247163 256576 259053 264031 267505 270238 274564 276606 285205 291380 296123 301517 304503 307891 313080 322153 326331 329094 335584 341498 344819 353283 354590 357841 364991 370151 382674 385196 389038 394419 403278 416631 419038 422331 430447 434704 445323 452117 456454 458944 461645 464987 466426 475191 478433 481392

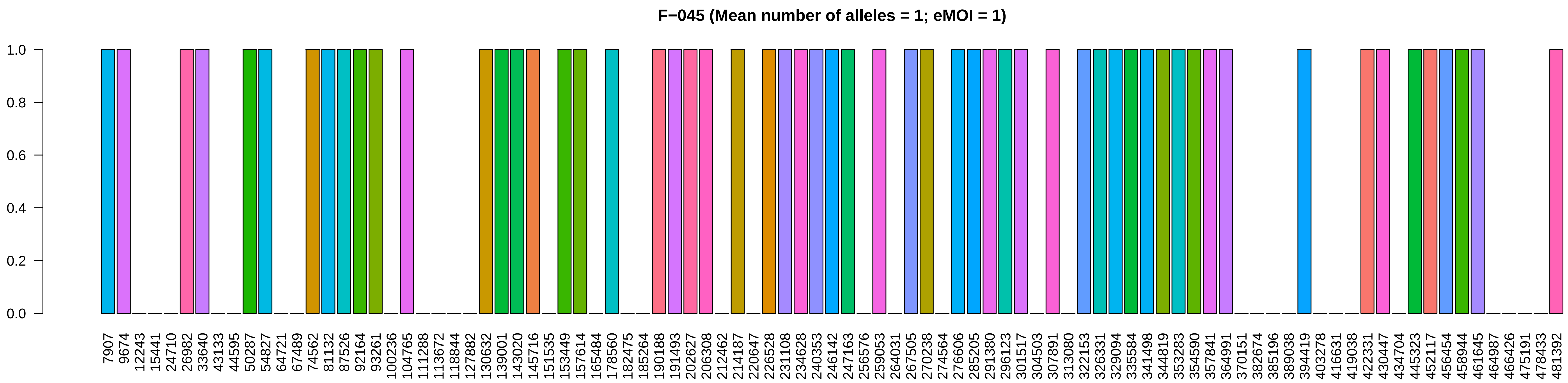
F−045 (Mean number of alleles = 1; eMOI = 1)
0.0
0.2
0.4
0.6
0.8
1.0
7907
9674
12243
15441
24710
26982
33640
43133
44595
50287
54827
64721
67489
74562
81132
87526
92164
93261
100236
104765
111288
113672
118844
127882
130632
139001
143020
145716
151535
153449
157614
165484
178560
182475
185264
190188
191493
202627
206308
212462
214187
220647
226528
231108
234628
240353
246142
247163
256576
259053
264031
267505
270238
274564
276606
285205
291380
296123
301517
304503
307891
313080
322153
326331
329094
335584
341498
344819
353283
354590
357841
364991
370151
382674
385196
389038
394419
403278
416631
419038
422331
430447
434704
445323
452117
456454
458944
461645
464987
466426
475191
478433
481392

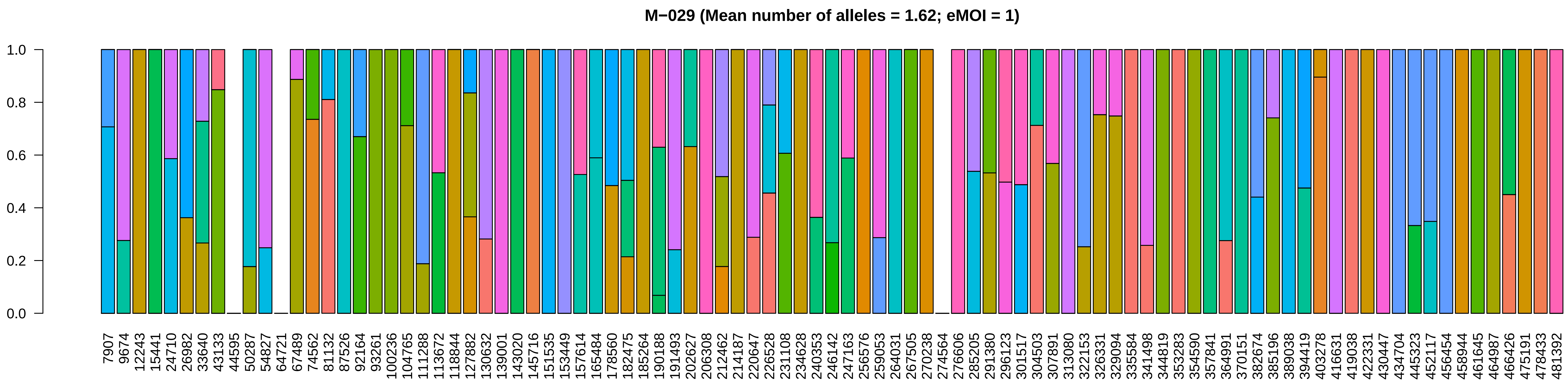
M−029 (Mean number of alleles = 1.62; eMOI = 1)
1.0
0.8
0.6
0.4
0.2
0.0
7907
9674
12243
15441
24710
26982
33640
43133
44595
50287
54827
64721
67489
74562
81132
87526
92164
93261
100236
104765
111288
113672
118844
127882
130632
139001
143020
145716
151535
153449
157614
165484
178560
182475
185264
190188
191493
202627
206308
212462
214187
220647
226528
231108
234628
240353
246142
247163
256576
259053
264031
267505
270238
274564
276606
285205
291380
296123
301517
304503
307891
313080
322153
326331
329094
335584
341498
344819
353283
354590
357841
364991
370151
382674
385196
389038
394419
403278
416631
419038
422331
430447
434704
445323
452117
456454
458944
461645
464987
466426
475191
478433
481392

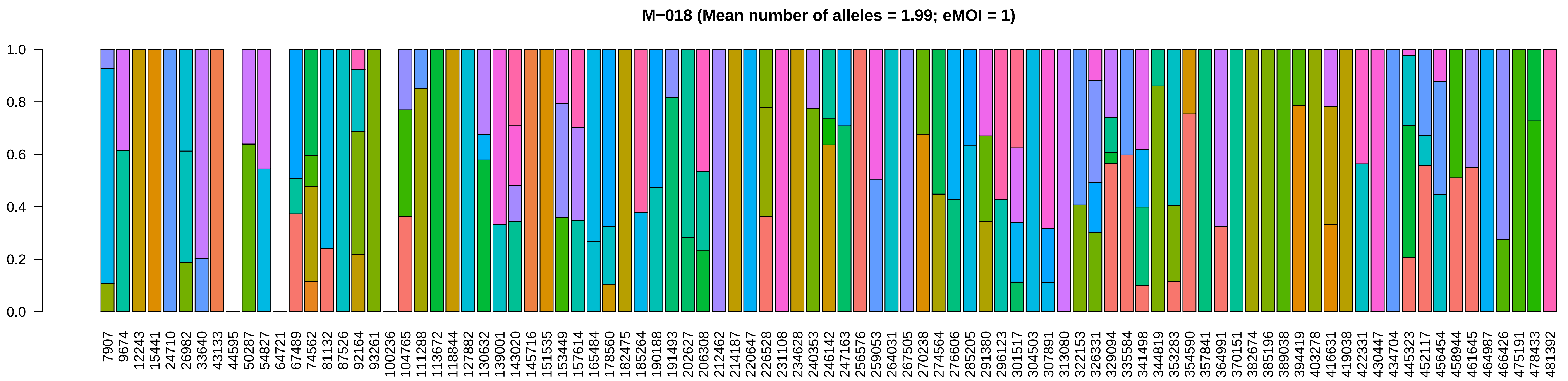
M−018 (Mean number of alleles = 1.99; eMOI = 1)
1.0
0.8
0.6
0.4
0.2
0.0
7907
9674
12243
15441
24710
26982
33640
43133
44595
50287
54827
64721
67489
74562
81132
87526
92164
93261
100236
104765
111288
113672
118844
127882
130632
139001
143020
145716
151535
153449
157614
165484
178560
182475
185264
190188
191493
202627
206308
212462
214187
220647
226528
231108
234628
240353
246142
247163
256576
259053
264031
267505
270238
274564
276606
285205
291380
296123
301517
304503
307891
313080
322153
326331
329094
335584
341498
344819
353283
354590
357841
364991
370151
382674
385196
389038
394419
403278
416631
419038
422331
430447
434704
445323
452117
456454
458944
461645
464987
466426
475191
478433
481392

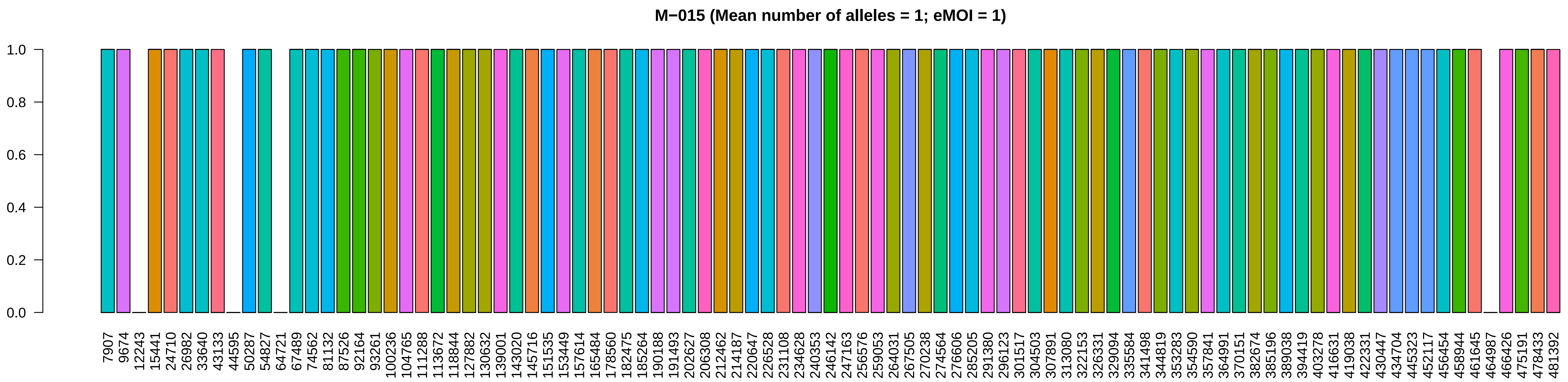
M−015 (Mean number of alleles = 1; eMOI = 1)
1.0
0.8
0.6
0.4
0.2
0.0
7907
9674
12243
15441
24710
26982
33640
43133
44595
50287
54827
64721
67489
74562
81132
87526
92164
93261
100236
104765
111288
113672
118844
127882
130632
139001
143020
145716
151535
153449
157614
165484
178560
182475
185264
190188
191493
202627
206308
212462
214187
220647
226528
231108
234628
240353
246142
247163
256576
259053
264031
267505
270238
274564
276606
285205
291380
296123
301517
304503
307891
313080
322153
326331
329094
335584
341498
344819
353283
354590
357841
364991
370151
382674
385196
389038
394419
403278
416631
419038
422331
430447
434704
445323
452117
456454
458944
461645
464987
466426
475191
478433
481392

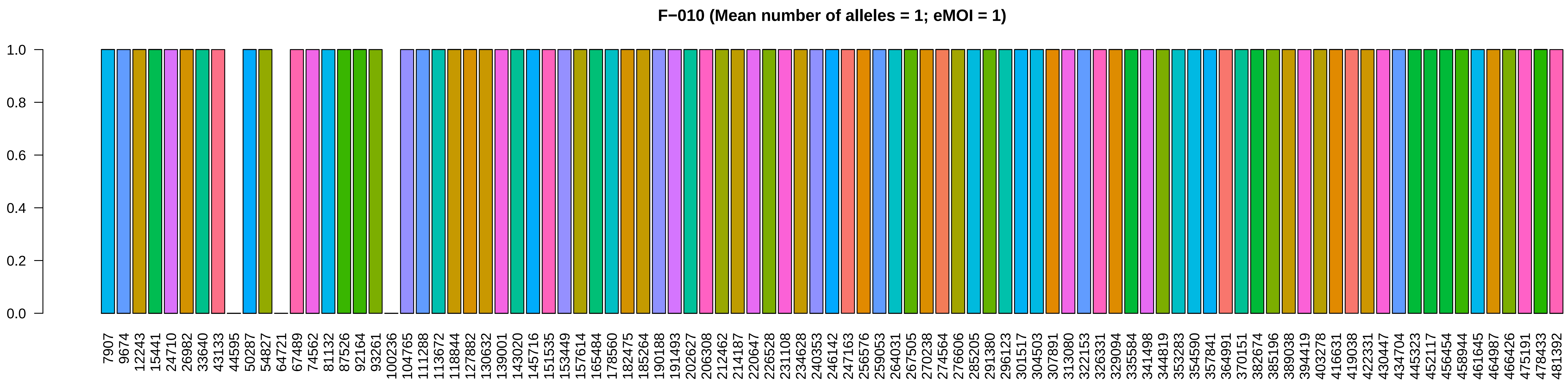

F−010 (Mean number of alleles = 1; eMOI = 1)
1.0
0.8
0.6
0.4
0.2
0.0
7907 9674 12243 15441 24710 26982 33640 43133 44595 50287 54827 64721 67489 74562 81132 87526 92164 93261 100236 104765 111288 113672 118844 127882 130632 139001 143020 145716 151535 153449 157614 165484 178560 182475 185264 190188 191493 202627 206308 212462 214187 220647 226528 231108 234628 240353 246142 247163 256576 259053 264031 267505 270238 274564 276606 285205 291380 296123 301517 304503 307891 313080 322153 326331 329094 335584 341498 344819 353283 354590 357841 364991 370151 382674 385196 389038 394419 403278 416631 419038 422331 430447 434704 445323 452117 456454 458944 461645 464987 466426 475191 478433 481392

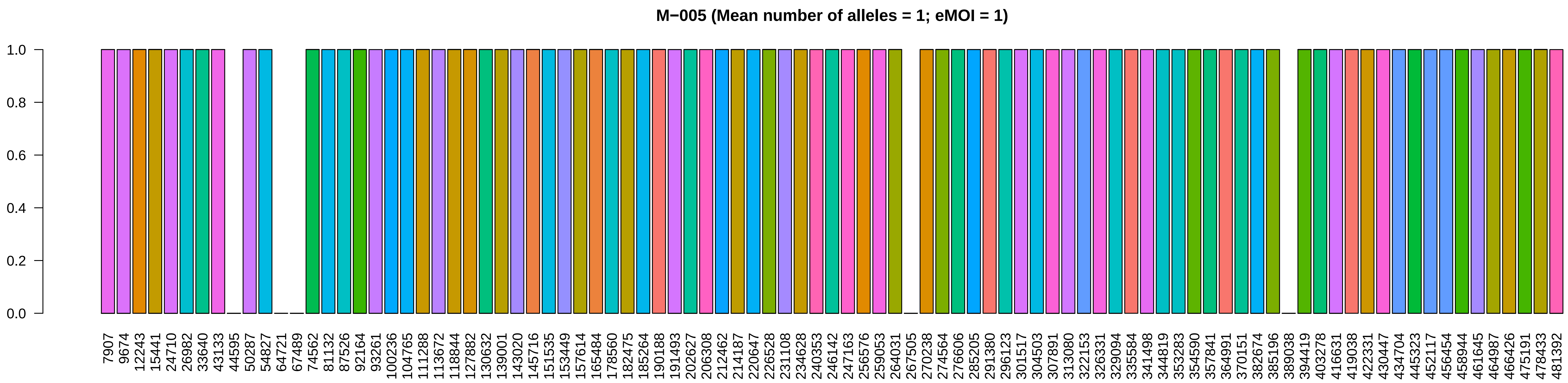
M−005 (Mean number of alleles = 1; eMOI = 1)
1.0
0.8
0.6
0.4
0.2
0.0
7907
9674
12243
15441
24710
26982
33640
43133
44595
50287
54827
64721
67489
74562
81132
87526
92164
93261
100236
104765
111288
113672
118844
127882
130632
139001
143020
145716
151535
153449
157614
165484
178560
182475
185264
190188
191493
202627
206308
212462
214187
220647
226528
231108
234628
240353
246142
247163
256576
259053
264031
267505
270238
274564
276606
285205
291380
296123
301517
304503
307891
313080
322153
326331
329094
335584
341498
344819
353283
354590
357841
364991
370151
382674
385196
389038
394419
403278
416631
419038
422331
430447
434704
445323
452117
456454
458944
461645
464987
466426
475191
478433
481392

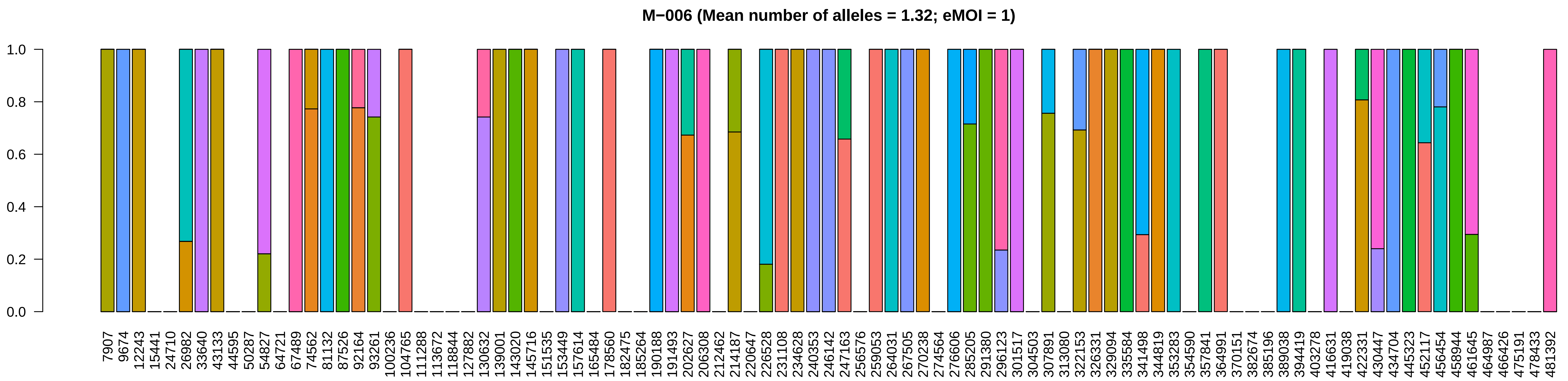
M−006 (Mean number of alleles = 1.32; eMOI = 1)
1.0
0.8
0.6
0.4
0.2
0.0
7907
9674
12243
15441
24710
26982
33640
43133
44595
50287
54827
64721
67489
74562
81132
87526
92164
93261
100236
104765
111288
113672
118844
127882
130632
139001
143020
145716
151535
153449
157614
165484
178560
182475
185264
190188
191493
202627
206308
212462
214187
220647
226528
231108
234628
240353
246142
247163
256576
259053
264031
267505
270238
274564
276606
285205
291380
296123
301517
304503
307891
313080
322153
326331
329094
335584
341498
344819
353283
354590
357841
364991
370151
382674
385196
389038
394419
403278
416631
419038
422331
430447
434704
445323
452117
456454
458944
461645
464987
466426
475191
478433
481392

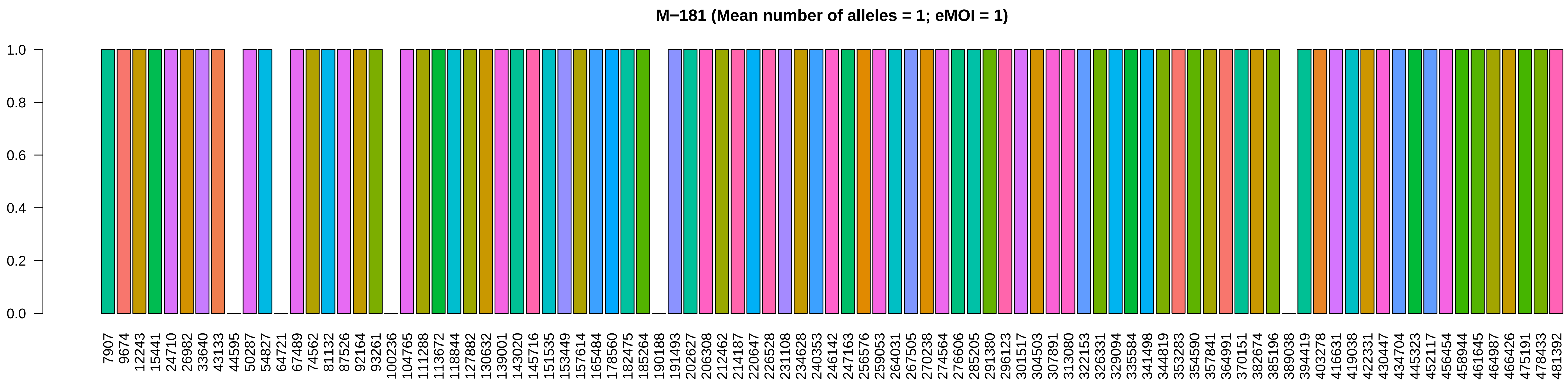
M−181 (Mean number of alleles = 1; eMOI = 1)
1.0
0.8
0.6
0.4
0.2
0.0
7907
9674
12243
15441
24710
26982
33640
43133
44595
50287
54827
64721
67489
74562
81132
87526
92164
93261
100236
104765
111288
113672
118844
127882
130632
139001
143020
145716
151535
153449
157614
165484
178560
182475
185264
190188
191493
202627
206308
212462
214187
220647
226528
231108
234628
240353
246142
247163
256576
259053
264031
267505
270238
274564
276606
285205
291380
296123
301517
304503
307891
313080
322153
326331
329094
335584
341498
344819
353283
354590
357841
364991
370151
382674
385196
389038
394419
403278
416631
419038
422331
430447
434704
445323
452117
456454
458944
461645
464987
466426
475191
478433
481392

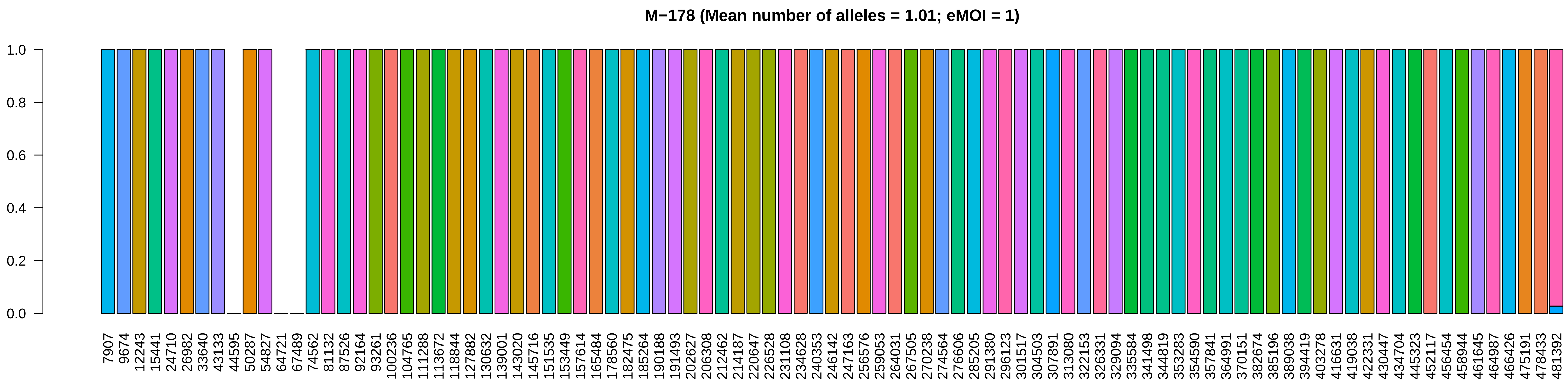
M−178 (Mean number of alleles = 1.01; eMOI = 1)
1.0
0.8
0.6
0.4
0.2
0.0
7907
9674
12243
15441
24710
26982
33640
43133
44595
50287
54827
64721
67489
74562
81132
87526
92164
93261
100236
104765
111288
113672
118844
127882
130632
139001
143020
145716
151535
153449
157614
165484
178560
182475
185264
190188
191493
202627
206308
212462
214187
220647
226528
231108
234628
240353
246142
247163
256576
259053
264031
267505
270238
274564
276606
285205
291380
296123
301517
304503
307891
313080
322153
326331
329094
335584
341498
344819
353283
354590
357841
364991
370151
382674
385196
389038
394419
403278
416631
419038
422331
430447
434704
445323
452117
456454
458944
461645
464987
466426
475191
478433
481392

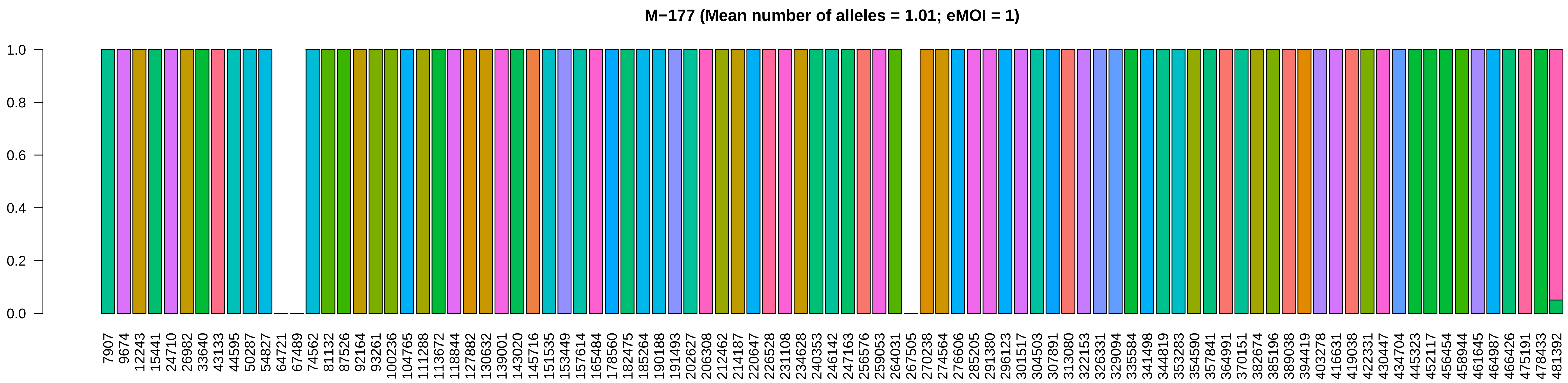
M−177 (Mean number of alleles = 1.01; eMOI = 1)
1.0
0.8
0.6
0.4
0.2
0.0
7907
9674
12243
15441
24710
26982
33640
43133
44595
50287
54827
64721
67489
74562
81132
87526
92164
93261
100236
104765
111288
113672
118844
127882
130632
139001
143020
145716
151535
153449
157614
165484
178560
182475
185264
190188
191493
202627
206308
212462
214187
220647
226528
231108
234628
240353
246142
247163
256576
259053
264031
267505
270238
274564
276606
285205
291380
296123
301517
304503
307891
313080
322153
326331
329094
335584
341498
344819
353283
354590
357841
364991
370151
382674
385196
389038
394419
403278
416631
419038
422331
430447
434704
445323
452117
456454
458944
461645
464987
466426
475191
478433
481392

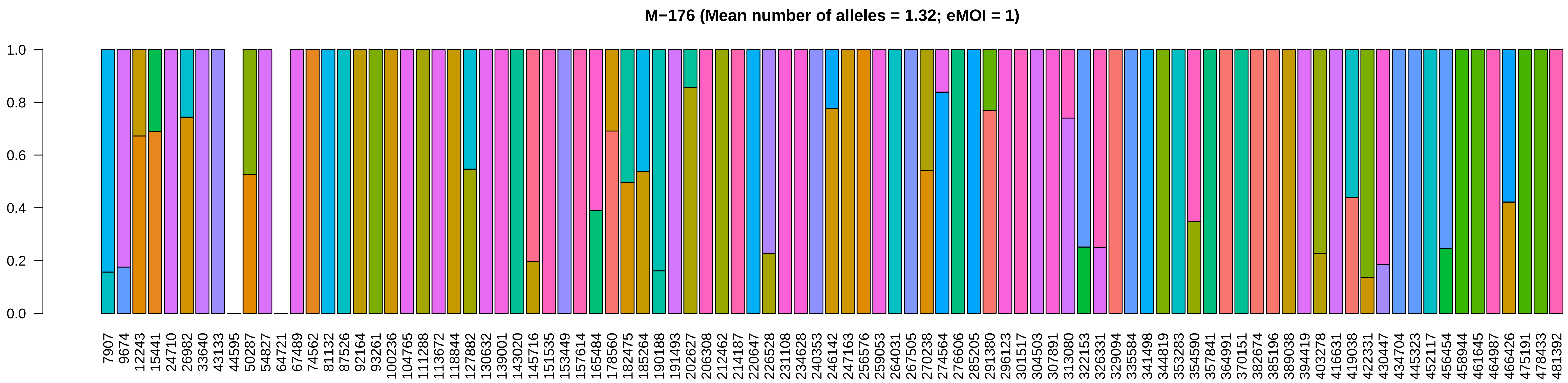
M−176 (Mean number of alleles = 1.32; eMOI = 1)
1.0
0.8
0.6
0.4
0.2
0.0
7907 9674 12243 15441 24710 26982 33640 43133 44595 50287 54827 64721 67489 74562 81132 87526 92164 93261 100236 104765 111288 113672 118844 127882 130632 139001 143020 145716 151535 153449 157614 165484 178560 182475 185264 190188 191493 202627 206308 212462 214187 220647 226528 231108 234628 240353 246142 247163 256576 259053 264031 267505 270238 274564 276606 285205 291380 296123 301517 304503 307891 313080 322153 326331 329094 335584 341498 344819 353283 354590 357841 364991 370151 382674 385196 389038 394419 403278 416631 419038 422331 430447 434704 445323 452117 456454 458944 461645 464987 466426 475191 478433 481392

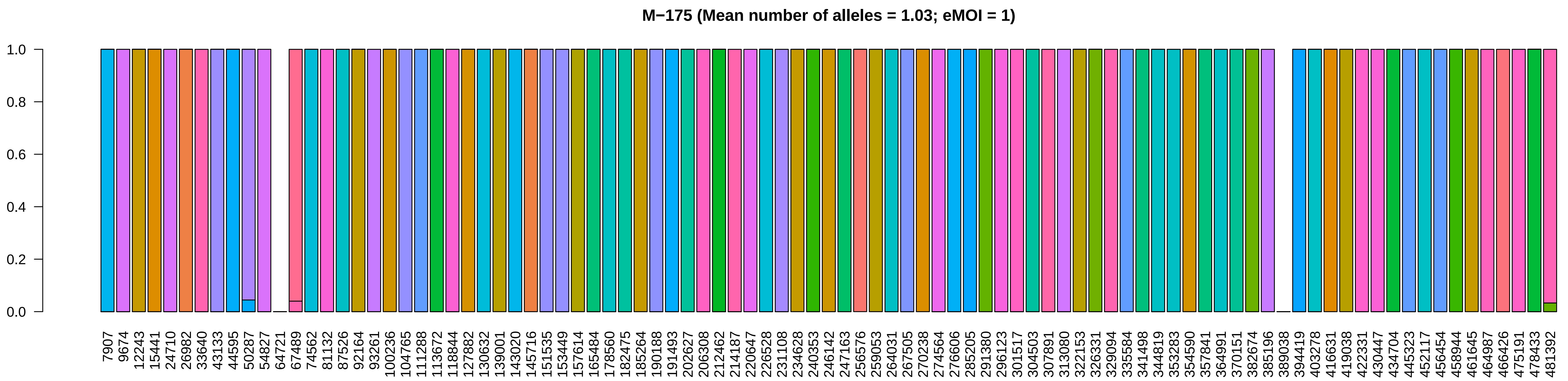

M−175 (Mean number of alleles = 1.03; eMOI = 1)
0.0
0.2
0.4
0.6
0.8
1.0
7907
9674
12243
15441
24710
26982
33640
43133
44595
50287
54827
64721
67489
74562
81132
87526
92164
93261
100236
104765
111288
113672
118844
127882
130632
139001
143020
145716
151535
153449
157614
165484
178560
182475
185264
190188
191493
202627
206308
212462
214187
220647
226528
231108
234628
240353
246142
247163
256576
259053
264031
267505
270238
274564
276606
285205
291380
296123
301517
304503
307891
313080
322153
326331
329094
335584
341498
344819
353283
354590
357841
364991
370151
382674
385196
389038
394419
403278
416631
419038
422331
430447
434704
445323
452117
456454
458944
461645
464987
466426
475191
478433
481392

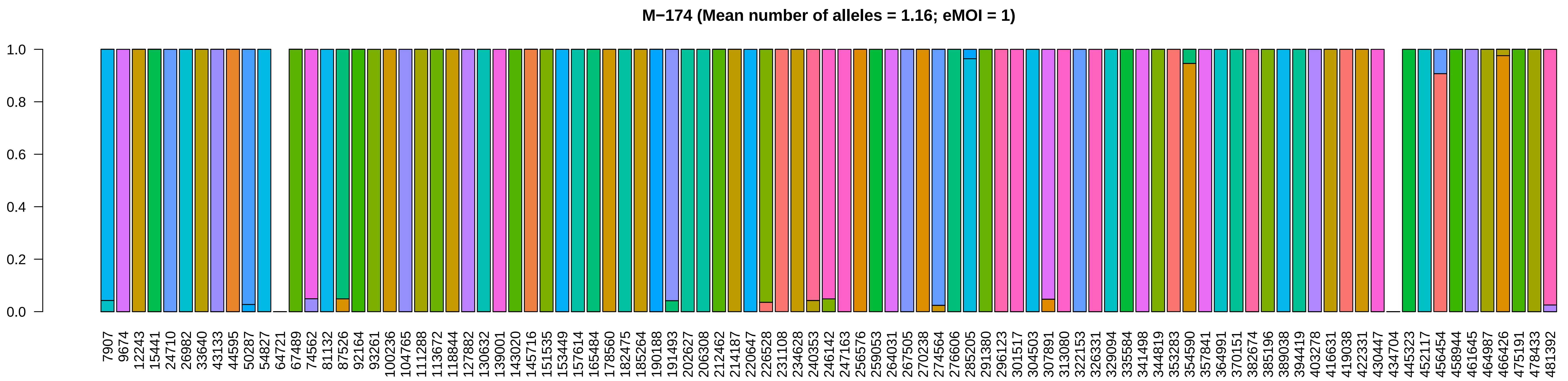
M−174 (Mean number of alleles = 1.16; eMOI = 1)
1.0
0.8
0.6
0.4
0.2
0.0
7907 9674 12243 15441 24710 26982 33640 43133 44595 50287 54827 64721 67489 74562 81132 87526 92164 93261 100236 104765 111288 113672 118844 127882 130632 139001 143020 145716 151535 153449 157614 165484 178560 182475 185264 190188 191493 202627 206308 212462 214187 220647 226528 231108 234628 240353 246142 247163 256576 259053 264031 267505 270238 274564 276606 285205 291380 296123 301517 304503 307891 313080 322153 326331 329094 335584 341498 344819 353283 354590 357841 364991 370151 382674 385196 389038 394419 403278 416631 419038 422331 430447 434704 445323 452117 456454 458944 461645 464987 466426 475191 478433 481392

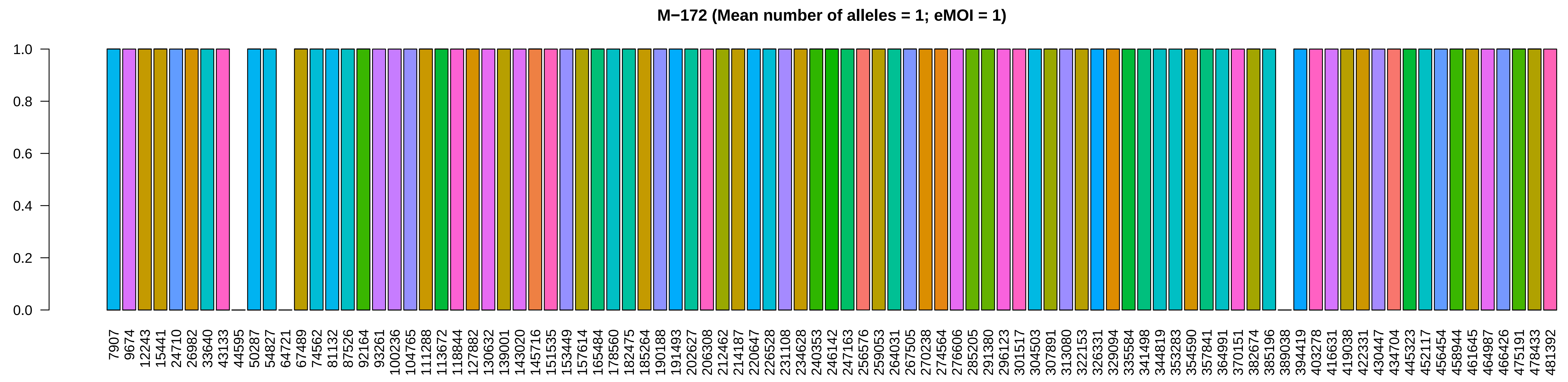

M−172 (Mean number of alleles = 1; eMOI = 1)
1.0
0.8
0.6
0.4
0.2
0.0
7907
9674
12243
15441
24710
26982
33640
43133
44595
50287
54827
64721
67489
74562
81132
87526
92164
93261
100236
104765
111288
113672
118844
127882
130632
139001
143020
145716
151535
153449
157614
165484
178560
182475
185264
190188
191493
202627
206308
212462
214187
220647
226528
231108
234628
240353
246142
247163
256576
259053
264031
267505
270238
274564
276606
285205
291380
296123
301517
304503
307891
313080
322153
326331
329094
335584
341498
344819
353283
354590
357841
364991
370151
382674
385196
389038
394419
403278
416631
419038
422331
430447
434704
445323
452117
456454
458944
461645
464987
466426
475191
478433
481392

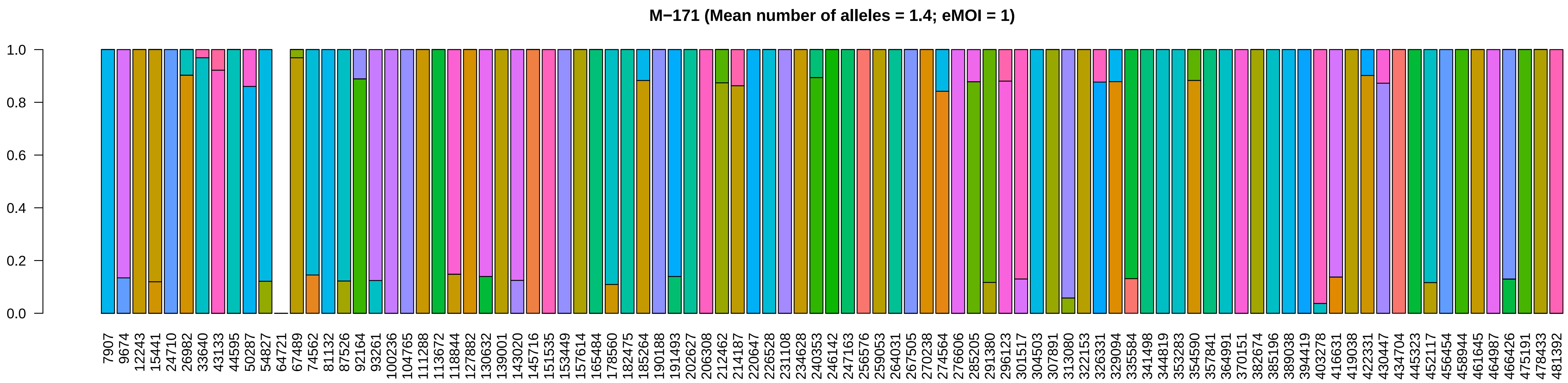

M−171 (Mean number of alleles = 1.4; eMOI = 1)
0.0
0.2
0.4
0.6
0.8
1.0
7907
9674
12243
15441
24710
26982
33640
43133
44595
50287
54827
64721
67489
74562
81132
87526
92164
93261
100236
104765
111288
113672
118844
127882
130632
139001
143020
145716
151535
153449
157614
165484
178560
182475
185264
190188
191493
202627
206308
212462
214187
220647
226528
231108
234628
240353
246142
247163
256576
259053
264031
267505
270238
274564
276606
285205
291380
296123
301517
304503
307891
313080
322153
326331
329094
335584
341498
344819
353283
354590
357841
364991
370151
382674
385196
389038
394419
403278
416631
419038
422331
430447
434704
445323
452117
456454
458944
461645
464987
466426
475191
478433
481392

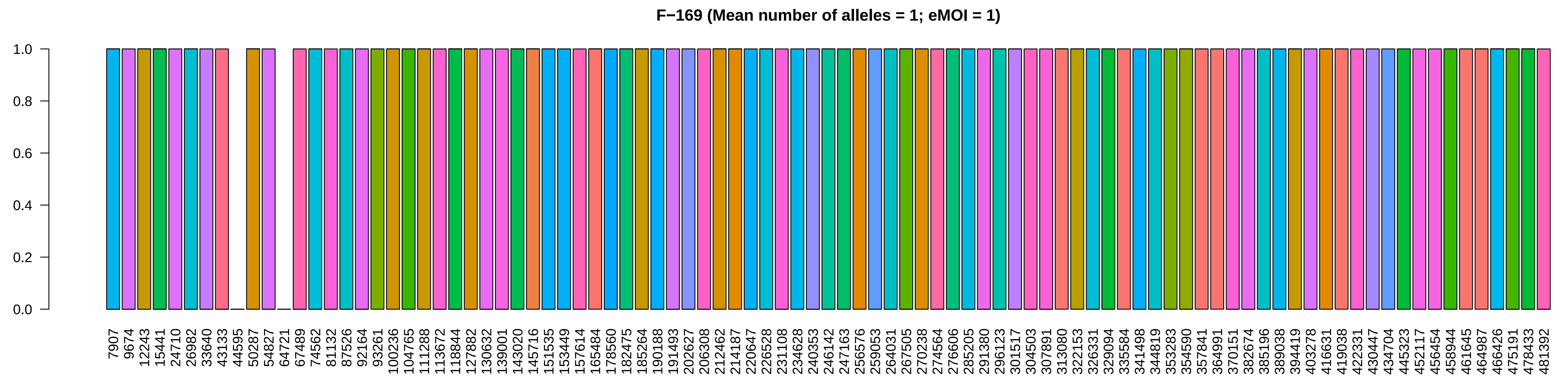

F−169 (Mean number of alleles = 1; eMOI = 1)
1.0
0.8
0.6
0.4
0.2
0.0
7907 9674 12243 15441 24710 26982 33640 43133 44595 50287 54827 64721 67489 74562 81132 87526 92164 93261 100236 104765 111288 113672 118844 127882 130632 139001 143020 145716 151535 153449 157614 165484 178560 182475 185264 190188 191493 202627 206308 212462 214187 220647 226528 231108 234628 240353 246142 247163 256576 259053 264031 267505 270238 274564 276606 285205 291380 296123 301517 304503 307891 313080 322153 326331 329094 335584 341498 344819 353283 354590 357841 364991 370151 382674 385196 389038 394419 403278 416631 419038 422331 430447 434704 445323 452117 456454 458944 461645 464987 466426 475191 478433 481392

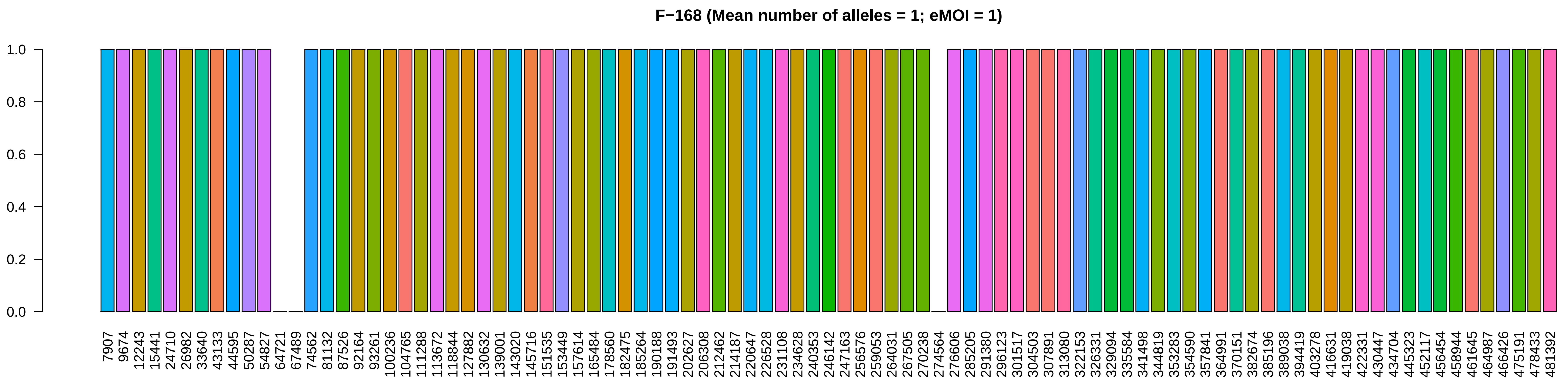
F−168 (Mean number of alleles = 1; eMOI = 1)
1.0
0.8
0.6
0.4
0.2
0.0
7907
9674
12243
15441
24710
26982
33640
43133
44595
50287
54827
64721
67489
74562
81132
87526
92164
93261
100236
104765
111288
113672
118844
127882
130632
139001
143020
145716
151535
153449
157614
165484
178560
182475
185264
190188
191493
202627
206308
212462
214187
220647
226528
231108
234628
240353
246142
247163
256576
259053
264031
267505
270238
274564
276606
285205
291380
296123
301517
304503
307891
313080
322153
326331
329094
335584
341498
344819
353283
354590
357841
364991
370151
382674
385196
389038
394419
403278
416631
419038
422331
430447
434704
445323
452117
456454
458944
461645
464987
466426
475191
478433
481392

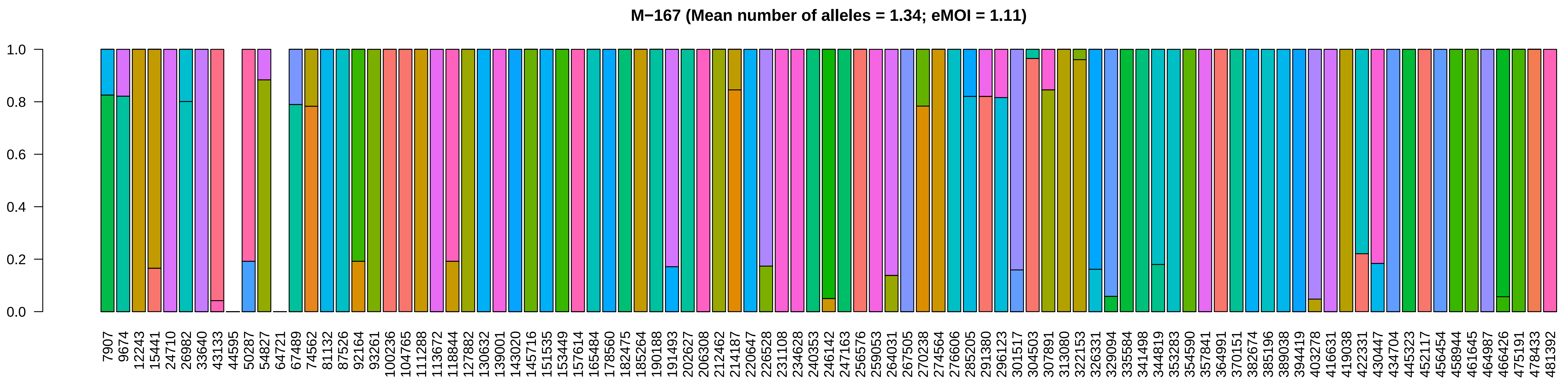

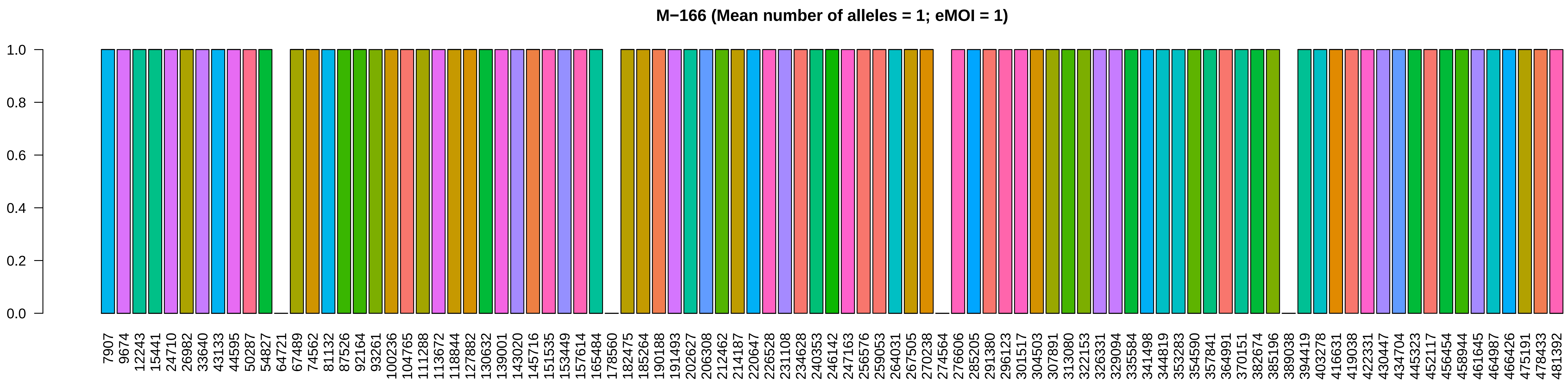
M−166 (Mean number of alleles = 1; eMOI = 1)
1.0
0.8
0.6
0.4
0.2
0.0
7907
9674
12243
15441
24710
26982
33640
43133
44595
50287
54827
64721
67489
74562
81132
87526
92164
93261
100236
104765
111288
113672
118844
127882
130632
139001
143020
145716
151535
153449
157614
165484
178560
182475
185264
190188
191493
202627
206308
212462
214187
220647
226528
231108
234628
240353
246142
247163
256576
259053
264031
267505
270238
274564
276606
285205
291380
296123
301517
304503
307891
313080
322153
326331
329094
335584
341498
344819
353283
354590
357841
364991
370151
382674
385196
389038
394419
403278
416631
419038
422331
430447
434704
445323
452117
456454
458944
461645
464987
466426
475191
478433
481392

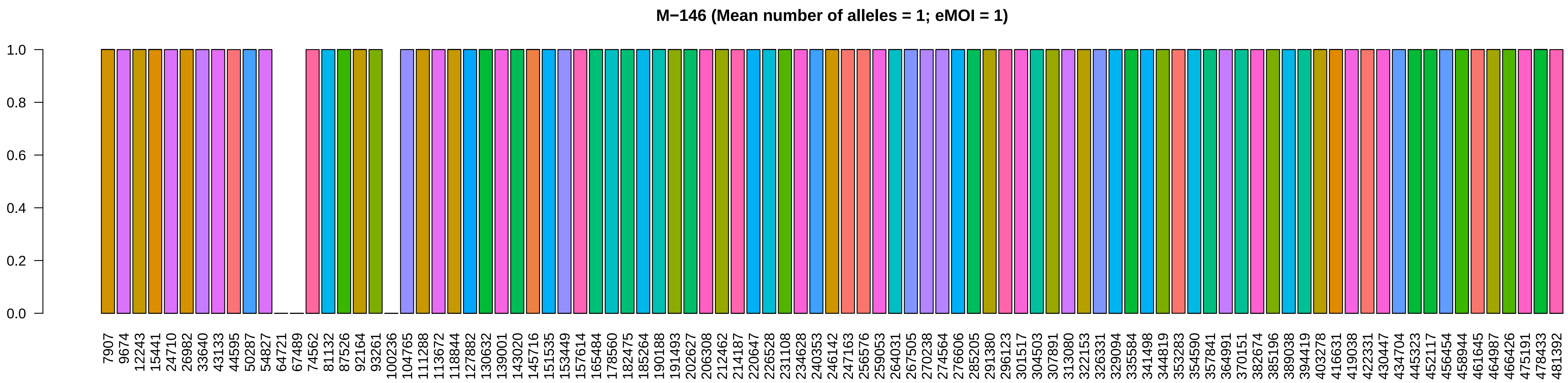

M−146 (Mean number of alleles = 1; eMOI = 1)
1.0
0.8
0.6
0.4
0.2
0.0
7907
9674
12243
15441
24710
26982
33640
43133
44595
50287
54827
64721
67489
74562
81132
87526
92164
93261
100236
104765
111288
113672
118844
127882
130632
139001
143020
145716
151535
153449
157614
165484
178560
182475
185264
190188
191493
202627
206308
212462
214187
220647
226528
231108
234628
240353
246142
247163
256576
259053
264031
267505
270238
274564
276606
285205
291380
296123
301517
304503
307891
313080
322153
326331
329094
335584
341498
344819
353283
354590
357841
364991
370151
382674
385196
389038
394419
403278
416631
419038
422331
430447
434704
445323
452117
456454
458944
461645
464987
466426
475191
478433
481392

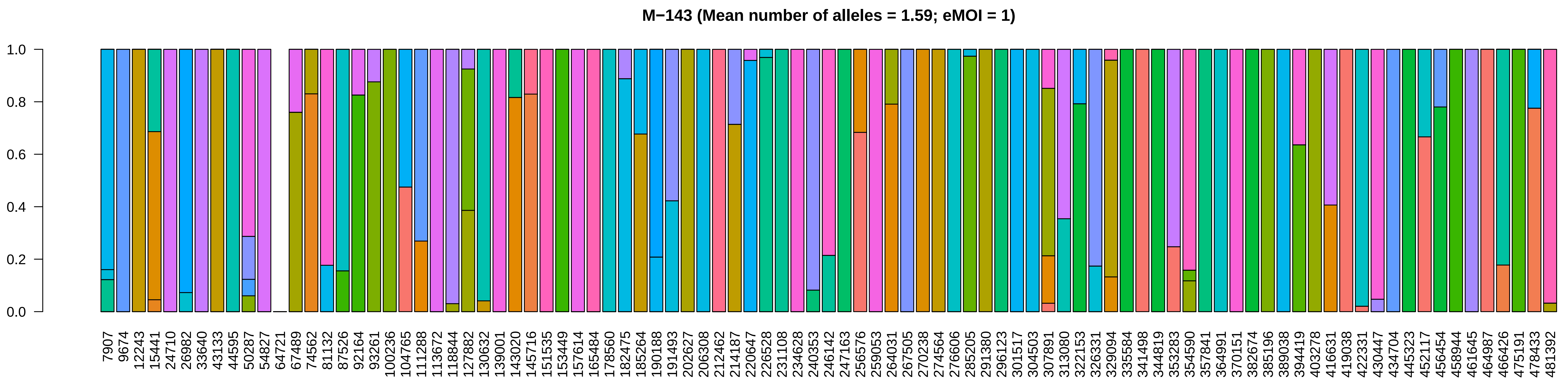

M−143 (Mean number of alleles = 1.59; eMOI = 1)
1.0
0.8
0.6
0.4
0.2
0.0
7907
9674
12243
15441
24710
26982
33640
43133
44595
50287
54827
64721
67489
74562
81132
87526
92164
93261
100236
104765
111288
113672
118844
127882
130632
139001
143020
145716
151535
153449
157614
165484
178560
182475
185264
190188
191493
202627
206308
212462
214187
220647
226528
231108
234628
240353
246142
247163
256576
259053
264031
267505
270238
274564
276606
285205
291380
296123
301517
304503
307891
313080
322153
326331
329094
335584
341498
344819
353283
354590
357841
364991
370151
382674
385196
389038
394419
403278
416631
419038
422331
430447
434704
445323
452117
456454
458944
461645
464987
466426
475191
478433
481392

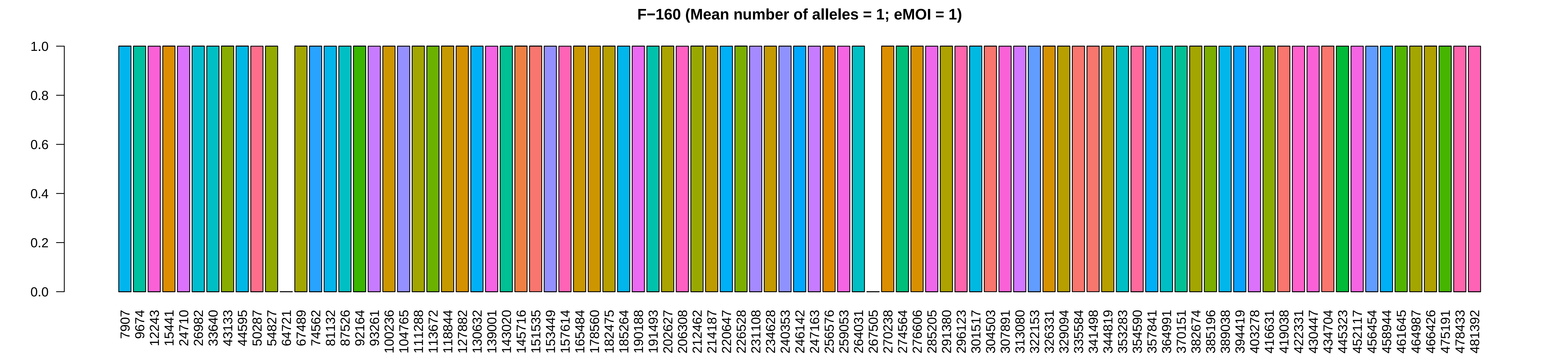
F−160 (Mean number of alleles = 1; eMOI = 1)
1.0
0.8
0.6
0.4
0.2
0.0
7907
9674
12243
15441
24710
26982
33640
43133
44595
50287
54827
64721
67489
74562
81132
87526
92164
93261
100236
104765
111288
113672
118844
127882
130632
139001
143020
145716
151535
153449
157614
165484
178560
182475
185264
190188
191493
202627
206308
212462
214187
220647
226528
231108
234628
240353
246142
247163
256576
259053
264031
267505
270238
274564
276606
285205
291380
296123
301517
304503
307891
313080
322153
326331
329094
335584
341498
344819
353283
354590
357841
364991
370151
382674
385196
389038
394419
403278
416631
419038
422331
430447
434704
445323
452117
456454
458944
461645
464987
466426
475191
478433
481392

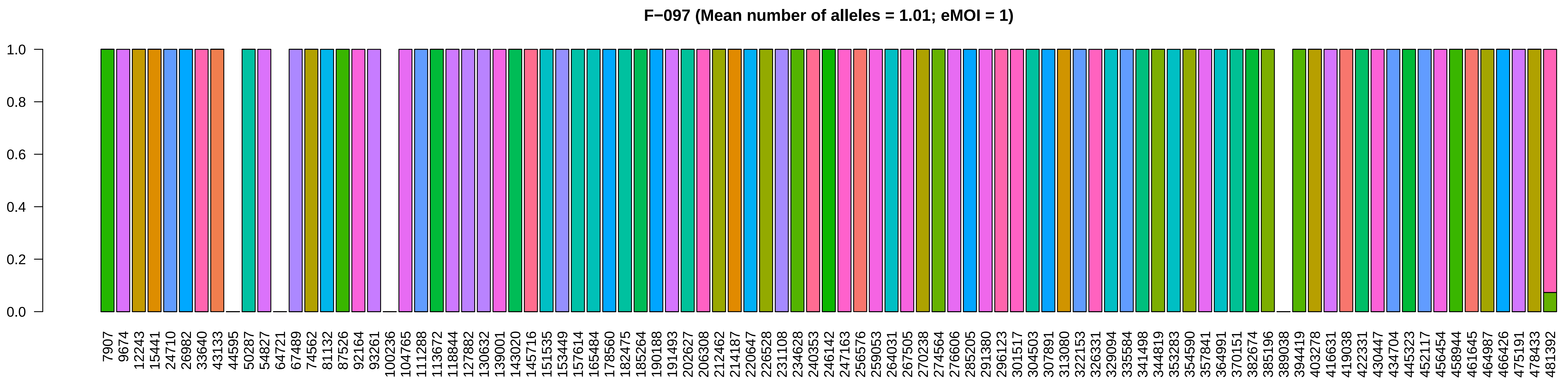
F−097 (Mean number of alleles = 1.01; eMOI = 1)
0.0
0.2
0.4
0.6
0.8
1.0
7907 9674 12243 15441 24710 26982 33640 43133 44595 50287 54827 64721 67489 74562 81132 87526 92164 93261 100236 104765 111288 113672 118844 127882 130632 139001 143020 145716 151535 153449 157614 165484 178560 182475 185264 190188 191493 202627 206308 212462 214187 220647 226528 231108 234628 240353 246142 247163 256576 259053 264031 267505 270238 274564 276606 285205 291380 296123 301517 304503 307891 313080 322153 326331 329094 335584 341498 344819 353283 354590 357841 364991 370151 382674 385196 389038 394419 403278 416631 419038 422331 430447 434704 445323 452117 456454 458944 461645 464987 466426 475191 478433 481392

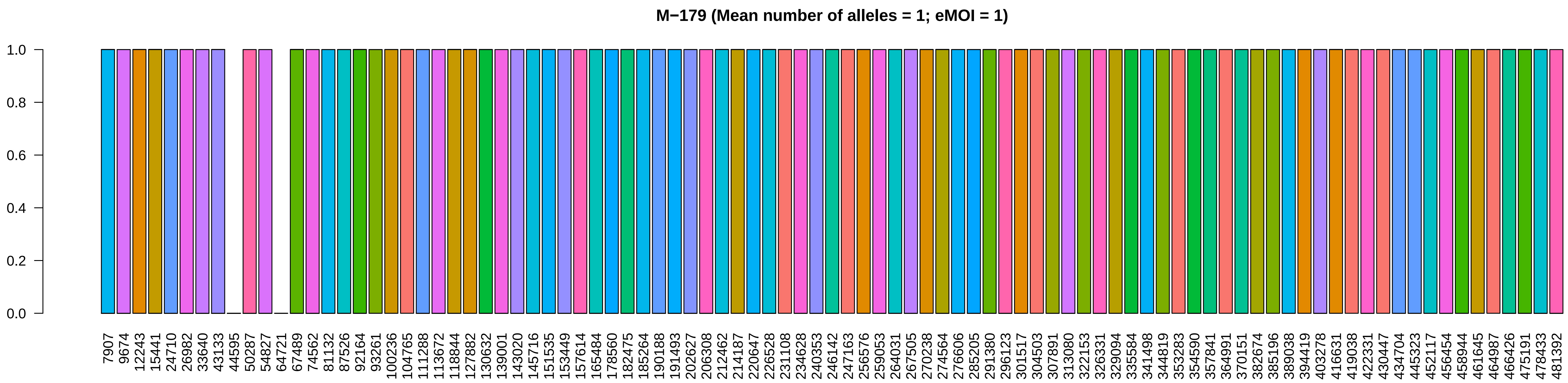
M−179 (Mean number of alleles = 1; eMOI = 1)
1.0
0.8
0.6
0.4
0.2
0.0
7907
9674
12243
15441
24710
26982
33640
43133
44595
50287
54827
64721
67489
74562
81132
87526
92164
93261
100236
104765
111288
113672
118844
127882
130632
139001
143020
145716
151535
153449
157614
165484
178560
182475
185264
190188
191493
202627
206308
212462
214187
220647
226528
231108
234628
240353
246142
247163
256576
259053
264031
267505
270238
274564
276606
285205
291380
296123
301517
304503
307891
313080
322153
326331
329094
335584
341498
344819
353283
354590
357841
364991
370151
382674
385196
389038
394419
403278
416631
419038
422331
430447
434704
445323
452117
456454
458944
461645
464987
466426
475191
478433
481392

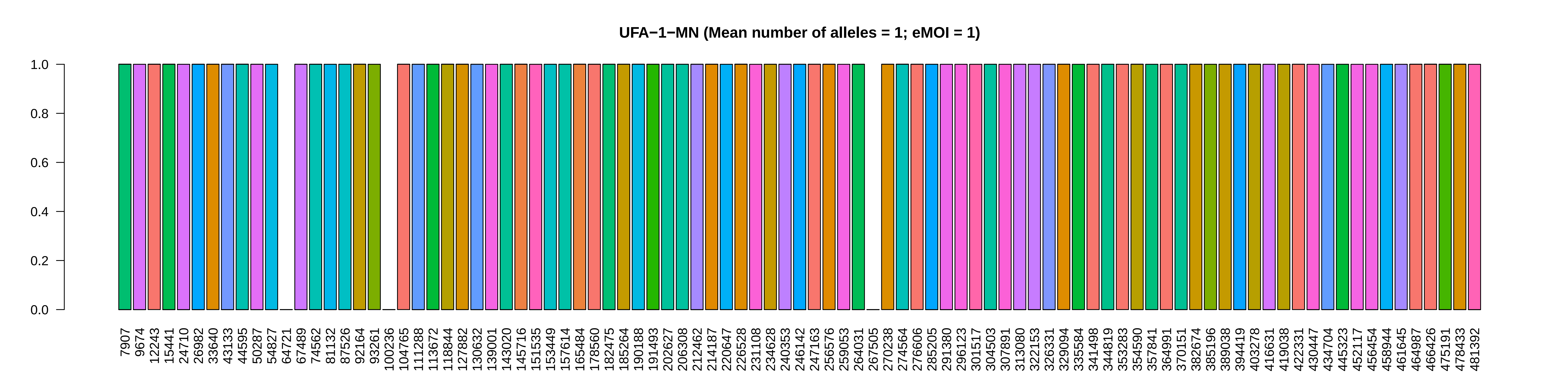
UFA−1−MN (Mean number of alleles = 1; eMOI = 1)
1.0
0.8
0.6
0.4
0.2
0.0
7907
9674
12243
15441
24710
26982
33640
43133
44595
50287
54827
64721
67489
74562
81132
87526
92164
93261
100236
104765
111288
113672
118844
127882
130632
139001
143020
145716
151535
153449
157614
165484
178560
182475
185264
190188
191493
202627
206308
212462
214187
220647
226528
231108
234628
240353
246142
247163
256576
259053
264031
267505
270238
274564
276606
285205
291380
296123
301517
304503
307891
313080
322153
326331
329094
335584
341498
344819
353283
354590
357841
364991
370151
382674
385196
389038
394419
403278
416631
419038
422331
430447
434704
445323
452117
456454
458944
461645
464987
466426
475191
478433
481392

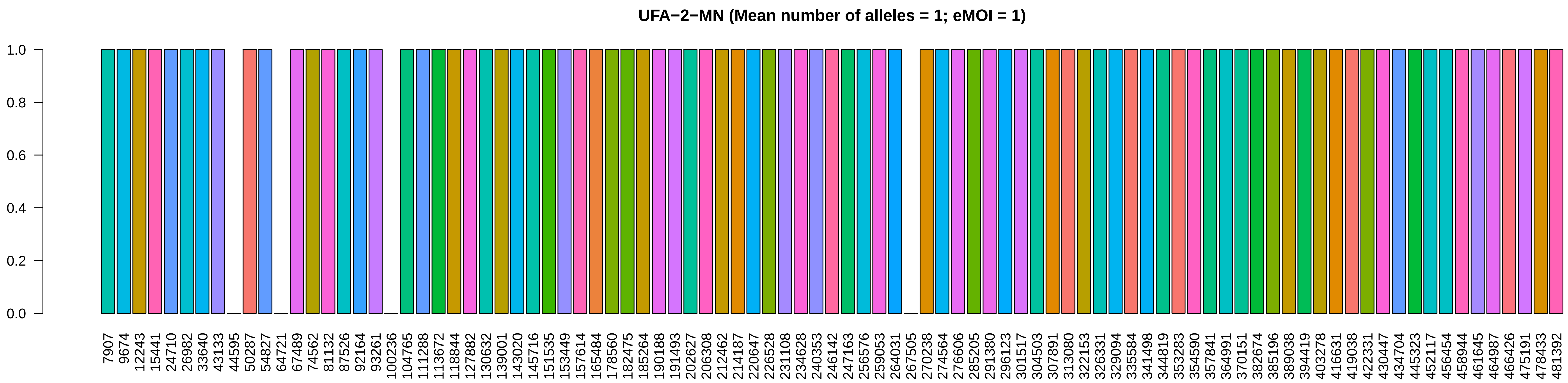

UFA−2−MN (Mean number of alleles = 1; eMOI = 1)
0.0
0.2
0.4
0.6
0.8
1.0
7907
9674
12243
15441
24710
26982
33640
43133
44595
50287
54827
64721
67489
74562
81132
87526
92164
93261
100236
104765
111288
113672
118844
127882
130632
139001
143020
145716
151535
153449
157614
165484
178560
182475
185264
190188
191493
202627
206308
212462
214187
220647
226528
231108
234628
240353
246142
247163
256576
259053
264031
267505
270238
274564
276606
285205
291380
296123
301517
304503
307891
313080
322153
326331
329094
335584
341498
344819
353283
354590
357841
364991
370151
382674
385196
389038
394419
403278
416631
419038
422331
430447
434704
445323
452117
456454
458944
461645
464987
466426
475191
478433
481392

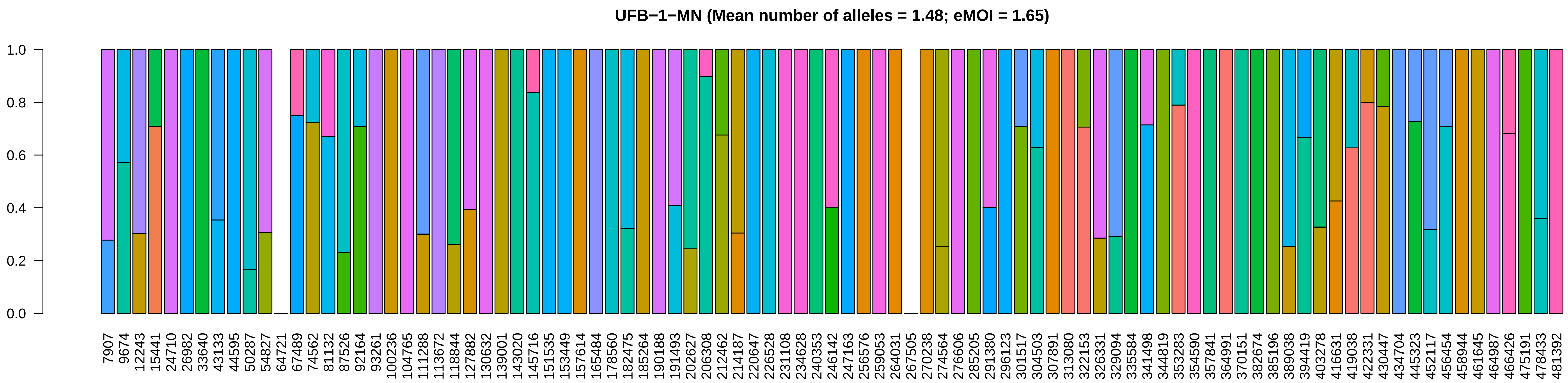
UFB−1−MN (Mean number of alleles = 1.48; eMOI = 1.65)
1.0
0.8
0.6
0.4
0.2
0.0
7907
9674
12243
15441
24710
26982
33640
43133
44595
50287
54827
64721
67489
74562
81132
87526
92164
93261
100236
104765
111288
113672
118844
127882
130632
139001
143020
145716
151535
153449
157614
165484
178560
182475
185264
190188
191493
202627
206308
212462
214187
220647
226528
231108
234628
240353
246142
247163
256576
259053
264031
267505
270238
274564
276606
285205
291380
296123
301517
304503
307891
313080
322153
326331
329094
335584
341498
344819
353283
354590
357841
364991
370151
382674
385196
389038
394419
403278
416631
419038
422331
430447
434704
445323
452117
456454
458944
461645
464987
466426
475191
478433
481392

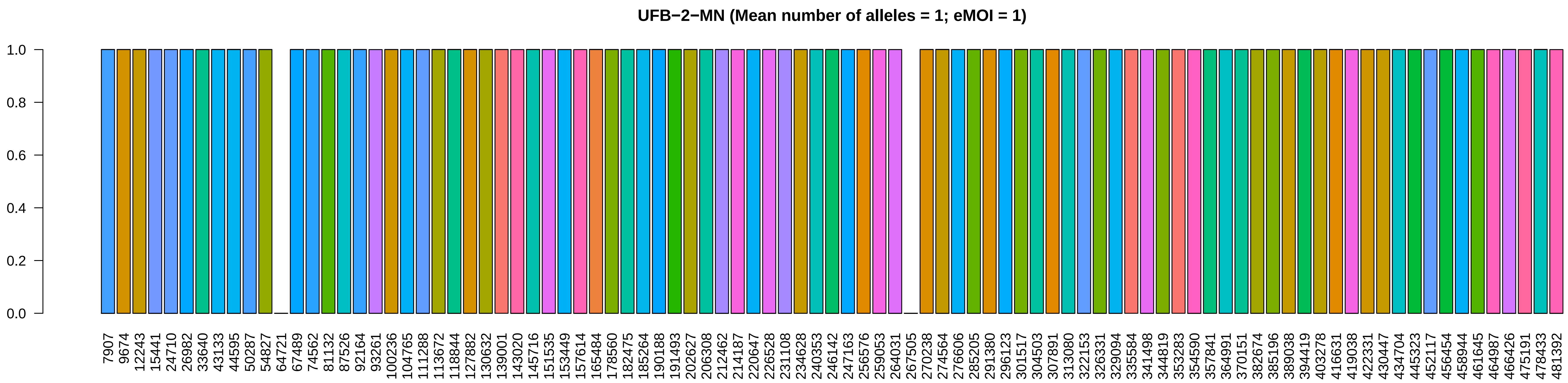

UFB−2−MN (Mean number of alleles = 1; eMOI = 1)
1.0
0.8
0.6
0.4
0.2
0.0
7907
9674
12243
15441
24710
26982
33640
43133
44595
50287
54827
64721
67489
74562
81132
87526
92164
93261
100236
104765
111288
113672
118844
127882
130632
139001
143020
145716
151535
153449
157614
165484
178560
182475
185264
190188
191493
202627
206308
212462
214187
220647
226528
231108
234628
240353
246142
247163
256576
259053
264031
267505
270238
274564
276606
285205
291380
296123
301517
304503
307891
313080
322153
326331
329094
335584
341498
344819
353283
354590
357841
364991
370151
382674
385196
389038
394419
403278
416631
419038
422331
430447
434704
445323
452117
456454
458944
461645
464987
466426
475191
478433
481392

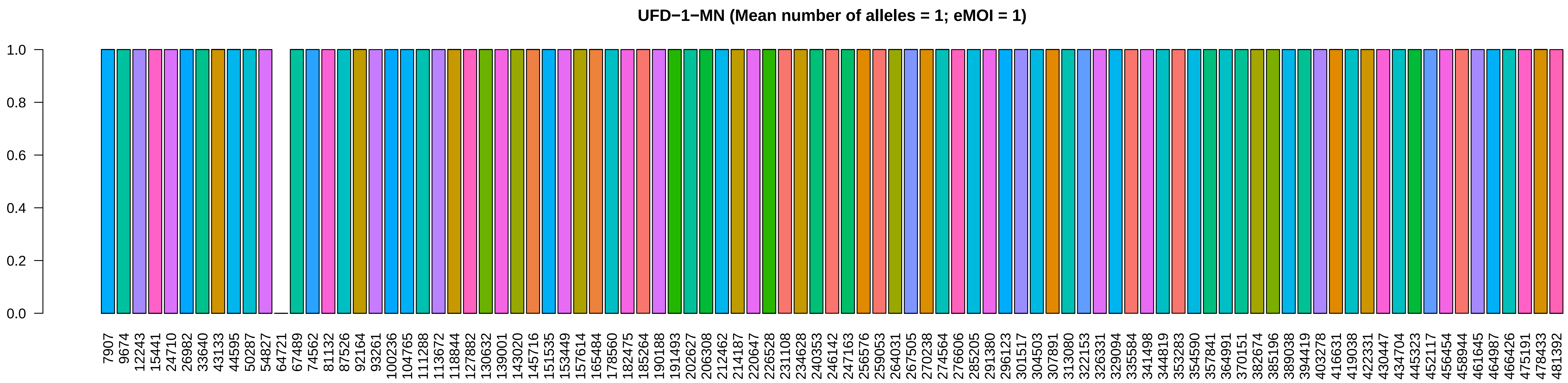

UFD−1−MN (Mean number of alleles = 1; eMOI = 1)
1.0
0.8
0.6
0.4
0.2
0.0
7907
9674
12243
15441
24710
26982
33640
43133
44595
50287
54827
64721
67489
74562
81132
87526
92164
93261
100236
104765
111288
113672
118844
127882
130632
139001
143020
145716
151535
153449
157614
165484
178560
182475
185264
190188
191493
202627
206308
212462
214187
220647
226528
231108
234628
240353
246142
247163
256576
259053
264031
267505
270238
274564
276606
285205
291380
296123
301517
304503
307891
313080
322153
326331
329094
335584
341498
344819
353283
354590
357841
364991
370151
382674
385196
389038
394419
403278
416631
419038
422331
430447
434704
445323
452117
456454
458944
461645
464987
466426
475191
478433
481392

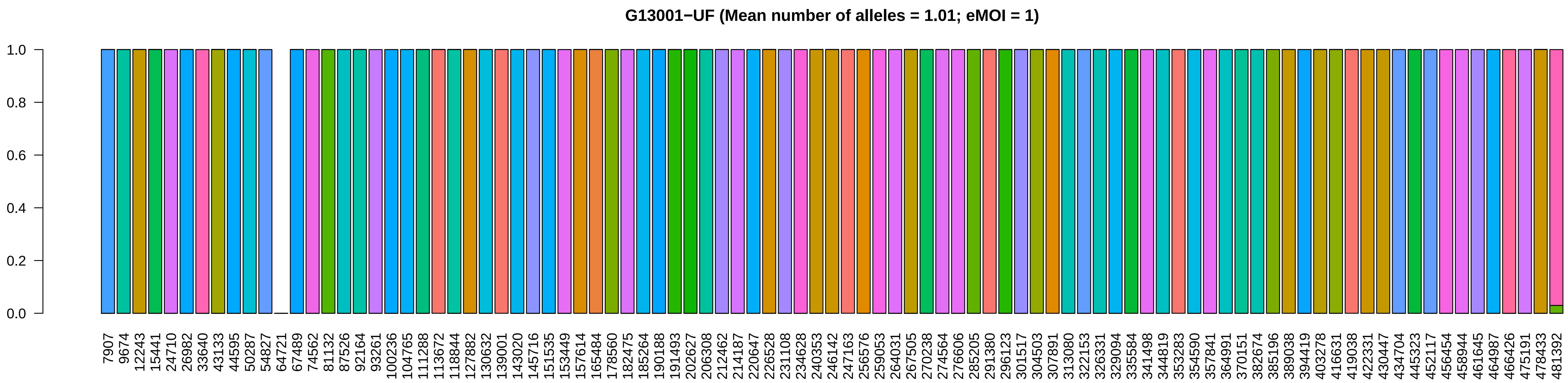

G13001−UF (Mean number of alleles = 1.01; eMOI = 1)
1.0
0.8
0.6
0.4
0.2
0.0
7907 9674 12243 15441 24710 26982 33640 43133 44595 50287 54827 64721 67489 74562 81132 87526 92164 93261 100236 104765 111288 113672 118844 127882 130632 139001 143020 145716 151535 153449 157614 165484 178560 182475 185264 190188 191493 202627 206308 212462 214187 220647 226528 231108 234628 240353 246142 247163 256576 259053 264031 267505 270238 274564 276606 285205 291380 296123 301517 304503 307891 313080 322153 326331 329094 335584 341498 344819 353283 354590 357841 364991 370151 382674 385196 389038 394419 403278 416631 419038 422331 430447 434704 445323 452117 456454 458944 461645 464987 466426 475191 478433 481392

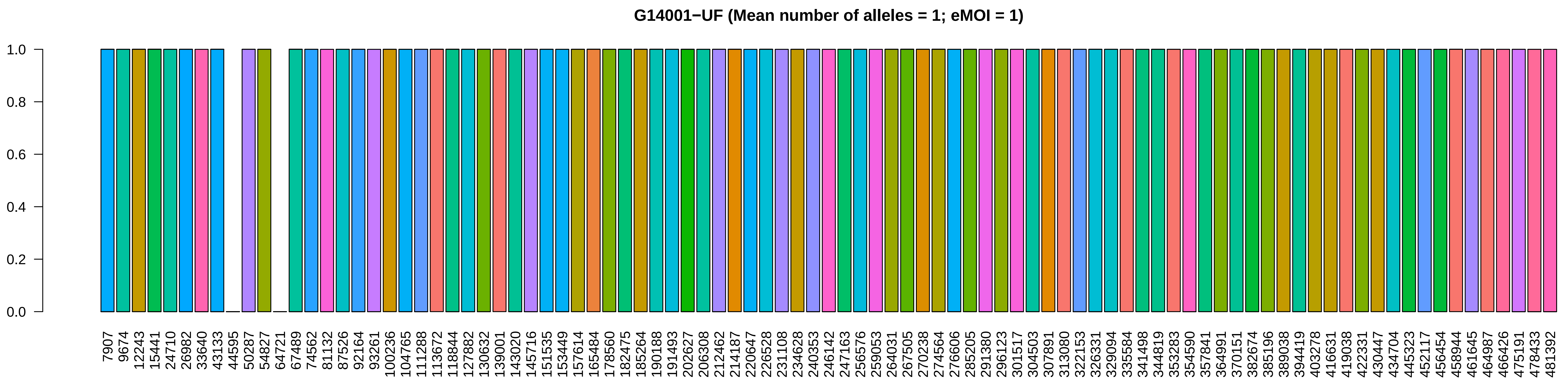
G14001−UF (Mean number of alleles = 1; eMOI = 1)
0.0
0.2
0.4
0.6
0.8
1.0
7907
9674
12243
15441
24710
26982
33640
43133
44595
50287
54827
64721
67489
74562
81132
87526
92164
93261
100236
104765
111288
113672
118844
127882
130632
139001
143020
145716
151535
153449
157614
165484
178560
182475
185264
190188
191493
202627
206308
212462
214187
220647
226528
231108
234628
240353
246142
247163
256576
259053
264031
267505
270238
274564
276606
285205
291380
296123
301517
304503
307891
313080
322153
326331
329094
335584
341498
344819
353283
354590
357841
364991
370151
382674
385196
389038
394419
403278
416631
419038
422331
430447
434704
445323
452117
456454
458944
461645
464987
466426
475191
478433
481392

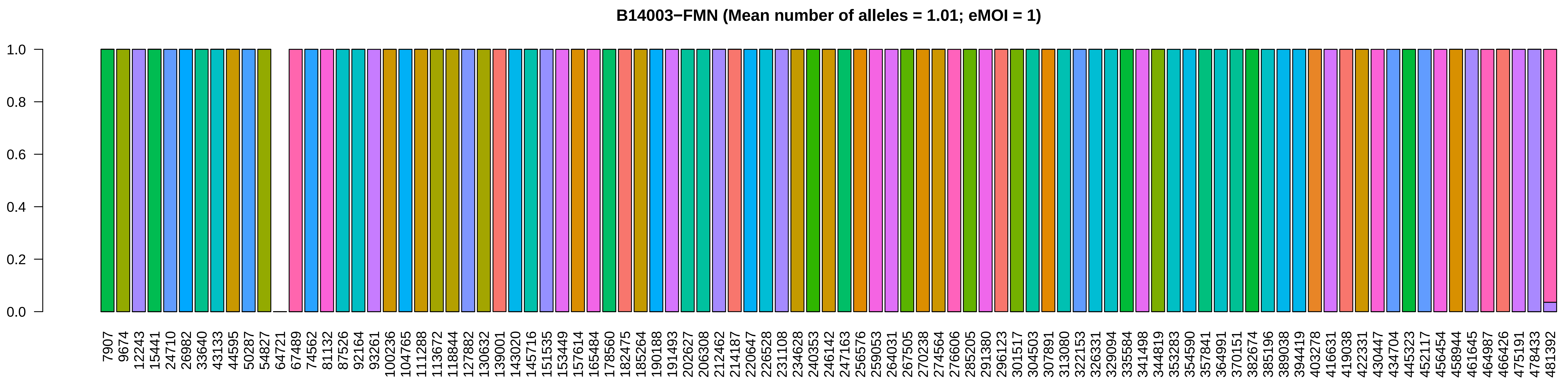
B14003−FMN (Mean number of alleles = 1.01; eMOI = 1)
1.0
0.8
0.6
0.4
0.2
0.0
7907 9674 12243 15441 24710 26982 33640 43133 44595 50287 54827 64721 67489 74562 81132 87526 92164 93261 100236 104765 111288 113672 118844 127882 130632 139001 143020 145716 151535 153449 157614 165484 178560 182475 185264 190188 191493 202627 206308 212462 214187 220647 226528 231108 234628 240353 246142 247163 256576 259053 264031 267505 270238 274564 276606 285205 291380 296123 301517 304503 307891 313080 322153 326331 329094 335584 341498 344819 353283 354590 357841 364991 370151 382674 385196 389038 394419 403278 416631 419038 422331 430447 434704 445323 452117 456454 458944 461645 464987 466426 475191 478433 481392

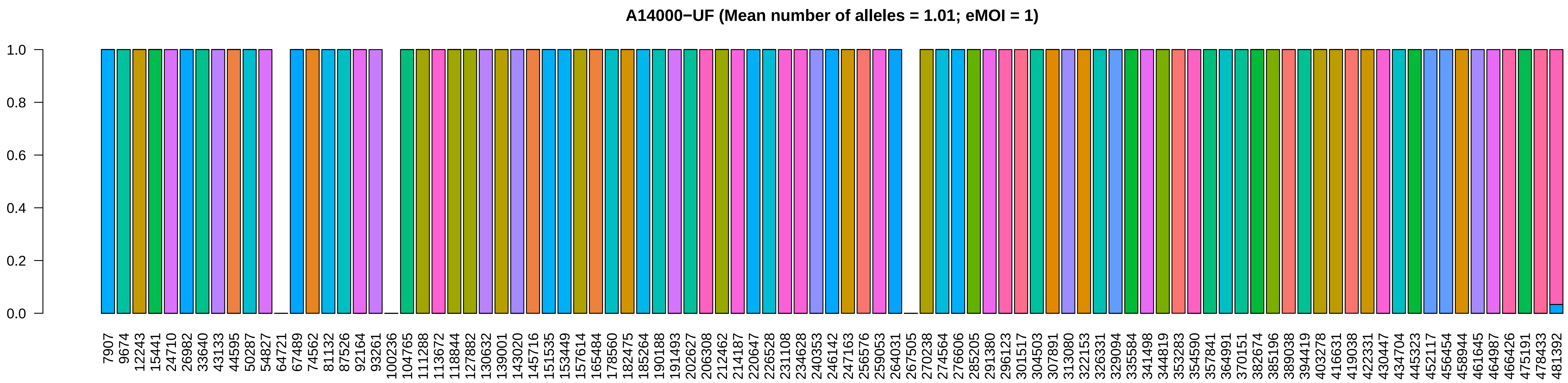

A14000−UF (Mean number of alleles = 1.01; eMOI = 1)

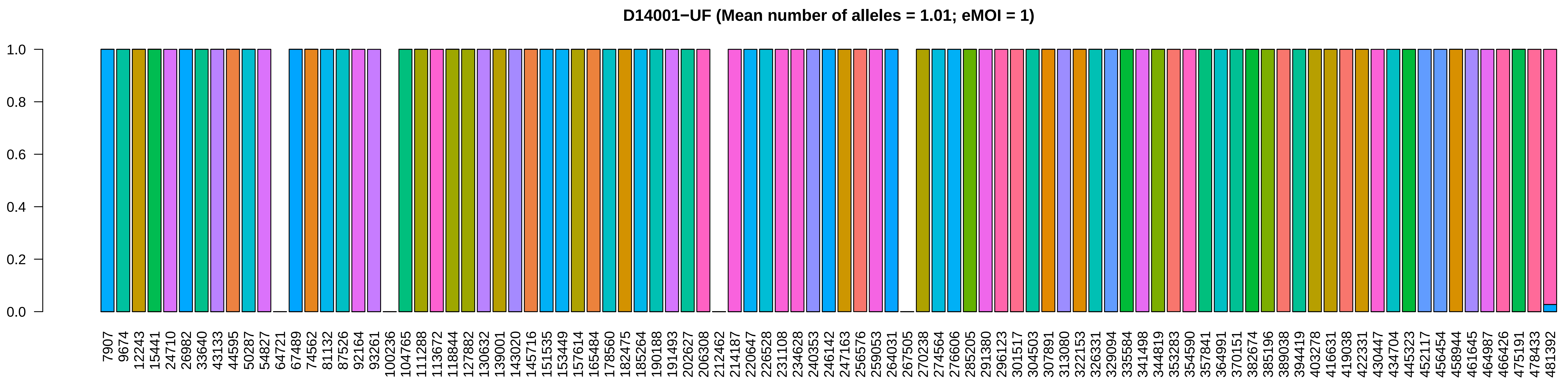
D14001−UF (Mean number of alleles = 1.01; eMOI = 1)
1.0
0.8
0.6
0.4
0.2
0.0

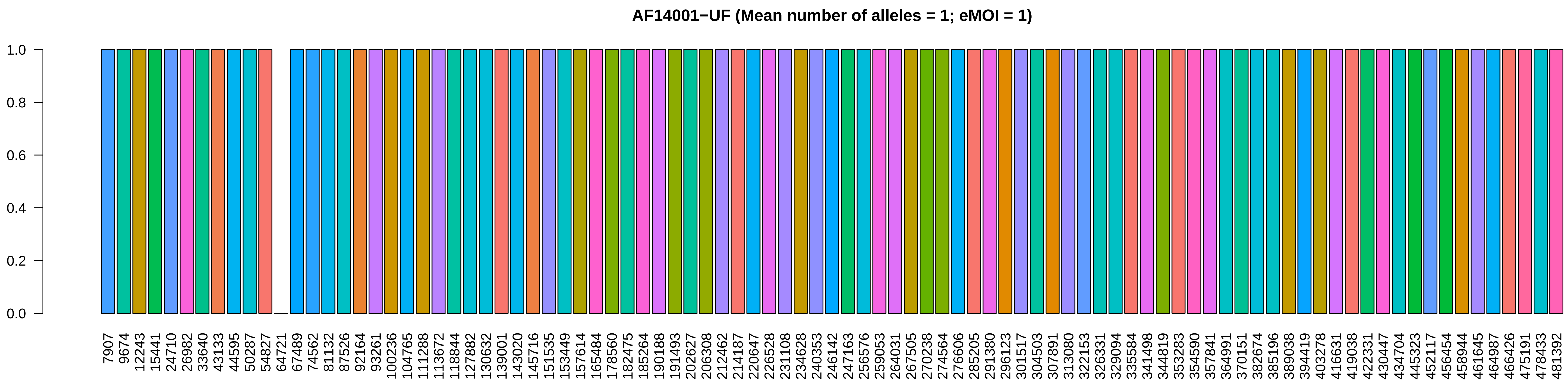

AF14001−UF (Mean number of alleles = 1; eMOI = 1)
1.0
0.8
0.6
0.4
0.2
0.0
7907 9674 12243 15441 24710 26982 33640 43133 44595 50287 54827 64721 67489 74562 81132 87526 92164 93261 100236 104765 111288 113672 118844 127882 130632 139001 143020 145716 151535 153449 157614 165484 178560 182475 185264 190188 191493 202627 206308 212462 214187 220647 226528 231108 234628 240353 246142 247163 256576 259053 264031 267505 270238 274564 276606 285205 291380 296123 301517 304503 307891 313080 322153 326331 329094 335584 341498 344819 353283 354590 357841 364991 370151 382674 385196 389038 394419 403278 416631 419038 422331 430447 434704 445323 452117 456454 458944 461645 464987 466426 475191 478433 481392

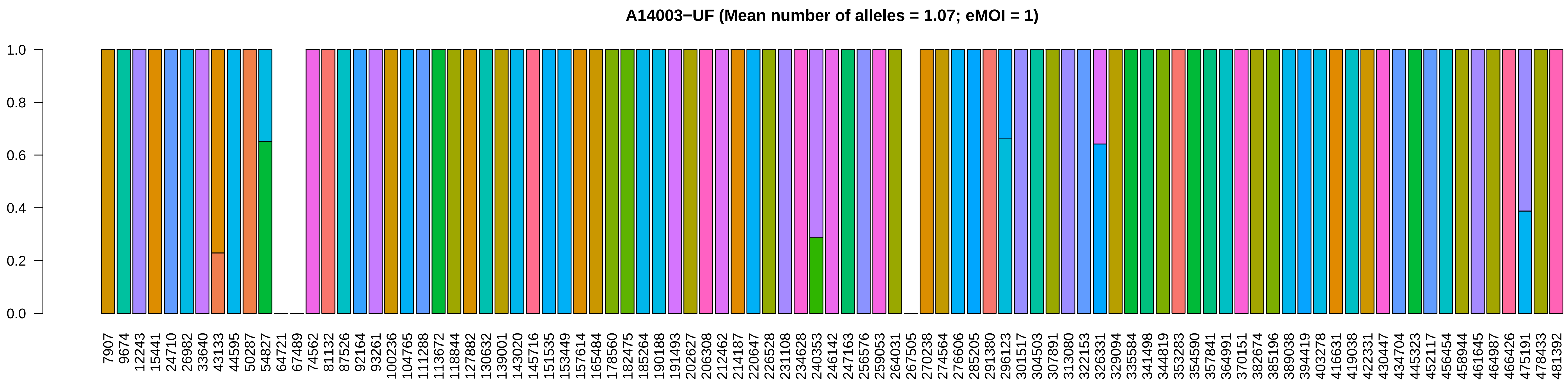
A14003−UF (Mean number of alleles = 1.07; eMOI = 1)
1.0
0.8
0.6
0.4
0.2
0.0
7907
9674
12243
15441
24710
26982
33640
43133
44595
50287
54827
64721
67489
74562
81132
87526
92164
93261
100236
104765
111288
113672
118844
127882
130632
139001
143020
145716
151535
153449
157614
165484
178560
182475
185264
190188
191493
202627
206308
212462
214187
220647
226528
231108
234628
240353
246142
247163
256576
259053
264031
267505
270238
274564
276606
285205
291380
296123
301517
304503
307891
313080
322153
326331
329094
335584
341498
344819
353283
354590
357841
364991
370151
382674
385196
389038
394419
403278
416631
419038
422331
430447
434704
445323
452117
456454
458944
461645
464987
466426
475191
478433
481392

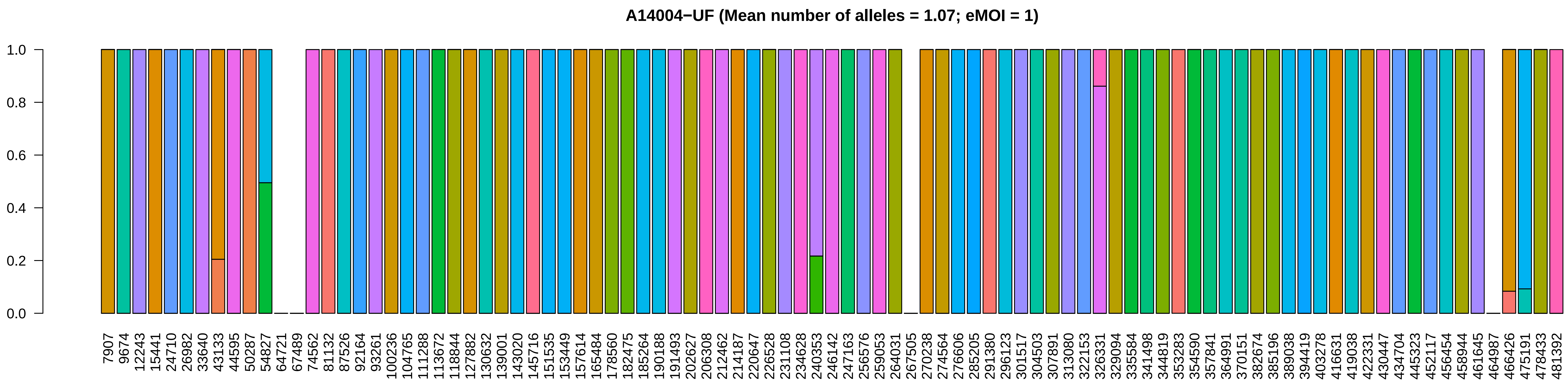

A14004−UF (Mean number of alleles = 1.07; eMOI = 1)
0.0
0.2
0.4
0.6
0.8
1.0
7907 9674 12243 15441 24710 26982 33640 43133 44595 50287 54827 64721 67489 74562 81132 87526 92164 93261 100236 104765 111288 113672 118844 127882 130632 139001 143020 145716 151535 153449 157614 165484 178560 182475 185264 190188 191493 202627 206308 212462 214187 220647 226528 231108 234628 240353 246142 247163 256576 259053 264031 267505 270238 274564 276606 285205 291380 296123 301517 304503 307891 313080 322153 326331 329094 335584 341498 344819 353283 354590 357841 364991 370151 382674 385196 389038 394419 403278 416631 419038 422331 430447 434704 445323 452117 456454 458944 461645 464987 466426 475191 478433 481392

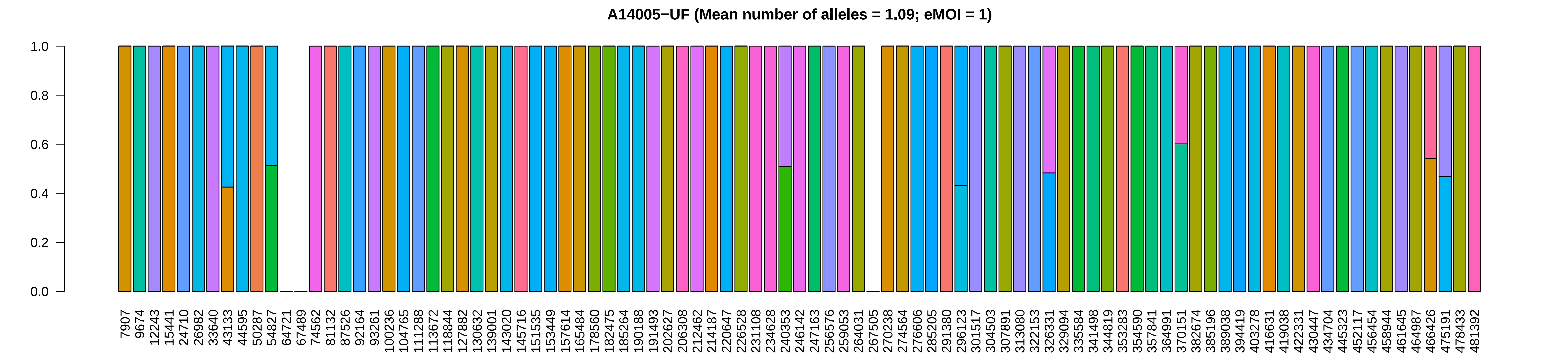

A14005−UF (Mean number of alleles = 1.09; eMOI = 1)
0.0
0.2
0.4
0.6
0.8
1.0
7907
9674
12243
15441
24710
26982
33640
43133
44595
50287
54827
64721
67489
74562
81132
87526
92164
93261
100236
104765
111288
113672
118844
127882
130632
139001
143020
145716
151535
153449
157614
165484
178560
182475
185264
190188
191493
202627
206308
212462
214187
220647
226528
231108
234628
240353
246142
247163
256576
259053
264031
267505
270238
274564
276606
285205
291380
296123
301517
304503
307891
313080
322153
326331
329094
335584
341498
344819
353283
354590
357841
364991
370151
382674
385196
389038
394419
403278
416631
419038
422331
430447
434704
445323
452117
456454
458944
461645
464987
466426
475191
478433
481392

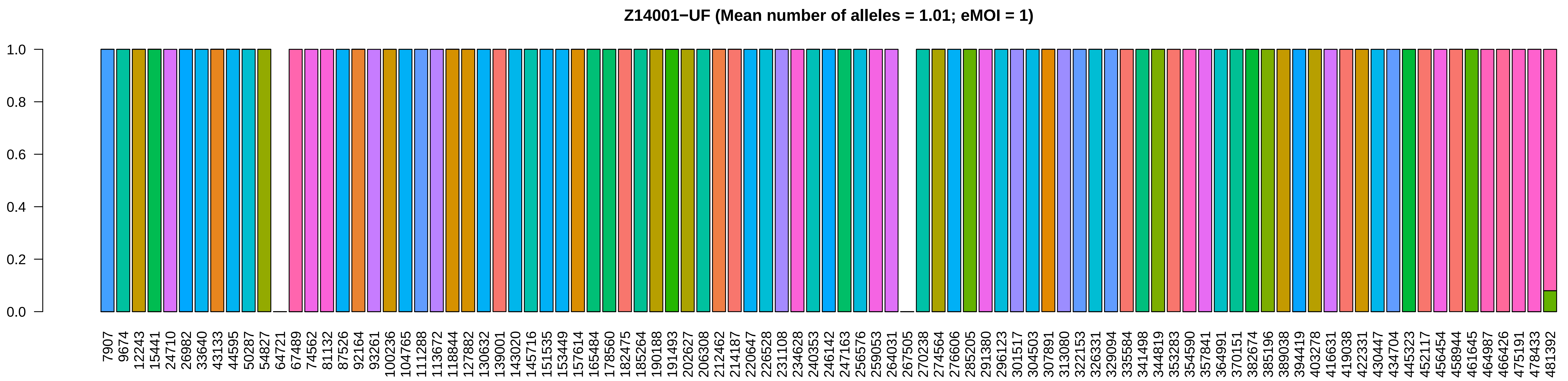
Z14001−UF (Mean number of alleles = 1.01; eMOI = 1)
0.0
0.2
0.4
0.6
0.8
1.0
7907
9674
12243
15441
24710
26982
33640
43133
44595
50287
54827
64721
67489
74562
81132
87526
92164
93261
100236
104765
111288
113672
118844
127882
130632
139001
143020
145716
151535
153449
157614
165484
178560
182475
185264
190188
191493
202627
206308
212462
214187
220647
226528
231108
234628
240353
246142
247163
256576
259053
264031
267505
270238
274564
276606
285205
291380
296123
301517
304503
307891
313080
322153
326331
329094
335584
341498
344819
353283
354590
357841
364991
370151
382674
385196
389038
394419
403278
416631
419038
422331
430447
434704
445323
452117
456454
458944
461645
464987
466426
475191
478433
481392

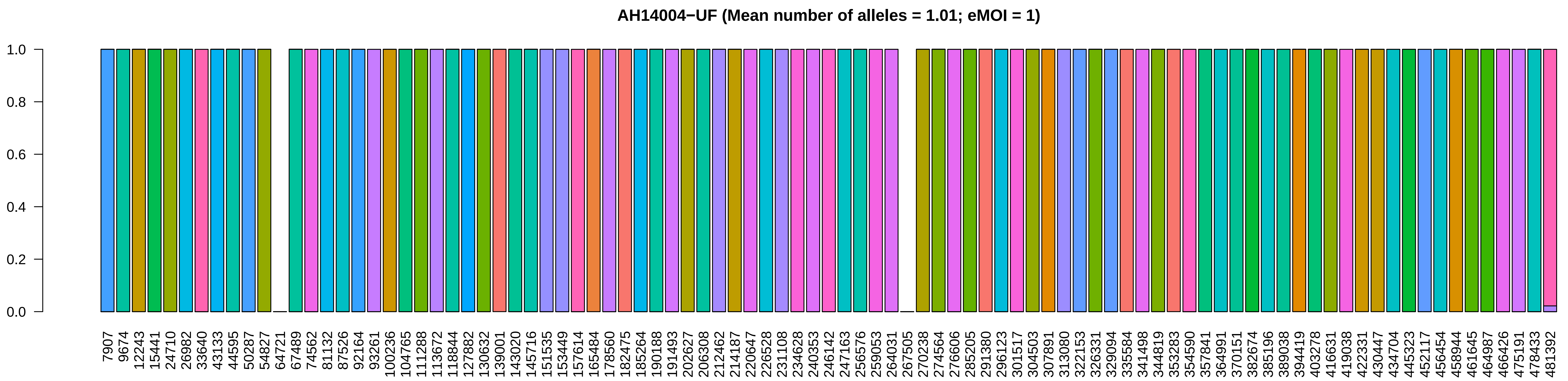
AH14004−UF (Mean number of alleles = 1.01; eMOI = 1)
1.0
0.8
0.6
0.4
0.2
0.0
7907 9674 12243 15441 24710 26982 33640 43133 44595 50287 54827 64721 67489 74562 81132 87526 92164 93261 100236 104765 111288 113672 118844 127882 130632 139001 143020 145716 151535 153449 157614 165484 178560 182475 185264 190188 191493 202627 206308 212462 214187 220647 226528 231108 234628 240353 246142 247163 256576 259053 264031 267505 270238 274564 276606 285205 291380 296123 301517 304503 307891 313080 322153 326331 329094 335584 341498 344819 353283 354590 357841 364991 370151 382674 385196 389038 394419 403278 416631 419038 422331 430447 434704 445323 452117 456454 458944 461645 464987 466426 475191 478433 481392

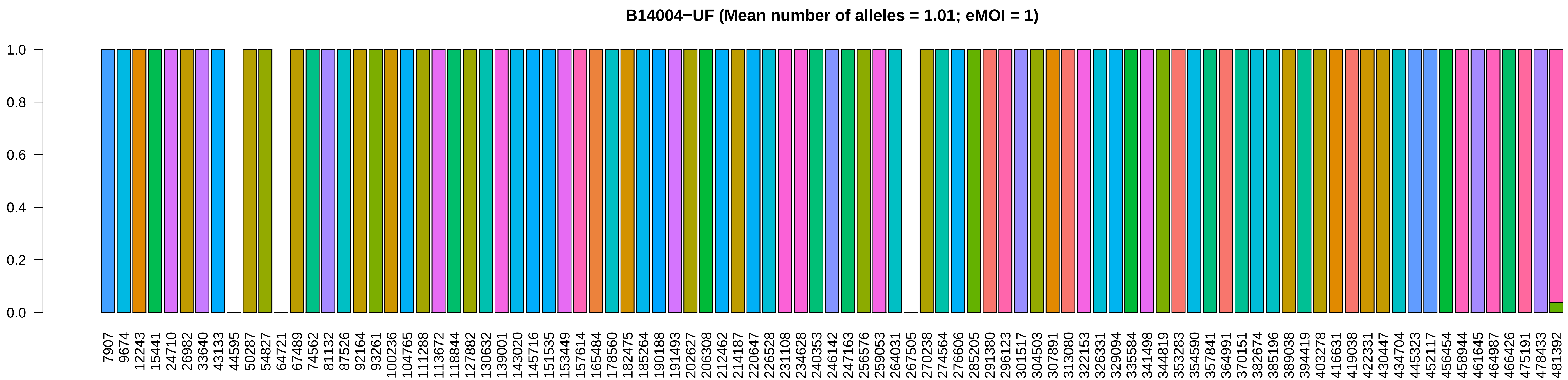

B14004−UF (Mean number of alleles = 1.01; eMOI = 1)
1.0
0.8
0.6
0.4
0.2
0.0
7907 9674 12243 15441 24710 26982 33640 43133 44595 50287 54827 64721 67489 74562 81132 87526 92164 93261 100236 104765 111288 113672 118844 127882 130632 139001 143020 145716 151535 153449 157614 165484 178560 182475 185264 190188 191493 202627 206308 212462 214187 220647 226528 231108 234628 240353 246142 247163 256576 259053 264031 267505 270238 274564 276606 285205 291380 296123 301517 304503 307891 313080 322153 326331 329094 335584 341498 344819 353283 354590 357841 364991 370151 382674 385196 389038 394419 403278 416631 419038 422331 430447 434704 445323 452117 456454 458944 461645 464987 466426 475191 478433 481392

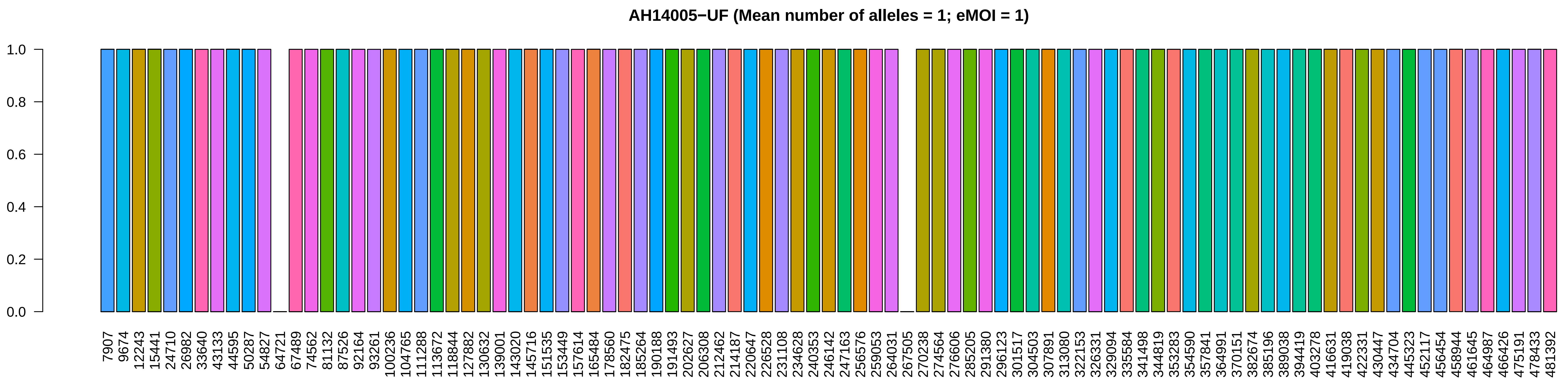
AH14005−UF (Mean number of alleles = 1; eMOI = 1)
1.0
0.8
0.6
0.4
0.2
0.0
7907
9674
12243
15441
24710
26982
33640
43133
44595
50287
54827
64721
67489
74562
81132
87526
92164
93261
100236
104765
111288
113672
118844
127882
130632
139001
143020
145716
151535
153449
157614
165484
178560
182475
185264
190188
191493
202627
206308
212462
214187
220647
226528
231108
234628
240353
246142
247163
256576
259053
264031
267505
270238
274564
276606
285205
291380
296123
301517
304503
307891
313080
322153
326331
329094
335584
341498
344819
353283
354590
357841
364991
370151
382674
385196
389038
394419
403278
416631
419038
422331
430447
434704
445323
452117
456454
458944
461645
464987
466426
475191
478433
481392

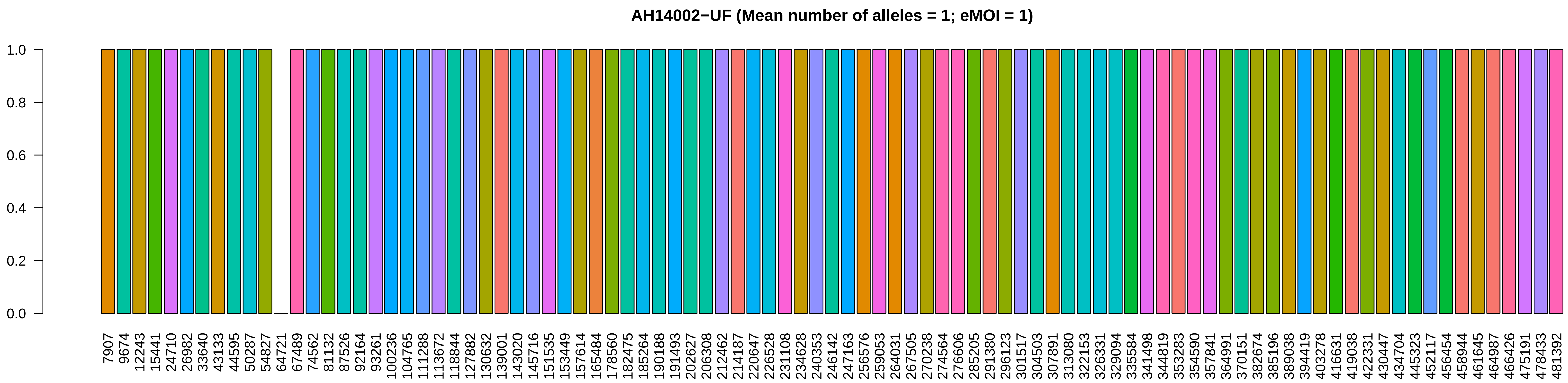

AH14002−UF (Mean number of alleles = 1; eMOI = 1)
1.0
0.8
0.6
0.4
0.2
0.0
7907
9674
12243
15441
24710
26982
33640
43133
44595
50287
54827
64721
67489
74562
81132
87526
92164
93261
100236
104765
111288
113672
118844
127882
130632
139001
143020
145716
151535
153449
157614
165484
178560
182475
185264
190188
191493
202627
206308
212462
214187
220647
226528
231108
234628
240353
246142
247163
256576
259053
264031
267505
270238
274564
276606
285205
291380
296123
301517
304503
307891
313080
322153
326331
329094
335584
341498
344819
353283
354590
357841
364991
370151
382674
385196
389038
394419
403278
416631
419038
422331
430447
434704
445323
452117
456454
458944
461645
464987
466426
475191
478433
481392

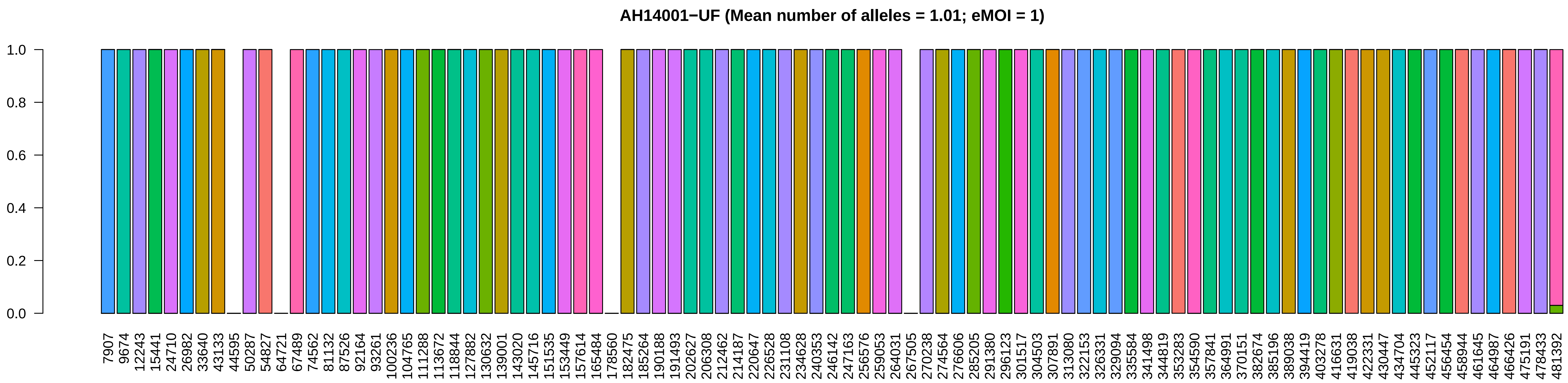
AH14001−UF (Mean number of alleles = 1.01; eMOI = 1)
1.0
0.8
0.6
0.4
0.2
0.0
7907 9674 12243 15441 24710 26982 33640 43133 44595 50287 54827 64721 67489 74562 81132 87526 92164 93261 100236 104765 111288 113672 118844 127882 130632 139001 143020 145716 151535 153449 157614 165484 178560 182475 185264 190188 191493 202627 206308 212462 214187 220647 226528 231108 234628 240353 246142 247163 256576 259053 264031 267505 270238 274564 276606 285205 291380 296123 301517 304503 307891 313080 322153 326331 329094 335584 341498 344819 353283 354590 357841 364991 370151 382674 385196 389038 394419 403278 416631 419038 422331 430447 434704 445323 452117 456454 458944 461645 464987 466426 475191 478433 481392

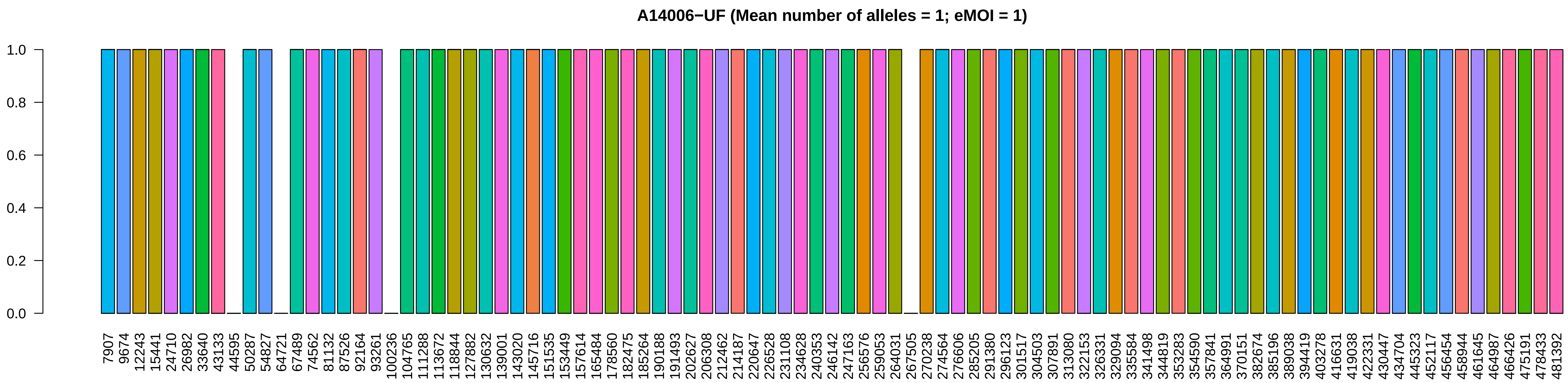
A14006−UF (Mean number of alleles = 1; eMOI = 1)
1.0
0.8
0.6
0.4
0.2
0.0
7907
9674
12243
15441
24710
26982
33640
43133
44595
50287
54827
64721
67489
74562
81132
87526
92164
93261
100236
104765
111288
113672
118844
127882
130632
139001
143020
145716
151535
153449
157614
165484
178560
182475
185264
190188
191493
202627
206308
212462
214187
220647
226528
231108
234628
240353
246142
247163
256576
259053
264031
267505
270238
274564
276606
285205
291380
296123
301517
304503
307891
313080
322153
326331
329094
335584
341498
344819
353283
354590
357841
364991
370151
382674
385196
389038
394419
403278
416631
419038
422331
430447
434704
445323
452117
456454
458944
461645
464987
466426
475191
478433
481392

A14007−UF (Mean number of alleles = 1; eMOI = 1)

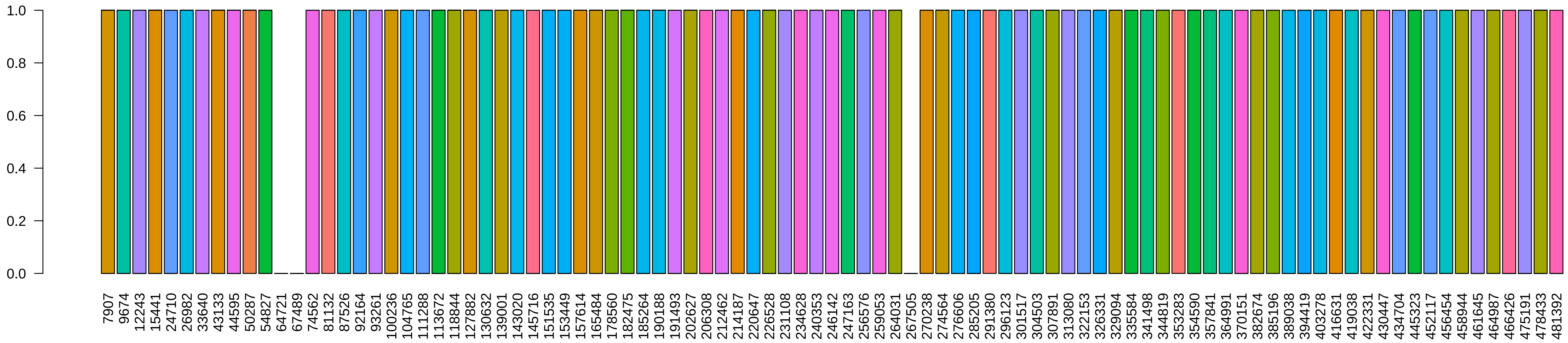

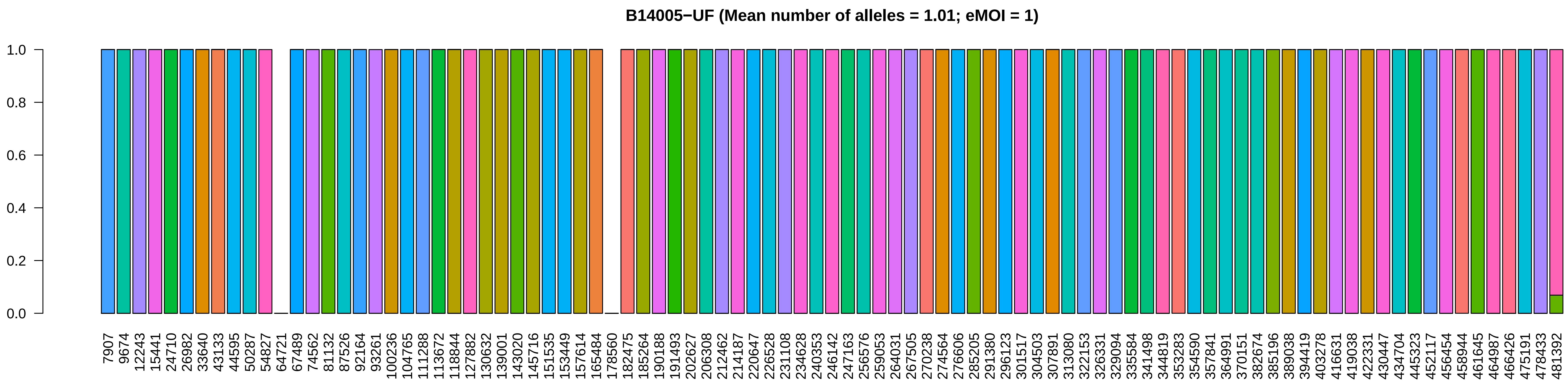
B14005−UF (Mean number of alleles = 1.01; eMOI = 1)
1.0
0.8
0.6
0.4
0.2
0.0
7907 9674 12243 15441 24710 26982 33640 43133 44595 50287 54827 64721 67489 74562 81132 87526 92164 93261 100236 104765 111288 113672 118844 127882 130632 139001 143020 145716 151535 153449 157614 165484 178560 182475 185264 190188 191493 202627 206308 212462 214187 220647 226528 231108 234628 240353 246142 247163 256576 259053 264031 267505 270238 274564 276606 285205 291380 296123 301517 304503 307891 313080 322153 326331 329094 335584 341498 344819 353283 354590 357841 364991 370151 382674 385196 389038 394419 403278 416631 419038 422331 430447 434704 445323 452117 456454 458944 461645 464987 466426 475191 478433 481392

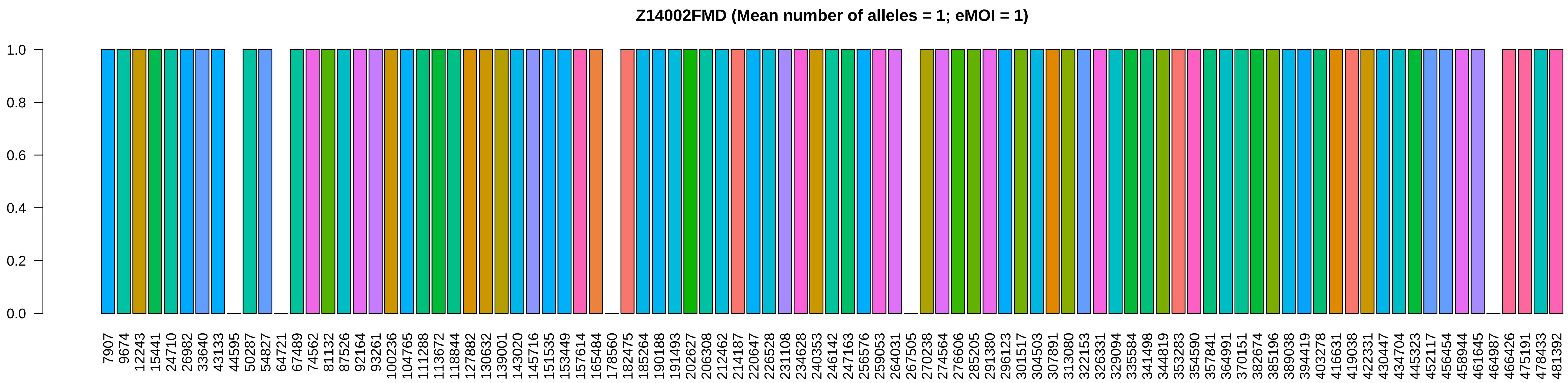
Z14002FMD (Mean number of alleles = 1; eMOI = 1)
1.0
0.8
0.6
0.4
0.2
0.0
7907
9674
12243
15441
24710
26982
33640
43133
44595
50287
54827
64721
67489
74562
81132
87526
92164
93261
100236
104765
111288
113672
118844
127882
130632
139001
143020
145716
151535
153449
157614
165484
178560
182475
185264
190188
191493
202627
206308
212462
214187
220647
226528
231108
234628
240353
246142
247163
256576
259053
264031
267505
270238
274564
276606
285205
291380
296123
301517
304503
307891
313080
322153
326331
329094
335584
341498
344819
353283
354590
357841
364991
370151
382674
385196
389038
394419
403278
416631
419038
422331
430447
434704
445323
452117
456454
458944
461645
464987
466426
475191
478433
481392

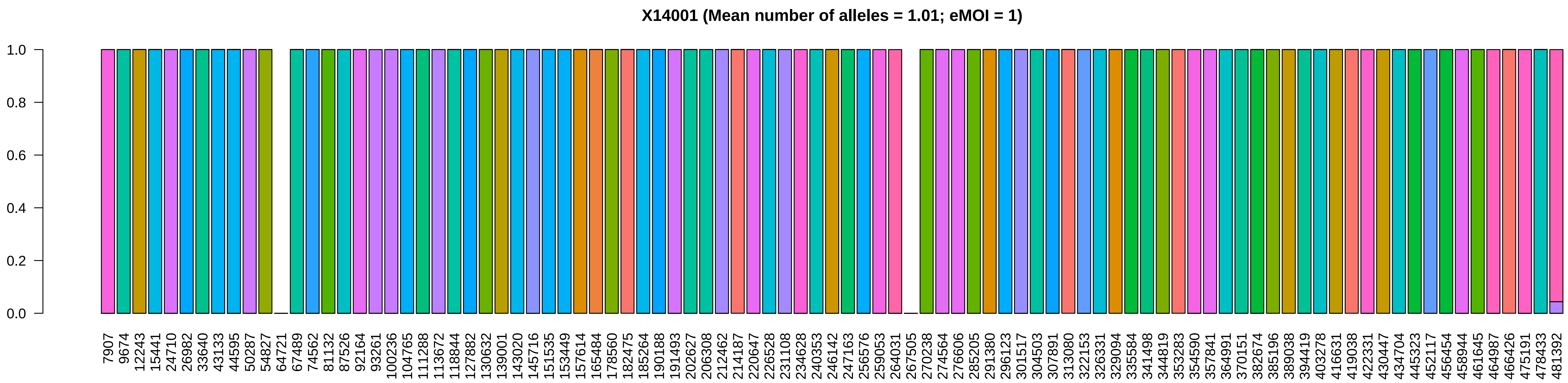
X14001 (Mean number of alleles = 1.01; eMOI = 1)

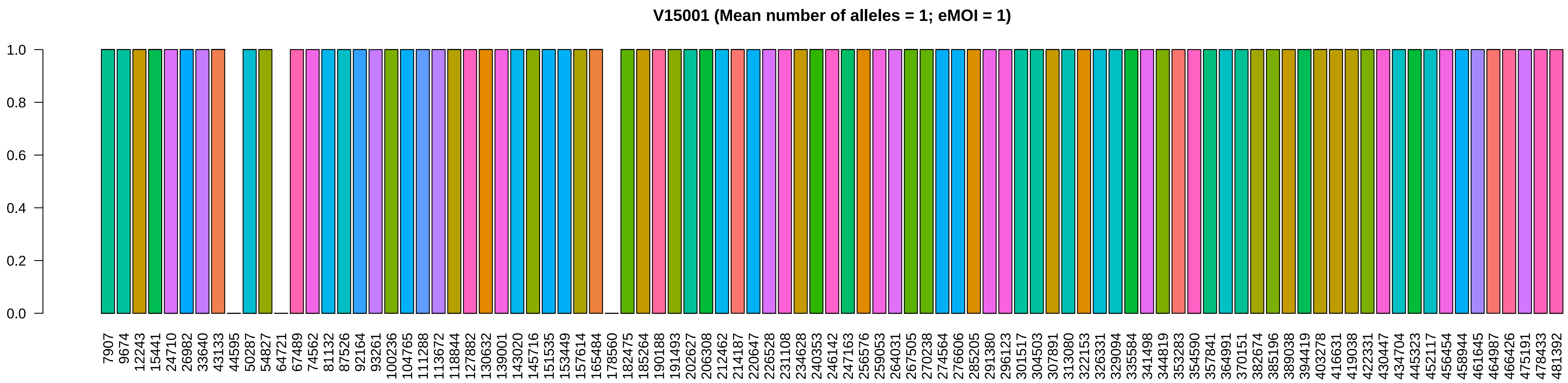
V15001 (Mean number of alleles = 1; eMOI = 1)
1.0
0.8
0.6
0.4
0.2
0.0
7907 9674 12243 15441 24710 26982 33640 43133 44595 50287 54827 64721 67489 74562 81132 87526 92164 93261 100236 104765 111288 113672 118844 127882 130632 139001 143020 145716 151535 153449 157614 165484 178560 182475 185264 190188 191493 202627 206308 212462 214187 220647 226528 231108 234628 240353 246142 247163 256576 259053 264031 267505 270238 274564 276606 285205 291380 296123 301517 304503 307891 313080 322153 326331 329094 335584 341498 344819 353283 354590 357841 364991 370151 382674 385196 389038 394419 403278 416631 419038 422331 430447 434704 445323 452117 456454 458944 461645 464987 466426 475191 478433 481392

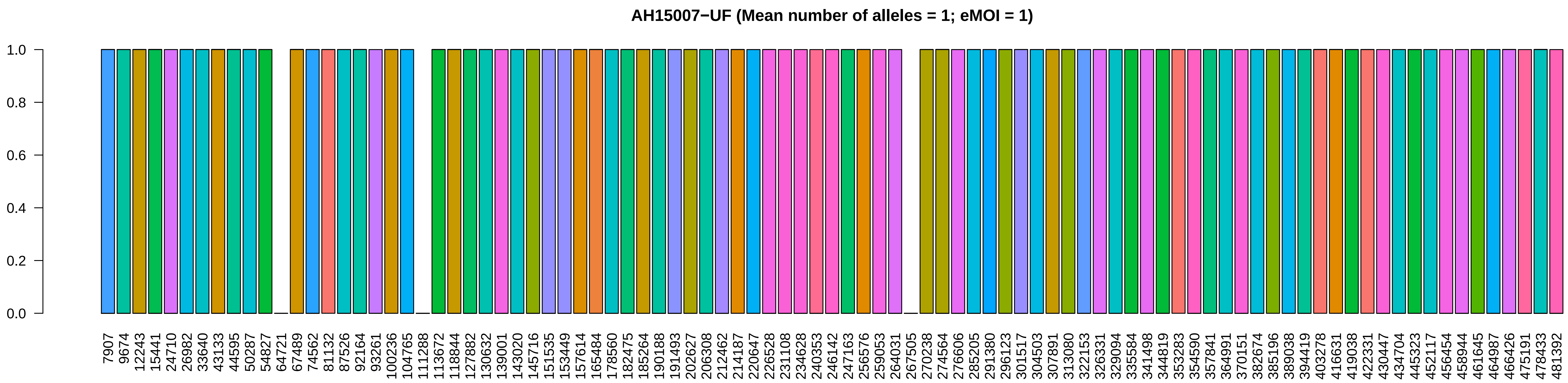
AH15007−UF (Mean number of alleles = 1; eMOI = 1)
0.0
0.2
0.4
0.6
0.8
1.0
7907 9674 12243 15441 24710 26982 33640 43133 44595 50287 54827 64721 67489 74562 81132 87526 92164 93261 100236 104765 111288 113672 118844 127882 130632 139001 143020 145716 151535 153449 157614 165484 178560 182475 185264 190188 191493 202627 206308 212462 214187 220647 226528 231108 234628 240353 246142 247163 256576 259053 264031 267505 270238 274564 276606 285205 291380 296123 301517 304503 307891 313080 322153 326331 329094 335584 341498 344819 353283 354590 357841 364991 370151 382674 385196 389038 394419 403278 416631 419038 422331 430447 434704 445323 452117 456454 458944 461645 464987 466426 475191 478433 481392
